# A Late Paleocene age for Greenland’s Hiawatha impact structure

**DOI:** 10.1126/sciadv.abm2434

**Published:** 2022-03-09

**Authors:** Gavin G. Kenny, William R. Hyde, Michael Storey, Adam A. Garde, Martin J. Whitehouse, Pierre Beck, Leif Johansson, Anne Sofie Søndergaard, Anders A. Bjørk, Joseph A. MacGregor, Shfaqat A. Khan, Jérémie Mouginot, Brandon C. Johnson, Elizabeth A. Silber, Daniel K. P. Wielandt, Kurt H. Kjær, Nicolaj K. Larsen

**Affiliations:** 1 Department of Geosciences, Swedish Museum of Natural History, SE-104 05 Stockholm, Sweden.; 2 Globe Institute, University of Copenhagen, Øster Voldgade 5-7, 1350 Copenhagen K, Denmark.; 3 Quadlab, Natural History Museum of Denmark, Øster Voldgade 5-7, 1350 Copenhagen K, Denmark.; 4 Geological Survey of Denmark and Greenland, Øster Voldgade 10, 1350 Copenhagen K, Denmark.; 5 Univ. Grenoble Alpes, CNRS, IPAG, 38000 Grenoble, France.; 6 Department of Geology, Lund University, Sölvegatan 12, 223 62 Lund, Sweden.; 7 Department of Geoscience, Aarhus University, Høegh Guldbergs Gade 2, 8000 Aarhus, Denmark.; 8 Laboratory for Ion Beam Physics, ETH Zürich, Otto-Stern-Weg 5, 8093 Zürich, Switzerland.; 9 Department of Geoscience and Natural Resource Management, University of Copenhagen, Øster Voldgade 10, 1350 Copenhagen K, Denmark.; 10 Cryospheric Sciences Lab, NASA Goddard Space Flight Center, Greenbelt, MD 20771, USA.; 11 Department of Geodesy, National Space Institute, Technical University; of Denmark, Kongens Lyngby, Denmark.; 12 Institut des Géosciences de l’Environnement, CNRS, Université Grenoble Alpes, Grenoble, France.; 13 Department of Earth System Science, University of California, Irvine, Irvine, CA 92617, USA.; 14 Department of Earth, Atmospheric, and Planetary Sciences, Purdue University, West Lafayette, IN 47907, USA.; 15 Department of Physics and Astronomy, Purdue University, West Lafayette, IN 47907, USA.; 16 Department of Earth Sciences, Western University, London, ON N6A 5B7, Canada.; 17 The Institute for Earth and Space Exploration, Western University, London, ON N6A 3K7, Canada.

## Abstract

The ~31-km-wide Hiawatha structure, located beneath Hiawatha Glacier in northwestern Greenland, has been proposed as an impact structure that may have formed after the Pleistocene inception of the Greenland Ice Sheet. To date the structure, we conducted ^40^Ar/^39^Ar analyses on glaciofluvial sand and U-Pb analyses on zircon separated from glaciofluvial pebbles of impact melt rock, all sampled immediately downstream of Hiawatha Glacier. Unshocked zircon in the impact melt rocks dates to ~1915 million years (Ma), consistent with felsic intrusions found in local bedrock. The ^40^Ar/^39^Ar data indicate Late Paleocene resetting and shocked zircon dates to 57.99 ± 0.54 Ma, which we interpret as the impact age. Consequently, the Hiawatha impact structure far predates Pleistocene glaciation and is unrelated to either the Paleocene-Eocene Thermal Maximum or flood basalt volcanism in east Greenland. However, it was contemporaneous with the Paleocene Carbon Isotope Maximum, although the impact’s exact paleoenvironmental and climatic significance awaits further investigation.

## Teaser

The Hiawatha impact structure is dated to 58 Ma by ^40^Ar/^39^Ar analysis of sand and U-Pb analysis of shocked zircon.

## INTRODUCTION

**Fig. 1.**
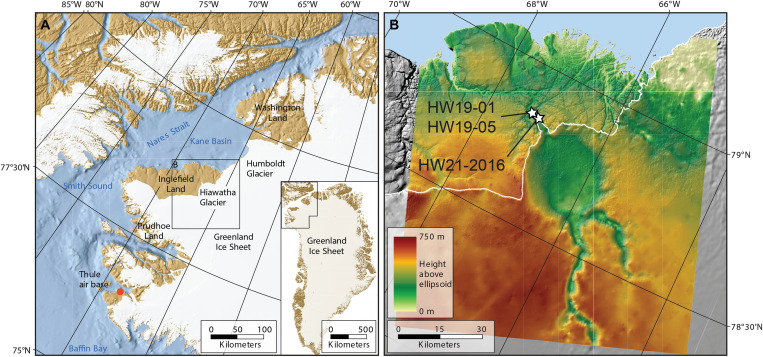
Location and geomorphological setting of Hiawatha Glacier, northwest Greenland. (**A**) Regional view of northwest Greenland. (**B**) Bedrock topography map showing the Hiawatha structure, and sampling locations of glaciofluvial sediment for ^40^Ar/^39^Ar analysis (HW21-2016) and clasts of impact melt rock for zircon U-Pb analysis (HW19-01 and HW19-05). Bed topography based on NASA and 2016 Alfred Wegener Institute (AWI) airborne radar-sounding data. Samples HW19-01 and HW19-05 are from the same location on a wide riverbank 4 km downstream of the terminus of Hiawatha Glacier. White line represents the present-day margin of the Greenland Ice Sheet. Figure was modified after (*1*).

The ~31-km-wide Hiawatha structure, located beneath Hiawatha Glacier in northwestern Greenland (Fig. 1), was proposed as an impact structure on the basis of (i) airborne radar surveys revealing a relatively flat, circular depression with an elevated rim and a subtle central uplift, (ii) structures in the bedrock along the ice margin striking tangential to the subglacial rim, and (iii) shocked quartz, and other grains interpreted to be impact related, in glaciofluvial sediment from the largest river draining the structure (*1*–*3*).

To contextualize an impact structure within the geologic record requires precise knowledge of its age, but available age constraints for the Hiawatha structure are only tentative. The bedrock under the structure is likely a continuation of the highly metamorphosed 1.95– to 1.75–billion year (Ga) (*4*) bedrock of the Ellesmere-Inglefield mobile belt that is exposed in Inglefield Land immediately adjacent to Hiawatha Glacier, which provides a maximum constraint on the structure’s age (*1*). Further age estimates have been based on indirect constraints, such as estimates of erosion rates in Greenland and the structure’s apparent relationship to the Greenland Ice Sheet (*1*). The structure has a rim-to-floor depth of 320 ± 70 m and a dissected central uplift that is up to 50 m high and whose peaks are up to ~8 km apart (*1*)*.* The depth of the structure and the height of the central uplift are muted compared to that predicted for a fresh, subaerial terrestrial crater of the same diameter (predicted depth of ~800 m) (*5*). This could result from slow erosion over a long period or faster erosion over a shorter period. Reported fluvial and subglacial erosion rates spanning a range of ~10 to 10,000 m million years^−1^ (Ma^−1^) (~10^−5^ to 10^−2^ m year^−1^) (*6*–*9*) mean that the ~500-m erosion of the crater rim corresponds to a minimum period of erosion of ~50 thousand years (ka) and a maximum period of ~50 Ma (*1*). Radar evidence of active subglacial erosion at present and active sediment deposition at the glacier front were interpreted to favor a faster subglacial erosion rate and hence potentially a younger age (*1*). Anomalous radiostratigraphy of the ice of Hiawatha Glacier relative to the rest of the Greenland Ice Sheet was interpreted to have potentially resulted from an impact through thick ice or subsequent impact-related heating affecting ice flow (*1*), also provisionally suggesting an impact after the 2.6-Ma (*10*) inception of the Greenland Ice Sheet.

Given the tentative nature of these previous age constraints, here, we undertake dating of the structure using detrital materials sampled immediately downstream thereof. We report both ^40^Ar/^39^Ar analysis of the impact-related glaciofluvial sand previously described in (*1*) and U-Pb analysis of shocked zircon from recently collected, and previously unreported, pebble-sized glaciofluvial clasts of impact melt rock.

## RESULTS

**Fig. 2.**
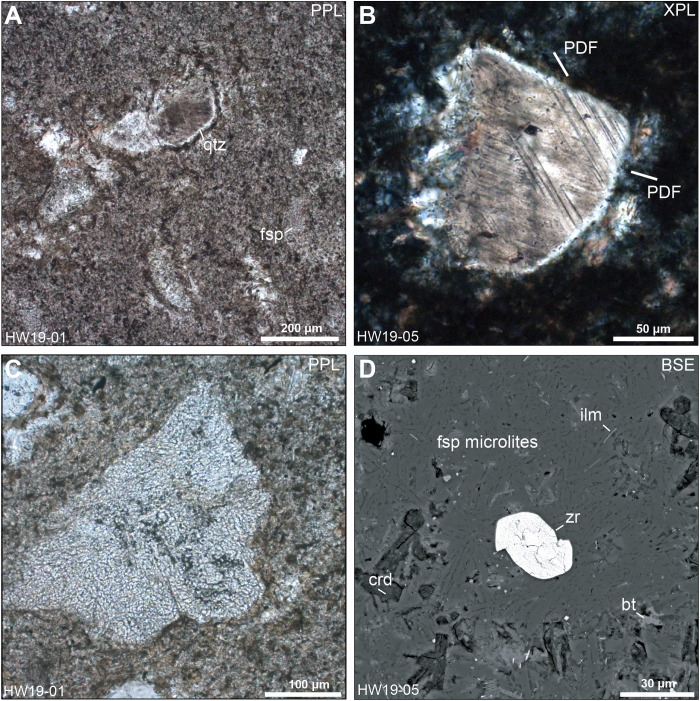
Images of impact melt rocks from the Hiawatha structure. (**A**) Feldspathic microlitic matrix with clasts of toasted quartz (qtz) and checkerboard feldspars (fsp). (**B**) Lightly toasted quartz fragment with two sets of PDFs that are considered unequivocal evidence of shock metamorphism. (**C**) Checkerboard feldspar. (**D**) Petrographic context of a granular and porous zircon (zr) grain (see Fig. 3 for high-resolution images of granular and porous zircon grains separated from the rock) in the feldspathic (fsp) matrix of impact melt rock, with accessory biotite (bt), ilmenite (ilm), and altered cordierite (crd). In contrast to zircon grains like this one that were in direct contact with the impact melt, zircon grains within clasts in impact melt rock do not display porous and granular textures. BSE, backscattered electrons; PPL, plane-polarized light; XPL, cross-polarized light.

**Fig. 3.**
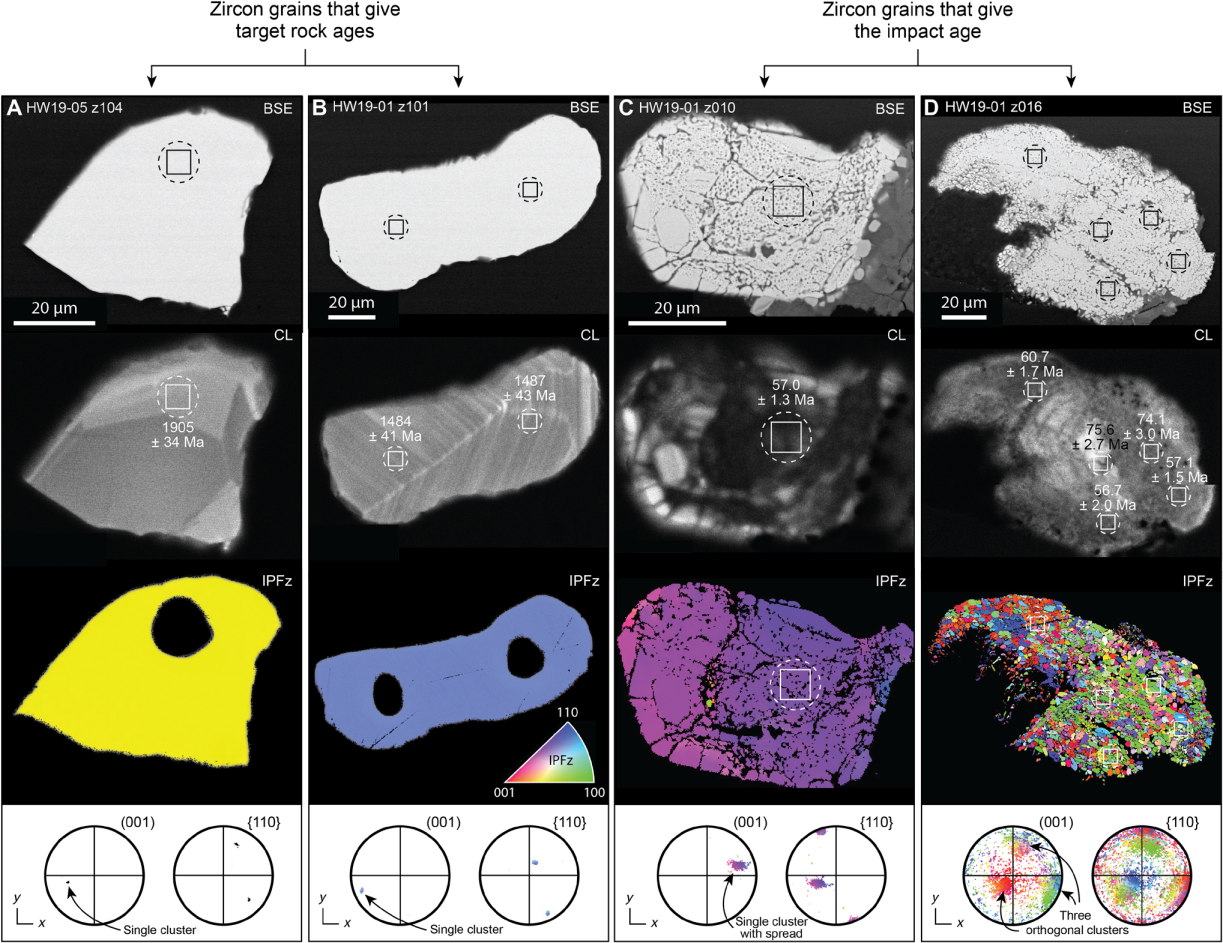
Imaging and microstructural characterization of zircon from the Hiawatha structure. (**A** and **B**) Pristine zircon grains that record target rock ages. These grains retain initial growth zoning visible in CL and show no evidence for impact-related deformation or recrystallization. Color in the inverse pole figure (IPF) images reflects crystallographic orientation [legend in (B) applies to all grains]. Minimal color variation in (A) and (B) indicates that these grains have not undergone appreciable deformation. (**C**) A porous zircon grain that records the impact age. The subtle variation in color across the crystal reflects up to ~20° of intra-grain misorientation resulting from crystal-plastic deformation. (**D**) A granular zircon grain that records the impact age. The different colors, as well as three clusters visible in the (001) pole figure, reflect distinct orthogonal crystallographic relationships between neoblasts in the recrystallized zircon grain. This is indicative of so-called “former reidite in granular neoblastic” (FRIGN) zircon (*16*,*17*), which has only been documented in impact-related rocks. Dashed circles represent the U-Pb analysis pits, and the squares represent the areas that were sampled for U-Pb geochronology after the secondary ion beam passed through the field aperture (see Materials and Methods). The black ellipses in the microstructural images in (A) and (B) correspond to the U-Pb analysis pits (microstructural analysis on these grains was performed after U-Pb analysis, whereas microstructural analysis on all other grains was performed before U-Pb analysis). Ages less than 1200 Ma are ^206^Pb/^238^U ages; those above 1200 Ma are ^207^Pb/^206^Pb ages. CL, cathodoluminescence.

The glaciofluvial sand sample analyzed here (HW21-2016; 78.83305°N, 67.13653°W) was collected in 2016 and reported on in (*1*–*3*). It is well-sorted, fine-grained sand that was collected from an active floodplain ~300 m from the terminus of Hiawatha Glacier and ~5 km from the inferred edge of the Hiawatha structure (Fig. 1). Historical aerial photographs and satellite images show that the floodplain began to build up after 2010, with sediments originating from within the Hiawatha structure (*1*). 50 sand grains from sample HW21-2016, which contains impact-diagnostic features, such as planar deformation features (PDFs) in quartz, underwent ^40^Ar/^39^Ar laser step-heating analysis. We also analyzed two pebble-sized clasts collected in 2019 from a wide riverbank ~4 km downstream from the terminus of Hiawatha Glacier and less than 10 km from the edge of the Hiawatha structure (HW19-01 and HW19-05; 78.84170°N, 67.29410°W; Fig. 1). The most likely provenance of the pebbles is also the proximal part of Hiawatha structure via subglacial and glaciofluvial transport (fig. S1). HW19-01 and HW19-05 are clast-rich impact melt rocks (Fig. 2 and fig. S2). Both samples have a dark gray, aphanitic, hemicrystalline melt matrix dominated by lath-like plagioclase feldspar microlites. The samples contain silicate clasts with impact-diagnostic shock features (Fig. 2, A and B), i.e., quartz grains that are generally strongly toasted (i.e., have a brown, nonpleochroic grainy appearance in plane- and cross-polarized light) and display multiple sets of PDFs (Fig. 2, A and B, and fig. S2, B and C). Further, most of the plagioclase feldspar clasts have checkerboard textures (Fig. 2C and fig. S2, E and F), which are likely related to impact-induced disequilibrium melting (*11*–*13*). Zircon grains within clasts in the impact melt rocks retain original growth zoning and do not appear shocked (Fig. 3, A and B), whereas zircon grains in contact with the feldspathic matrix (Fig. 2D) generally display porous and granular textures (Fig. 3, C and D). To complement the ^40^Ar/^39^Ar data for sediment sample HW21-2016, after the 2019 collection of pebble-size clasts of impact melt rock, aliquots of both HW19-01 and HW19-05 were crushed and zircon grains were extracted for U-Pb analysis.

### ^40^Ar/^39^Ar geochronology of glaciofluvial sand

The 50 grains that underwent ^40^Ar/^39^Ar step-heating analysis were hand-picked with the aim to (i) analyze the diversity of grain types in the sample and (ii) analyze a large number of grains that may represent fragments of solidified impact melt. For the latter, we selected grains with a very fine-grained to aphanitic matrix, occasionally with perlitic fractures, typically with a visible microspherulitic structure, and as few mineral fragments as possible. To maximize the analytical mass, only the largest, relatively rare grains at least 1 mm across were picked. The identification of melt grains and their descriptions are based on visual comparison with grains of known composition and microstructure previously documented in this sample in (*1*–*3*). Almost all grains can be assigned to four groups: (i) 20 grains have a greenish gray, yellow, or dark organic-rich matrix with feldspathic microspherulites about 10 to 50 μm across and fragments of quartz and feldspar (e.g., QL03, QL12, and QL29; one such grain, QL04, also has perlitic microfractures); (ii) 10 grains have a noncrystalline, glassy, or commonly schlieric matrix and mineral fragments (e.g., QL41); (iii) 6 grains have a hemicrystalline, presumably feldspathic matrix and numerous mineral fragments (e.g., QL05, QL18, and QL31); (iv) 3 grains have a dark, hemicrystalline, presumably feldspathic matrix and microlites presumably of pyroxene and ilmenite (e.g., QL16). Ten further grains have overlapping features between these groups or are dark without distinct features (e.g., QL25 and QL36). A unique and presumed sedimentary grain of pale, ellipsoidal to spherical silica ooids with nuclei of quartz fragments was also selected for analysis (QL01). A detailed petrographic description was not possible for each grain because they were too small to be mounted in thin section before analysis, but a description of each grain’s exterior is provided in the Supplementary Text, images of all analyzed grains are shown alongside ^40^Ar/^39^Ar spectra in fig. S3, and results from ^40^Ar/^39^Ar laser step-heating age experiments are reported in data S1.

**Fig. 4.**
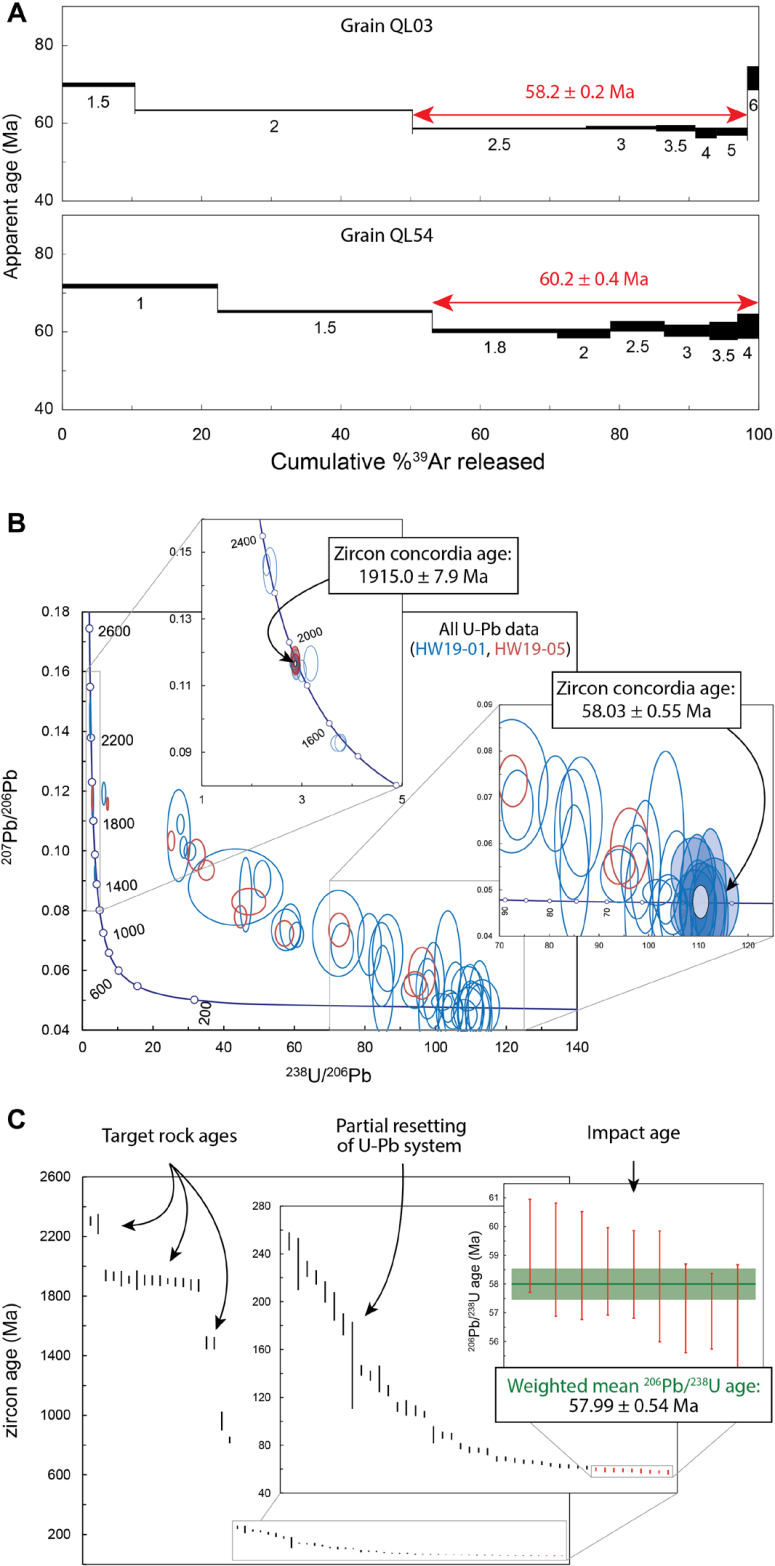
^40^Ar/^39^Ar and U-Pb data for the Hiawatha structure. (**A**) ^40^Ar/^39^Ar age spectra for the two grains that gave mini-plateau ages. Numbers beneath individual steps indicate laser power in watts. Mean square of weighted deviates (MSWD) and probability on mini-plateau age for QL03 are 1.73 and 0.14; those for QL54 are 1.04 and 0.39. (**B**) Tera-Wasserburg concordia diagrams showing zircon U-Pb data. MSWD and probability (of concordance and equivalence) of older concordia age are 0.99 and 0.47 (*n* = 11); those for younger concordia age are 0.96 and 0.50 (*n* = 9). (**C**) Ranked apparent zircon ages. Those less than 1200 Ma are ^206^Pb/^238^U ages; those above 1200 Ma are ^207^Pb/^206^Pb ages. MSWD and probability are 1.18 and 0.30 (*n* = 9). Uncertainties are 2σ.

Of the 50 grains that underwent ^40^Ar/^39^Ar step-heating analysis, 29 developed “U”- or “saddle”-shaped age spectra (*14*), with minimum ages occurring at medium-to-high laser power (fig. S3), 2 developed mini-plateaus (based on 47 and 48% of ^39^Ar released, respectively, and a minimum of five consecutive steps; Fig. 4A), and none developed statistically robust plateaus [defined as at least five consecutive steps that comprised at least 50% of the ^39^Ar released; (*15*)]. Of the 29 saddle-shaped spectra, the majority (23) gave minimum apparent ages in the Paleocene, and the 2 mini-plateaus gave Late Paleocene ages of 58.5 ± 0.2/0.3 Ma (internal/external error) [grain QL03; mean square of weighted deviates (MSWD) = 1.73; probability = 0.14; *n* = 5] and 60.2 ± 0.4/0.5 Ma (grain QL54; MSWD = 1.04; probability = 0.39; *n* = 6) (Fig. 4A; see also inverse isochrons in fig. S4). The two mini-plateau ages do not agree with each other, but given that 23 of 29 saddle-shaped spectra give minimum apparent ages in the Paleocene, they suggest that the grains record a Late Paleocene resetting event. One reason for the complex age spectra and lack of statistically robust plateaus may be that the analyzed grains were composed of multiple phases, including fragments of minerals from the target rock (fig. S3), which are likely to have undergone variable degrees of resetting and have thus retained variable amounts of inherited radiogenic ^40^Ar (*14*).

### U-Pb geochronology of zircon separated from impact melt rocks

Zircon grains were separated from the two clast-rich impact melt rocks, mounted in epoxy, ground to expose their midsections, polished, and imaged in backscattered electron (BSE) and cathodoluminescence (CL) modes on a scanning electron microscope (SEM). Images of all analyzed grains are presented in fig. S5. Both samples contain (i) pristine, apparently undeformed grains (Fig. 3, A and B), (ii) partially to entirely porous grains (Fig. 3C), and (iii) partially to entirely granular grains with micrometer-scale neoblasts (Fig. 3D). Microstructural characterization by electron backscatter diffraction (EBSD) analysis was performed on two pristine, apparently undeformed grains (Fig. 3, A and B) and eight grains that displayed porous or granular textures (Fig. 3, C and D, and figs. S6 to S13). Reidite, a high-pressure polymorph of zircon, was not detected. However, neoblasts in the three pervasively granular zircon grains preserve distinct crystallographic relationships (e.g., Fig. 3D) that are indicative of so-called “former reidite in granular neoblastic” (FRIGN) zircon (*16*–*17*). The interpretation that these recrystallization textures indicate the former presence of reidite is disputed (*18*), but they have only been documented at impact structures; thus, we consider them related unequivocally to an impact event. U-Pb analyses by secondary ion mass spectrometry (SIMS) targeted the three grain types described above (data S2 and S3). Uncertainties are presented at the 2σ level unless otherwise stated.

**Fig. 5.**
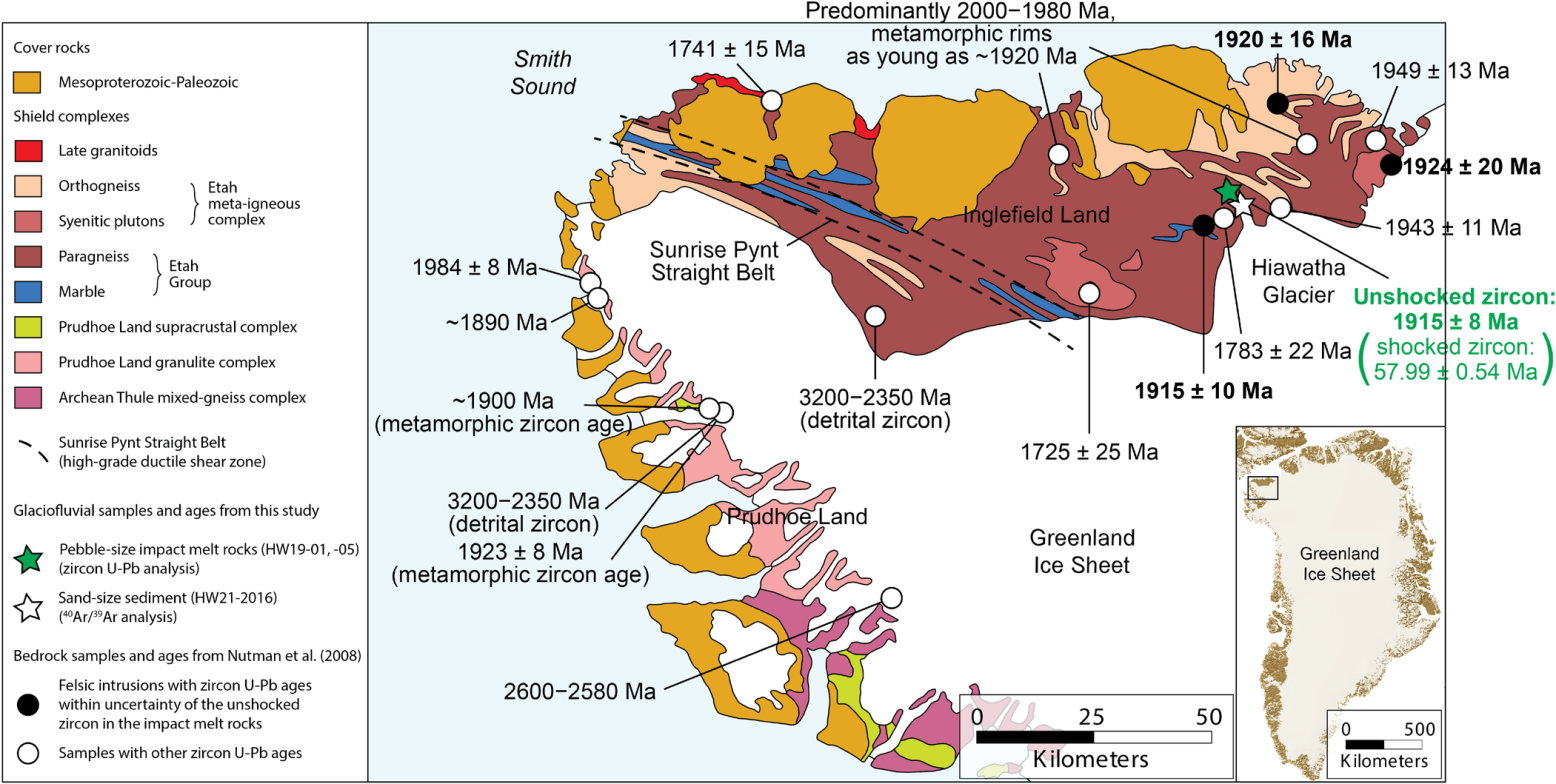
Geological map of Inglefield Land and Prudhoe Land, northwest Greenland. Previously published zircon U-Pb ages for bedrock samples (*4*) are shown in black text, and the age of unshocked zircon in clasts of impact melt rock sampled 4 km downstream from the terminus of Hiawatha Glacier (present study) is shown in green text. The dominant age of unshocked zircon in the impact melt rock samples (1915 ± 8 Ma) is indistinguishable from the zircon U-Pb ages of three felsic igneous intrusions in the vicinity of Hiawatha Glacier (bold text), supporting a local origin for the clasts of impact melt rock. Map was modified after (*4*).

Seventeen analyses were performed on 15 undeformed zircon grains from the two impact melt rock samples. Twelve analyses cluster at 1915 ± 8 Ma (Fig. 4, B and C), 1 analysis gave a date of approximately 1915 Ma but appears to record minor Pb loss, and 4 analyses date two grains to ~2300 and ~ 1485 Ma, respectively. The 1915 ± 8 Ma age agrees with known geological events and zircon ages from the bedrock of Inglefield Land, adjacent to Hiawatha Glacier (*4*). At 1930 to 1920 Ma, the Paleoproterozoic rocks of the so-called Central terrane (*4*) that occupies most of Inglefield Land were (i) exposed to high-grade metamorphism and (ii) intruded by felsic igneous rocks (*4*). Specifically, three felsic intrusions of the Etah meta-igneous complex of Inglefield Land gave zircon crystallization ages (*4*) that are indistinguishable from the 1915 ± 8 Ma age of unshocked zircon studied here (Fig. 5). These include (i) a heavily folded syenite sampled approximately 35 km northwest of Hiawatha Glacier, which dates to 1920 ± 16 Ma (*4*); (ii) a monzogranite sampled from the major pluton (Humboldt pluton) bordering the Greenland Ice Sheet less than 10 km north of Hiawatha Glacier, which dates to 1924 ± 20 Ma (*4*); and (iii) an undeformed granite sampled approximately 5 km west of Hiawatha Glacier (and 5 km south of where the clasts of impact melt rocks studied here were collected), which dates to 1915 ± 10 Ma (*4*) (Fig. 5).

We interpret the modal age of the unshocked zircon in these impact melt rocks being indistinguishable from that of voluminous local felsic intrusions as strongly supporting a local origin for the impact melt rocks. Further, we note that these intrusions have not been reported in other areas of northwestern Greenland, such as Washington Land to the north and Prudhoe Land to the south. In this context, the two unshocked zircon grains in the melt rocks that do not date crystallization at ~1915 Ma but instead gave ages of ~2300 and ~1485 Ma may represent (i) incorporation of additional target rock lithologies into the impact melt during the impact event or, (ii) in the case of the older age, inherited xenocrystic zircon in the intrusive felsic target rock.

Forty-five analyses on 28 porous and granular zircon grains from the two samples define a discordant distribution between ~1915 and ~58 Ma, consistent with a Late Paleocene impact into Paleoproterozoic target rocks. A population of concordant data points at the lower end of this distribution defines a plateau of ranked ^206^Pb/^238^U dates (Fig. 4C). The lack of dates that are younger than the 58 Ma plateau in zircon ^206^Pb/^238^U dates indicates that the grains have not suffered appreciable post-impact Pb loss (Fig. 4, B and C). The youngest nine data points are statistically indistinguishable, giving a robust concordia age and a weighted mean ^206^Pb/^238^U age of 57.99 ± 0.54 Ma (MSWD = 1.18; probability = 0.30; *n* = 9) (Fig. 4C). The U-Pb age is within uncertainty of the younger of the two ^40^Ar/^39^Ar mini-plateau ages, 58.5 ± 0.3 Ma yielded by grain QL03. Given the tight clustering of zircon dates at ~58 Ma, in contrast to the complex ^40^Ar/^39^Ar spectra lacking robust plateau ages based on >50% of ^39^Ar released, we consider the 57.99 ± 0.54 Ma zircon U-Pb age to be the most robust estimate for the age of the impact structure.

## DISCUSSION

### A new age for the Hiawatha structure

Two impact melt rock samples, collected in 2019 from a riverbank less than 10 km downstream from the edge of the Hiawatha structure, contain unshocked zircon with ages indistinguishable from voluminous local felsic intrusions adjacent to—and presumably also under—Hiawatha Glacier. Shocked zircon in the same samples give a robust ~58-Ma U-Pb age, which agrees with the younger of two ^40^Ar/^39^Ar mini-plateau ages from the 2016 sand sample recovered even closer to the structure. In sum, these data indicate sample provenance from a Late Paleocene impact event that occurred somewhere upstream but sufficiently proximal that the target geology is indistinguishable geochronologically from that exposed locally where the samples were recovered. Given existing geomorphic evidence for an eroded complex impact structure beneath Hiawatha Glacier, whose apparent rim is breached by the subglacial channel that ultimately becomes the subaerial river channel from which our detrital samples were recovered (*1*), the simplest interpretation of our observations—which we explicitly accept for the remainder of the discussion—is that the Hiawatha structure is a relatively large impact structure that formed in the Late Paleocene.

When the Hiawatha structure was first proposed as an impact structure, it was also suggested that it post-dated the Pleistocene inception of the Greenland Ice Sheet at ~2.6 Ma (*1*). The ~58-Ma age for the structure indicates that it formed long before the inception of the ice sheet, and that it is unrelated to the onset of the Younger Dryas cold period ~12,900 years ago as has been speculated (*19*). The new age compels reevaluation of previous observations of the Hiawatha structure that were tentatively interpreted as supporting a much younger impact age. These include (i) the crater’s morphology, (ii) the lack of known ejecta related to the impact, (iii) the presence of organic carbon in apparently impact-related sand and particles of charcoal in the outwash plain in front of the structure, and (iv) the complex radiostratigraphy of Hiawatha Glacier.

Although the Hiawatha structure retains fundamental elements of a complex crater morphology, including a raised rim, it is relatively shallow and exhibits a smaller central uplift than expected, i.e., its morphology is muted compared to that expected for a young, subaerial impact (*1*,*20*). This was hypothesized to be a result of either (i) long-term and ongoing fluvial and subglacial erosion of an older crater formed by an impact into ice-free bedrock or (ii) a relatively recent impact into thick ice resulting in an altered final crater morphology (*1*, *20*). Impact simulations by Silber *et al.* (*20*) demonstrated that the present morphology of the Hiawatha structure alone could not distinguish between the two possibilities such that a Pleistocene age was possible. However, the Late Paleocene age reported here naturally explains the structure’s muted morphology as a result of long-term erosion, with its rim and porous peak ring (*21*) having been preferentially eroded, as well as the absence of impact ejecta in Greenland’s Pleistocene rock and ice records. Moreover, the age indicates that since 58 Ma mean erosion rates in this area of northwestern Greenland have been at the lower end of the reported range considered in (*1*). If we assume that the crater’s present rim-to-floor depth of 320 ± 70 m reflects ~500 m of erosion, this corresponds to a mean erosion rate of less than 10 m Ma^−1^. However, as the structure has not been drilled, it is not certain to what degree its present morphology reflects differential erosion across the feature or sediment infill of the crater floor, and the erosion rate remains an approximation only and likely an upper bound. Despite its imprecision, this relatively low erosion rate for a major subglacial structure in northwestern Greenland has important implications for the understanding of other major subglacial geomorphological features found beneath the ice sheet in recent years, including large valleys (*22*–*24*), a second possible impact structure (*25*), and a fault-bounded lake (*26*). These other features may thus also be older than previously assumed and unrelated to the inception of the Greenland Ice Sheet. In particular, slower erosion suggests that large subglacial valleys represent a long-lasting river system that delivered sediments offshore since the Mesozoic Era, and that they only were reshaped by glacial erosion during the Pliocene and Pleistocene epochs (*27*).

Pebble-sized charcoal particles (with cellular structures indicative of conifer wood) and apparently impact-related sand grains rich in organic carbon have been found in Hiawatha Glacier’s glaciofluvial outwash (*2*, *3*). These were assumed to have been derived from organic material in Early Pleistocene deposits and were thus interpreted to support a young impact age (*2*). However, the new age for the Hiawatha structure indicates that—if these materials are related to the impact—they must instead date to the Paleocene or earlier. Abundant Late Paleocene plant fossils in the Arctic (e.g., on Ellesmere Island, across Nares Strait from Inglefield Land) point to widespread high-altitude coniferous forests at this time (*28*), providing a plausible source of organic material in impact-related sediments at Hiawatha Glacier.

The ~58-Ma age for the structure shows that the disrupted radiostratigraphy of Hiawatha Glacier (*1*) is unrelated to an impact through thick ice or subsequent impact-related heating of the structure. Two alternative explanations considered in (*1*) remain possible. First, the subglacial structure is a basin for which there are two primary inlets through its southeast rim and one primary outlet through its northwest rim (Fig. 1B). Water could accumulate there subglacially—as occurs presently in the sediment ~10 m beneath the ice (*29*)—and then outburst catastrophically, which would consequently disrupt ice flow into the structure. However, this scenario would require an unusually large volume of subglacial water to have accumulated there, which is not clearly supplied by apparent basal melting farther upstream (*30*). Second, the early Holocene collapse of the Nares Strait ice bridge that formerly connected the Innuitian and Greenland Ice Sheets likely disrupted ice flow regionally in northwestern Greenland (*30*). A Late Pleistocene ice-flow disturbance of unknown origin is exposed in regularly folded margin-exposed ice stratigraphy for a 35-km stretch immediately southwest of Hiawatha Glacier (*31*). This regular folding appears to be unique in Greenland and is coherent with Hiawatha Glacier’s disrupted radiostratigraphy (*1*). While we cannot yet distinguish between these two mechanisms, we favor the latter, because it likely affected a larger region, while the former cannot clearly explain why the stratigraphy disturbance is focused southwest of Hiawatha Glacier.

### The Hiawatha impact in the context of the Late Paleocene

**Fig. 6.**
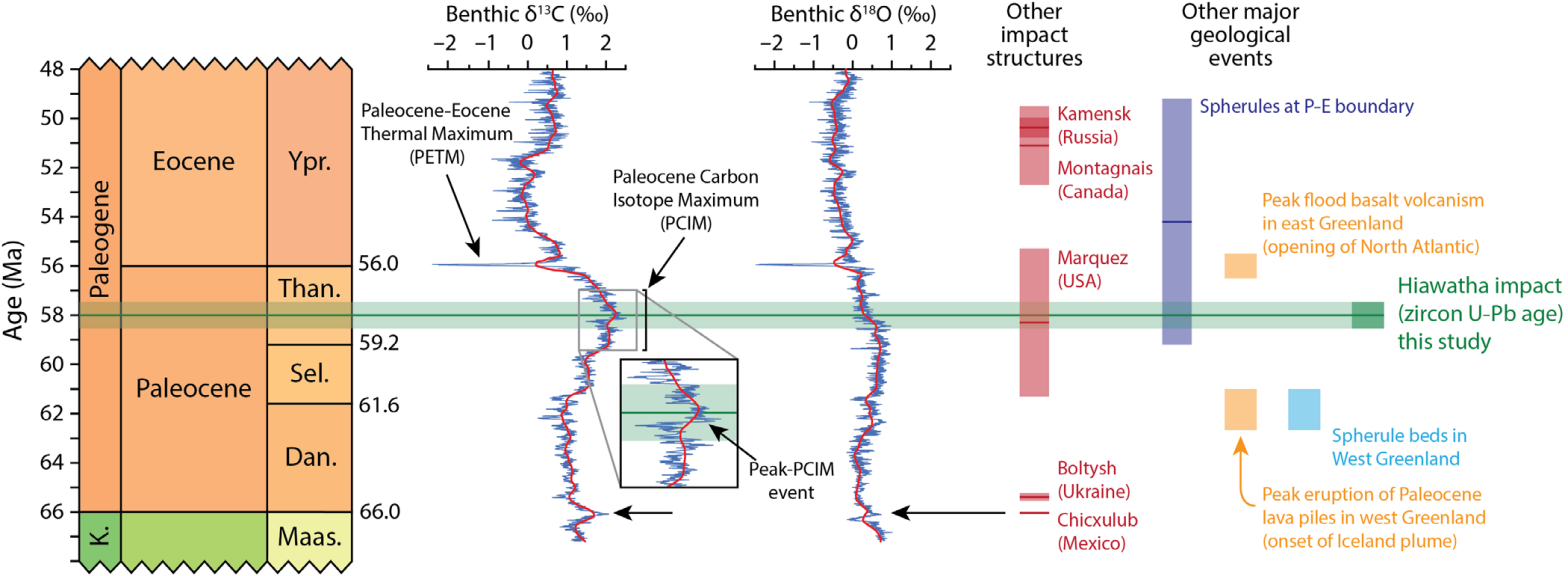
The new age for the Hiawatha impact structure in the context of early Paleogene chronostratigraphy and carbon and oxygen isotope variations. Chronostratigraphic column and absolute ages after (*75*). Carbon and oxygen isotope curves after (*76*) and references therein. Ages for other impact structures with absolute age constraints from compilation of (*42*) and references therein, except the age for Boltysh, which was published very recently (*77*). Age of spherules at P-E boundary from (*50*). Age of peak flood basalt volcanism in east Greenland recalculated from (*33*) and age of peak eruption of Paleocene lava piles in west Greenland from (*34*, *35*). Approximate age of spherule beds in west Greenland is based on their occurrence at low stratigraphic levels of the Paleocene lava pile of western Greenland (*34*). Uncertainties shown at 2σ. K., Cretaceous; Maas., Maastrichtian; Dan., Danian; Sel., Selandian; Than., Thanetian; Ypr., Ypresian.

The new age for the Hiawatha impact structure is close to, but distinguishably older than, the ~55.93-Ma (*32*) Paleocene-Eocene (P-E) boundary (Fig. 6). This boundary is marked by a global carbon isotope excursion and initiation of the Paleocene-Eocene Thermal Maximum (PETM). At ~58 Ma, the Hiawatha impact is also distinguishably older than massive flood basalt volcanism associated with the opening of the northeast Atlantic at ~56 Ma (*33*), and younger than the eruption of the Paleocene lava pile in western Greenland associated with the onset of the Iceland mantle plume at ~62 Ma (*34*, *35*) (Fig. 6), precluding a possible relationship with either of these events. The age of the Hiawatha impact structure overlaps with the current best estimate age for the Marquez impact structure, Texas, USA: 58.3 ± 3.1 Ma (*36*, *37*). It is likely that other impact events occurred around this time. However, craters are quickly removed on Earth [e.g., (*38*)], many preserved impact structures are yet to be found [e.g., (*39*)], and most of the known impact structures have relatively poor age constraints [e.g., (*40*–*42*)].

The Hiawatha impact structure also does not appear to be related to Paleocene-aged spherule beds found in western Greenland and along the northeastern seaboard of the United States. Spherule beds of Paleocene age have been described on Nuussuaq Peninsula in western Greenland [e.g., (*43*, *44*)] and have been proposed as having an impact origin (*45*, *46*). However, it has been shown convincingly that they are of volcanic origin (*43*, *44*, *47*). Further, these beds occur at low stratigraphic levels of the Paleocene lava pile of western Greenland, dated to 62.5 to 61 Ma (*34*,*35*), ruling out a relationship with the Hiawatha structure (Fig. 6). Spherules identified at the P-E boundary along the Atlantic margin of North America (*48*–*50*) have been interpreted as in situ airfall deposits sourced from an impact that occurred close in time to the P-E boundary (*48*). Consequently, they do not appear to be related to the Hiawatha impact structure. If the spherules at the P-E boundary are reworked, their ^40^Ar/^39^Ar age of 54.2 ± 5.0 Ma (*50*) may be consistent with the Hiawatha structure being the source (Fig. 6), but their ex situ setting at the P-E boundary would preclude their use in directly integrating the Hiawatha impact with sedimentary climate proxies. With the exception of grains in the glaciofluvial sediment sample that have been interpreted to be possible ejecta (*1*), no ejecta layer associated with the Hiawatha impact structure has yet been identified.

The timing of the Hiawatha impact does coincide with a broad peak in δ^13^C values in the Late Paleocene known as the Paleocene Carbon Isotope Maximum (PCIM) (Fig. 6). The PCIM appears to have been the result of relatively cool greenhouse conditions between ~63 and ~59 Ma, which facilitated carbon accumulation in reservoirs such as peat bogs, permafrost, and methane hydrates, leading to a positive shift in residual oceanic δ^13^C (*51*, *52*). The large-scale burial of carbon likely also led to the drawdown of atmospheric CO_2_, which agrees with benthic δ^18^O evidence for concurrent global cooling (*53*, *54*). The 57.99 ± 0.54 Ma age of the Hiawatha impact is presently indistinguishable from (i) a ~58-Ma negative step in benthic δ^13^C values, (ii) a subsequent large positive step in benthic δ^13^C values (of ~1‰), known as the peak-PCIM event, and (iii) the initiation of a long-term warming trend when δ^13^C values began a long-term decline, likely reflecting less favorable conditions for sequestration of organic carbon matter in the oceans or on land (Fig. 6) (*53*). However, in the absence of a known ejecta layer, the Hiawatha impact cannot yet be correlated with any of these events. Negative and positive steps in benthic δ^13^C values often reflect sudden shifts in the proportion of carbon removed from the system through carbonate or organic matter burial, reflecting contractions and expansions, respectively, of sedimentary reservoirs such as peat bogs, permafrost, and marine organic matter (*53*). However, changes in organic burial fluxes appear to occur too slowly to adequately explain very sharp steps in δ^13^C, such as the positive excursion of the peak-PCIM event (*53*). The Chicxulub impact appears to have been particularly deadly and disruptive to marine productivity due to both its large size and the composition of the target rocks such that enormous amounts of climate-forcing species, including CO_2_ and S, were released into the atmosphere (*55*–*57*). Although the Chicxulub impact event is the only known impact conclusively correlated with a major mass extinction event (*58*), it has been proposed that smaller impacts can have detrimental ecological effects and induce extinction events (*59*–*61*). Consequently, the possibility remains that the relatively large Hiawatha impact event may be recorded in the Late Paleocene δ^13^C record and that it affected local or global productivity. A full understanding of the precise timing and possible effect of the Hiawatha impact upon the global climate of the Late Paleocene may only come from future identification of associated ejecta, which would enable direct integration of the impact event with sedimentary climate proxies.

## MATERIALS AND METHODS

### ^40^Ar/^39^Ar analysis

Fifty grains (1 to 3 mm in diameter; fig. S3) from the sample of well-sorted, fine sand (HW21-2016) were selected for ^40^Ar/^39^Ar analysis using a binocular microscope. The grains were cleaned in distilled water followed by acetone and then, along with crystals of the neutron fluence monitor Fish Canyon sanidine [FCs; 28.201 Ma; (*62*)], loaded into single wells of 21-well, 18-mm-diameter aluminum sample discs. The sample discs were then stacked, wrapped in aluminum foil, and encapsulated in a heat-sealed quartz tube. Fast neutron irradiation was carried out in the CLICIT (Cadmium-Lined In-Core Irradiation Tube) facility at the General Atomics TRIGA (Training, Research, Isotopes, General Atomics) reactor at Oregon State University for a total duration of 16 hours between 24th and 26th of July 2019. The single-grain incremental heating experiments were performed in the noble gas isotope laboratory at the Natural History Museum of Denmark following previously documented instrumentation and procedures (*63*, *64*), which are summarized here. Step-heating of the grains was achieved by the progressive ramping up of the power of a defocused 50-W Synrad CO_2_ laser beam in 0.5- to >1-W increments beginning at 1 to 1.5 W and until fusion was achieved, which typically occurred between 4 and 15 W, depending on the size of the grain. The initial low laser power (<2 W) degassing steps yielded no visible glowing of the grain and commonly produced small amounts of radiogenic ^40^Ar (^40^Ar*) with model ages as young as 10 Ma. This is attributed to degassing of low-temperature alteration phases. The sample gas cleanup was through an all-metal extraction line, equipped with a cold trap operating at −130°C, to remove H_2_O, and two water-cooled SAES GP-50 getter pumps to remove reactive gases. Analyses of unknowns, blanks, and monitor minerals were carried out in identical fashion on a Nu Instruments multicollector Noblesse noble gas mass spectrometer, during a fixed period of 600 s in 11 data acquisition cycles, in which ^40^Ar and ^39^Ar were measured on the high-mass Faraday detector (F), ^38^Ar and ^37^Ar on the axial ion counter, and ^36^Ar on the low-mass ion counter, with baselines measured every cycle. Measurement of ^39^Ar was followed by the simultaneous measurement of the ^40^Ar, ^38^Ar, and ^36^Ar ion beams, followed by measurement of ^37^Ar. Ion beam switching was achieved by varying the field of the mass spectrometer magnet and with minor adjustment of the quad lenses. Data collection and reduction were carried out using the program “Mass Spec” (by A. Deino, Berkeley Geochronology Center). Every second heating step on an impactite grain was followed by a blank analysis, with typical values being <2.0 × 10^−16^ mol ^40^Ar and 6 × 10^−18^ mol ^36^Ar. Correction factors that combine mass fractionation and detector efficiencies (detector intercalibration) were derived from a time series of measured (instrumental) atmospheric argon aliquots, delivered from a calibrated air pipette as previously formulated (*63*). Air pipettes were run before, during, and after the analysis of the unknowns and FCs so that the latter were fully bracketed. Decay and other constants used in the ^40^Ar/^39^Ar age calculations, including correction factors for interference isotopes produced by nucleogenic reactions, are given in the caption to data S1.

### Zircon sample preparation

Impact melt rock samples HW19-01 and HW19-05 were disaggregated in a jaw crusher, and ring-and-puck–style mill before their heavy mineral fractions were concentrated by magnetic separation (with a hand magnet and Frantz magnetic separator) and heavy liquid density separation (using methylene iodide with a density of approximately 3.3 g/cm^3^). Under the binocular microscope, two types of zircon grains were picked from the heavy mineral separates of each sample: (i) Transparent, apparently undeformed grains were picked with the aim to date the target rocks, and (ii) grains with a turbid, or cloudy, appearance—which are more likely to be recrystallized and contain age-reset domains [e.g., (*65*)]—were picked with the aim to date the impact event. The grains were placed on double-sided sticky tape and cast in 2.5-cm-diameter epoxy mounts. The mounts were polished with a diamond suspension to expose grain interiors, and a final polish with colloidal silica prepared the grains for microstructural analysis. The exposed cross sections of the grains were imaged in CL, BSE, and secondary electron (SE) modes on an FEI Quanta FEG 650 SEM at the Swedish Museum of Natural History, Stockholm, Sweden, which is equipped with a Gatan ChromaCL2 system. Ten grains of interest were selected for microstructural characterization by EBSD analysis on the same SEM. After EBSD analysis, the mount was cleaned, coated in Au, and analyzed for U-Pb isotopic composition and age by SIMS on the CAMECA IMS1280 ion microprobe at the NordSIMS Laboratory, Swedish Museum of Natural History.

### EBSD analysis

EBSD analysis was performed with an Oxford Instruments Nordlys detector. Grains were indexed for zircon, reidite, and monoclinic ZrO_2_ using match units based on crystallographic data from (*66*–*68*). Analytical conditions and parameters include the following: accelerating voltage of 20 kV, working distance of ~18 mm, stage tilt of 70°, electron backscatter pattern (EBSP) binning of 4 × 4, EBSP gain set to “High,” background defined with collection of 128 frames, Hough resolution set to 60, and band detection minimum/maximum of 6/8. Maps were collected with step sizes between 75 and 150 nm. Data collection was performed in Oxford Instruments AZtec software, and processing was done in Oxford Instruments Channel 5 software v.5.12. Data cleaning in Channel 5 comprised a wildspike correction (applied to all grains) and a nearest-neighbor zero solution extrapolation, based on six (applied to HW19-01 z039 and z101), seven (applied to HW19-01 z009, z012, z016, z019, z020, and z047 and HW19-05 z104), or eight (applied to HW19-01 z010) neighbors.

### Uranium-lead dating

The zircon grains were analyzed for U-Pb isotopic composition and date by SIMS in two analytical sessions. For the U-Pb analyses, an Oregon Physics Hyperion H201 RF plasma high-brightness oxygen source was used to generate O_2_^−^ ions and the primary column tuned in critical focusing mode with a small (5 μm by 5 μm) raster applied to produce an analysis pit ~10 μm across. To limit sampling of extraneous material by small beam aberrations in critical focusing mode, the field of view on the sample was further restricted by closing the field aperture to limit analyzed ions to those from a central, approximately 6 μm by 6 μm square area. Routine U-Pb analyses of zircon are performed at a mass resolution (*M*/Δ*M*) of approximately 5000 [e.g., (*69*)], which is sufficient to separate Pb isotope peaks from nearby HfSi^+^ species, but here, the analyses were run with a mass resolution of approximately 8000 to negate any possible interferences related to incorporation of extraneous elements in the granular zircon grains. In addition, rare occurrences of baddeleyite in the recrystallized zircon grains were avoided to negate any possible issues associated with the zircon standardization. Analyses were performed using a peak switching routine, with a single ion-counting electron multiplier as the detection device. An energy window of 45 eV was used throughout, with energy adjustments made using the ^90^Zr_2_^16^O peak. Precise mass calibration was maintained by using an automatic routine in the CAMECA CIPS software to scan over large peaks and extrapolate the mass to B-field curve for peaks between these reference points (e.g., Pb isotopes were calibrated by centering the ^94^Zr_2_^16^O peak at nominal mass 204 and the ^177^Hf^16^O^2^ peak at nominal mass 209). In both analytical sessions, 91500 standard zircon [isotope dilution thermal ionization mass spectrometry (ID-TIMS) ^206^Pb/^238^U age = 1062.4 ± 0.8 Ma; (*70*)] was used as the calibration reference material. Temora 2 [ID-TIMS ^206^Pb/^238^U age = 416.78 ± 0.33 Ma; (*71*)] was analyzed as a secondary reference material, returning a weighted mean ^206^Pb/^238^U age of 419.5 ± 4.3 Ma (MSWD = 0.17, probability = 0.97, *n* = 6) for session #1 and 421.3 ± 5.1 Ma (MSWD = 0.28, probability = 0.93, *n* = 6) for session #2. We used the decay constant values of (*72*), and all uncertainties are presented at the 2σ level unless otherwise stated. Isotopic ratios and dates for zircon grains from Hiawatha and standard zircon Temora 2 are reported in data S2 and S3. A final consideration in U-Pb dating of impact-recrystallized zircon grains is their relatively high concentrations of nonradiogenic (i.e., common) Pb (Pb_c_). As is routine in zircon U-Pb geochronology, we applied a Pb_c_ correction to the U-Pb data. We estimated the amount of ^206^Pb in the analyses that is not radiogenic by measuring the abundance of nonradiogenic ^204^Pb. Between 1.6 and 9.2% of ^206^Pb in the nine analyses used to calculate the age of the Hiawatha impact structure is Pb_c_, compared to a maximum of 0.05% in the ca. 1915-Ma, unaltered grains (data S2). As is routine in studies of impact-recrystallized zircon (*65*), we assumed that Pb_c_ was largely introduced into crevices in the granular grains during the polishing stage of grain mount preparation and thus that Pb_c_ is modern (i.e., 0 Ma in age) and has a modern-day Stacey and Kramers (1975) (*73*) Pb composition. If some of Pb_c_ is actually ancient, we will have slightly underestimated the age of the impact. However, this effect is negligible; in a worst-case scenario, in which all Pb_c_ encountered in the granular zircon from Hiawatha dates to ca. 1915 Ma (the crystallization age of most grains in these samples), the weighted mean ^206^Pb/^238^U age becomes just 0.5 Ma older, i.e., indistinguishable from the age obtained when assuming Pb_c_ to be modern. To calculate a weighted mean ^206^Pb/^238^U age for the impact structure, we calculated a weighted mean ^206^Pb/^238^U age for the youngest two analyses and then did the same for the youngest three, four, five, etc., analyses. The youngest nine analyses are statistically indistinguishable, but including the 10th youngest analysis in weighted mean calculations results in poor statistics [probabilities below the 0.05 to 0.30 thresholds recommended by (*74*)], indicating excess scatter as a result of incomplete impact-related Pb loss.

## Supplementary Materials

### This PDF file includes:

Supplementary Text Figs. S1 to S13 References
