# Supplementary material for: A Late Paleocene age for Greenland’s Hiawatha impact structure
Source: Sci Adv. 2022 Mar 9;8(10):eabm2434. doi: 10.1126/sciadv.abm2434 (PMC8906741; doi:10.1126/sciadv.abm2434)
Supplement: Supplementary file 1 — Supplementary Text Figs. S1 to S13 References [file sciadv.abm2434_sm.pdf]

Science Advances
AAAS

Supplementary Materials for

A Late Paleocene age for Greenland's Hiawatha impact structure

Gavin G. Kenny*, William R. Hyde, Michael Storey*, Adam A. Garde, Martin J. Whitehouse, Pierre Beck, Leif Johansson, Anne Sofie Søndergaard, Anders A. Bjørk, Joseph A. MacGregor, Shfaqat A. Khan, Jérémie Mouginot, Brandon C. Johnson, Elizabeth A. Silber, Daniel K. P. Wielandt, Kurt H. Kjær, Nicolaj K. Larsen*

*Corresponding author. Email: gkennyeire@gmail.com (G.G.K.); michael.storey@snm.ku.dk (M.S.); nicl@sund.ku.dk (N.K.L.)

Published 9 March 2022, *Sci. Adv.* **8**, eabm2434 (2022)
DOI: 10.1126/sciadv.abm2434

The PDF file includes:

Supplementary Text
Figs. S1 to S13
Legends for data S1 to S3
References

Other Supplementary Material for this manuscript includes the following:

Data S1 to S3

Supplementary text

Descriptions of grains from glaciofluvial sand sample HW21-2016 that were selected for $^{40}Ar/^{39}Ar$ analysis. As the grains were too small to be mounted in thin section prior to $^{40}Ar/^{39}Ar$ analysis, only a brief description of the grain exterior was possible. Images of all analyzed grains are given alongside the $^{40}Ar/^{39}Ar$ spectra in Fig. S3.

QL01 Chemical sedimentary grain with presumed quartz ooids – note pale, almost perfectly shaped, ellipsoidal to spherical bodies of silica with nuclei of ?quartz fragments.

QL03 Melt grain with imperfectly shaped microspherulites of zoned feldspar in a dark brown glassy matrix with organic carbon, and pale fragments of quartz and (presumably) partially melted feldspar

QL04 Felsic melt grain with closely packed, yellowish microspherulites with concentric structure. Perlitic fracturing seems to be present.

QL05 Dark brown melt grain with apparent hemicrystalline (feldspathic?) melt phase and numerous small mineral fragments.

QL07 Heterogeneous melt grain with dark, possibly organic carbon-rich patches in a light grey matrix and several mineral fragments.

QL08 Heterogeneous melt grain with zoned microspherulites formed around cores of quartz fragments, an indistinct zone of flattening or flow, irregular fragments of dark grey material possibly with organic carbon, and fragments of quartz.

QL09 Melt grain with dark, heterogeneous matrix, pale schlieren (feldspar?) and common fragments of quartz.

QL10 Grey melt grain with scattered, pale yellow, poorly developed spherulites and mineral fragments of mainly quartz.

QL11 Melt grain with a dark brown, aphanitic matrix, a few indistinct microspherulites, pale yellow patches of presumed partially dissolved feldspar and fragments of quartz.

QL12 Melt grain with numerous, closely packed microspherulites with radiating marginal parts in a greenish grey matrix, dark, irregular aphanitic patches and fragments of quartz and feldspar.

QL14 Melt grain with dark, ?organic carbon-rich glassy matrix, a few small oblong, pale spherulites, yellow patches of ?partially dissolved feldspar and fragments of quartz.

QL15 Heterogeneous pale grain with light grey presumed melt matrix and abundant clasts.

QL16 Black, apparently microporphyritic melt grain with microlites of presumed pyroxene and ilmenite in a hemicrystalline feldspathic matrix with organic carbon and fragments of quartz.

QL17 Black ?microporphyritic, apparently mafic melt grain with microlites of presumed pyroxene and ilmenite in a black matrix with presumed organic carbon and fragments of quartz.

QL18 Pale, heterogeneous melt grain with presumed felsic to intermediate matrix composition and numerous mineral fragments. Feldspathic microspherulites may be present.

QL19 Melt grain dominated by irregular, light grey ?feldspar microspherulites and mineral fragments in a darker matrix with small black spots.

QL20 Yellow melt grain with indistinct but apparently abundant, irregularly shaped feldspar microspherulites impinging on each other, and dark patches with sulphide mineral specks.

QL21 Melt grain dominated by a greenish grey, aphanitic component and a heterogeneous, darker component presumably rich in organic carbon, and fragments of quartz up to 1 mm across.

QL22 Pale grey–yellow melt grain with schlieric microstructure and fragments of quartz.

QL23 Heterogeneous, patchy presumed melt grain with yellowish and dark components. Possibly microspherulites in the yellowish component, which may be feldspar.

QL24 Melt grain dominated by a dark greenish grey, aphanitic (?glassy) matrix and an almost black fragment 0.3 mm across.

QL25 Heterogeneous, patchy, dark grey to black grain without distinct features.

QL26 Heterogeneous, patchy, dark grey to black grain with elongate, light grey patches up to 0.1 mm across.

QL27 Heterogeneous, grey to dark grey, very fine-grained grain with an indistinct sub-planar microfabric. Apparent mineral clasts seem to be partially dissolved.

QL28 Fragmental grain with numerous mineral fragments and dark, rounded ?melt particles that may be rich in organic material.

QL29 Heterogeneous melt grain with microspherulites of zoned feldspar up to 0.2 mm across in a greenish grey matrix, and fragments of quartz and feldspar.

QL30 Melt grain with dark, microcrystalline matrix which may be rich in organic carbon, pale yellow patches of partially dissolved fragments of ?feldspar and common fragments of quartz.

QL31 Melt grain with greenish grey matrix and numerous different mineral fragments including mesoperthite feldspar.

QL32 Heterogeneous melt grain with a greenish grey matrix, irregular dark inclusions up to almost 1 mm across and a rounded patch with high reflectance, probably rich in iron oxide or ilmenite.

QL33 Heterogeneous, greenish grey grain with abundant clasts displaying epitaxial growth of white material, presumably feldspar. Several irregular voids with interior black lining of walls (?organic carbon).

QL34 Heterogeneous melt grain with closely packed, mostly yellowish ?feldspar microspherulites and fragments of quartz. In some parts of the grain both microspherulites and interstitial matrix are dark, probably due to dispersed organic carbon.

QL35 Almost black melt grain with scattered microspherulites about 0.05 mm across and mineral fragments.

QL36 Dark, heterogeneous melt grain with schlieric microstructure and angular mineral fragments.

QL37 Heterogeneous melt grain with epitaxial growth of pale-yellow feldspar on dark clasts.

QL38 Greyish yellow melt grain with indistinct, apparently closely packed ?feldspar microspherulites and mineral fragments.

QL39 Heterogeneous, predominantly dark brown melt grain with microspherulites, pale grey patches of presumed partially dissolved feldspar clasts and other mineral clasts. Organic carbon is likely to be present.

QL40 Grey melt grain with schlieric microstructure and several fragments of quartz.

QL41 Heterogeneous, greenish grey melt grain with schlieric microstructure, partially melted material and fragments of quartz.

QL42 Dark brown to black melt grain with microporphyritic structure including numerous pale, lath-shaped feldspar microlites. Fragments of quartz are also visible.

QL43 Dark brown melt grain with pale areas dominated by microspherulites and epitaxial microlites as well as numerous, very small mineral fragments. The dark matrix color is likely to be caused by a high content of organic carbon.

QL44 Greenish grey melt grain with indistinct, lath-like microlites, probably of feldspar.

QL45 Dark brown melt grain with granular appearance to matrix and small mineral clasts less than 0.1 mm across.

QL46 Light brown melt grain with apparently hemicrystalline matrix. No mineral fragments are visible.

QL47 Two-component melt grain with black and grey components. The latter contains cored microspherulites, interpreted as consisting of feldspar.

QL48 Dark greenish grey melt grain with patchy microstructure and local cored microspherulites.

QL49 Brown melt grain with a granular appearance of matrix and fragments of quartz less than 0.1 mm across.

QL50 Heterogeneous melt grain with dark, almost black and yellow components. No obvious mineral fragments are present.

QL52 Melt grain with grey, cored microspherulites and a dark brown, probably hemicrystalline matrix.

QL53 Relatively homogeneous melt grain with apparent pale, indistinct microspherulites and a darker matrix.

QL54 Melt grain consisting of mineral fragments rimmed by grey layers of fibrous, epitaxial material (?feldspar) and a subordinate, dark brown to black matrix.

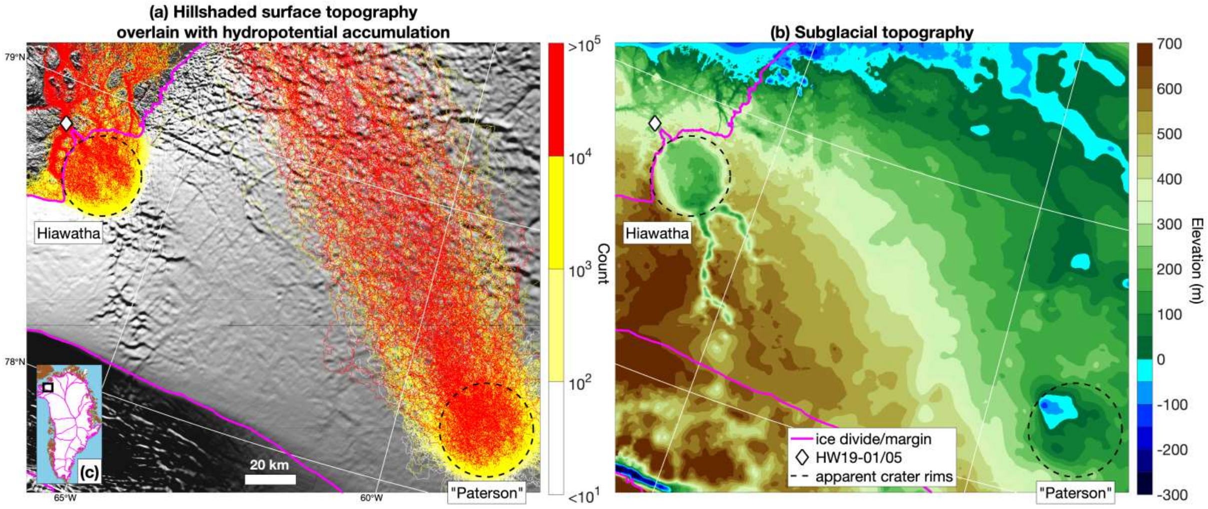

Fig. S1. Modeled subglacial water routing in northwestern Greenland. (**A**) Hillshaded surface topography from ArcticDEM *(78)* overlain with modeled subglacial water accumulation. The latter field is calculated using the Shreve hydropotential *(79)* and 100 realizations of BedMachine v3 bed topography with an additional normally distributed elevation uncertainty whose standard deviation is 200 m. These water pathways are then summed into the total count shown. In this model, subglacial water is permitted to flow everywhere (a conservative scenario, given that the bed is likely frozen in some regions; *(30)*), but that water is only produced uniformly inside of the apparent crater rims. In this manner, the effect of subglacial and subaerial transport from the two structures can be isolated from other possible confounding factors. (**B**) BedMachine v3 bed topography *(23)*. Assuming that samples HW19-01 and -05 were recently transported subglacially, prior to their proglacial discharge and subaerial recovery, this analysis demonstrates that they most likely originated from the Hiawatha impact structure, and the second possible impact structure ("Paterson"; *(25)*) is unlikely to have recently contributed significant material to the sampled floodplain. Any subglacially entrained samples from that second structure are consistently routed farther north, toward the floating terminus of Humboldt Glacier. Further, samples HW19-01 and -05 are found at the intersection of two of the primary modeled transport routes for material from beneath Hiawatha Glacier.

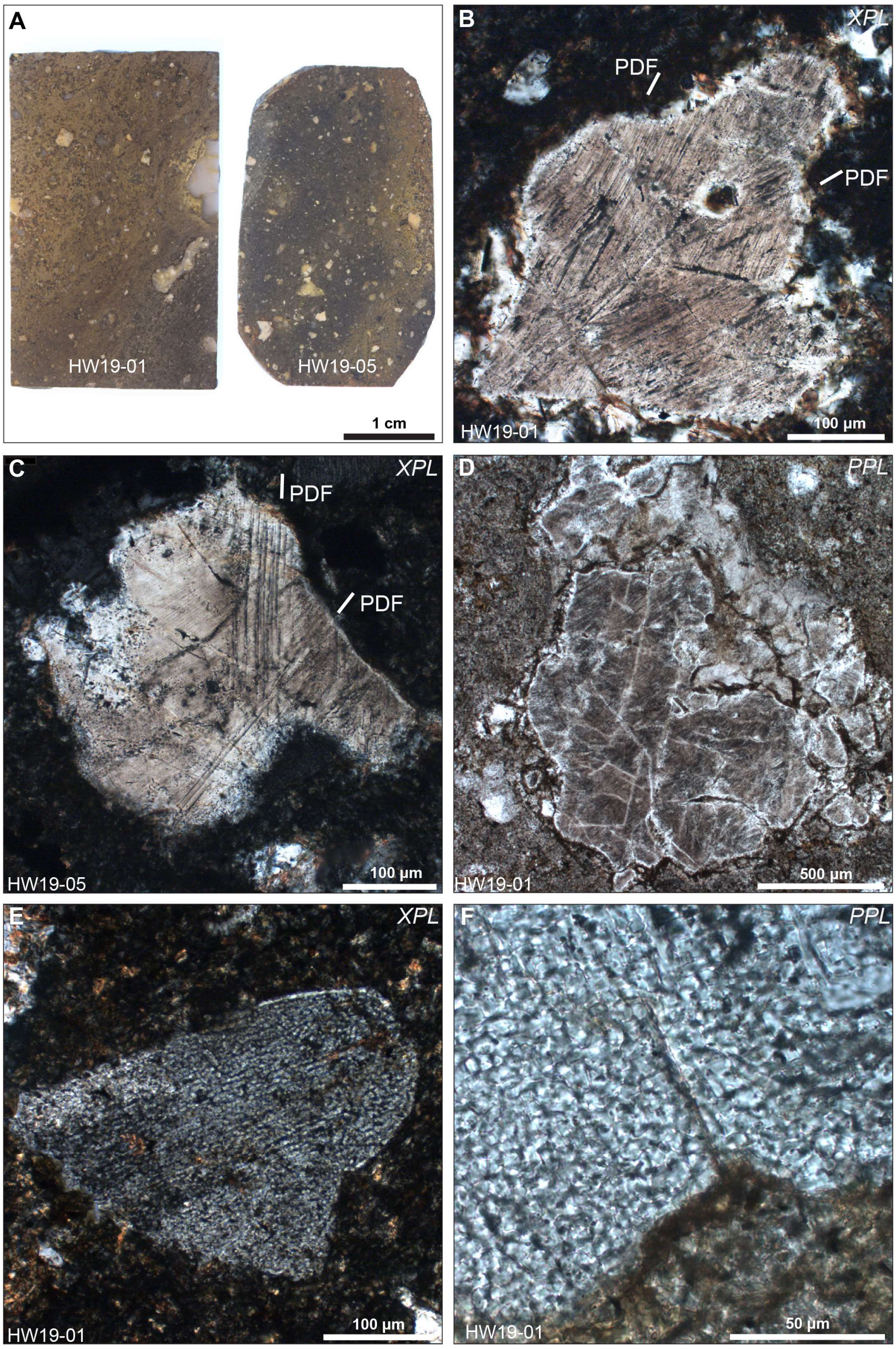

Fig. S2. Photographs of impact melt rocks HW19-01 and HW19-05 (A) and photomicrographs of shocked silicate clasts in the impact melt rocks (B–F). (**A**) Photographs of impact melt rock hand samples from which zircon was separated. (**B**) Lightly toasted quartz clast with two sets of planar deformation features (PDFs). (**C**) Lightly toasted quartz showing partial digestion, seen as embayments with infilling of orthoclase glass. (**D**) Highly toasted quartz grain in feldspathic melt rock matrix. (**E–F**) Shocked feldspar showing checkerboard texture in feldspathic melt rock matrix. PPL – plane polarized light; XPL – cross-polarized light.

A

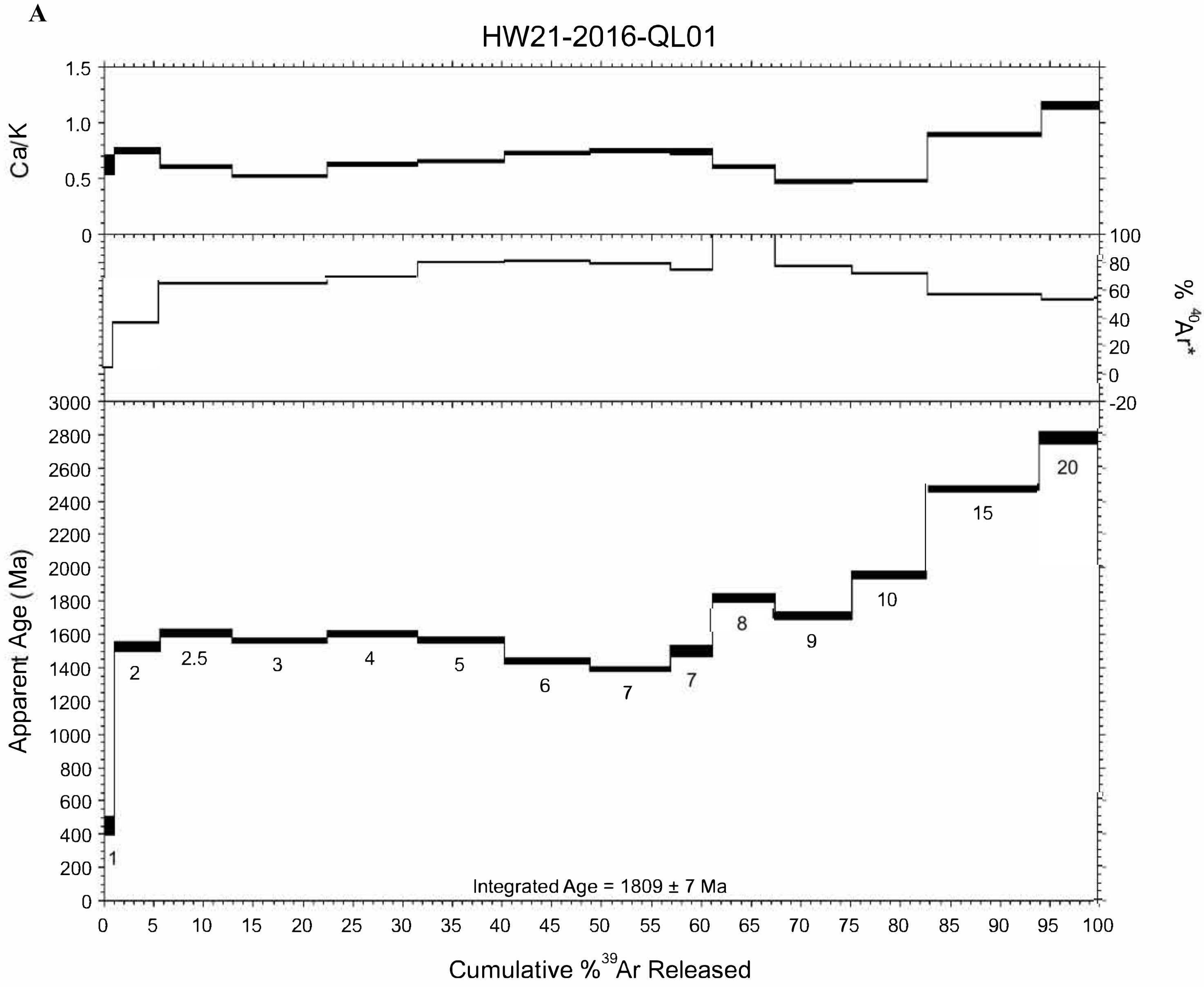

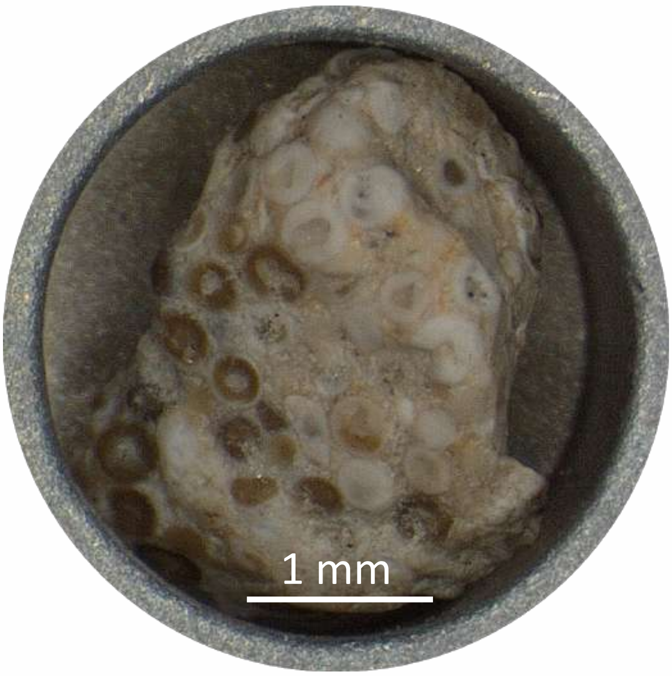

Fig. S3. Age spectra and images of impactite grains that underwent $^{40}Ar/^{39}Ar$ analysis. The number below each heating step is the laser power (watts). The integrated, or total gas age is equivalent to a K/Ar age and is calculated by weighting the individual step ages according to the % of ^{39}Ar released. MSWD – mean square of weighted deviates. P – probability.

B

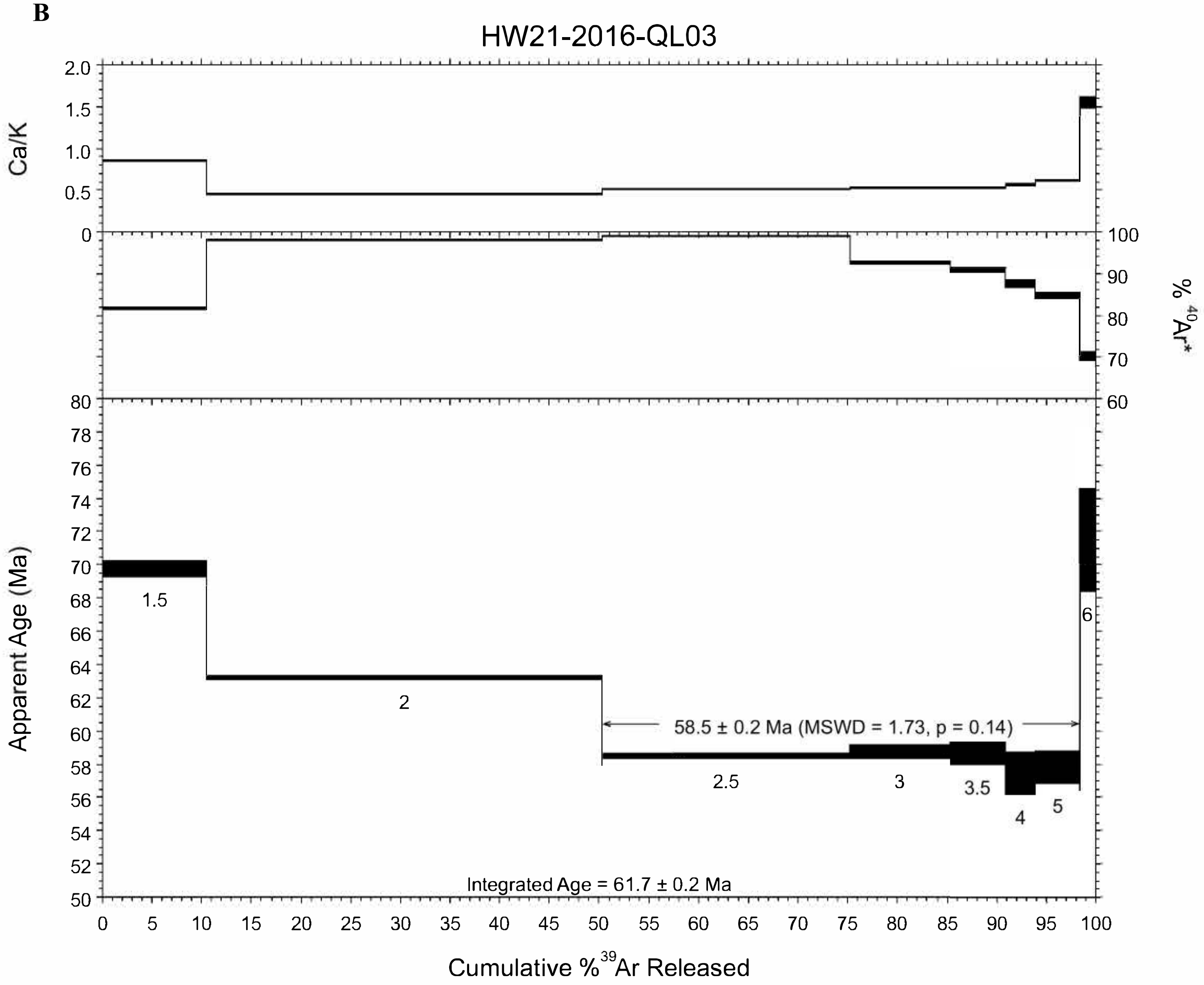

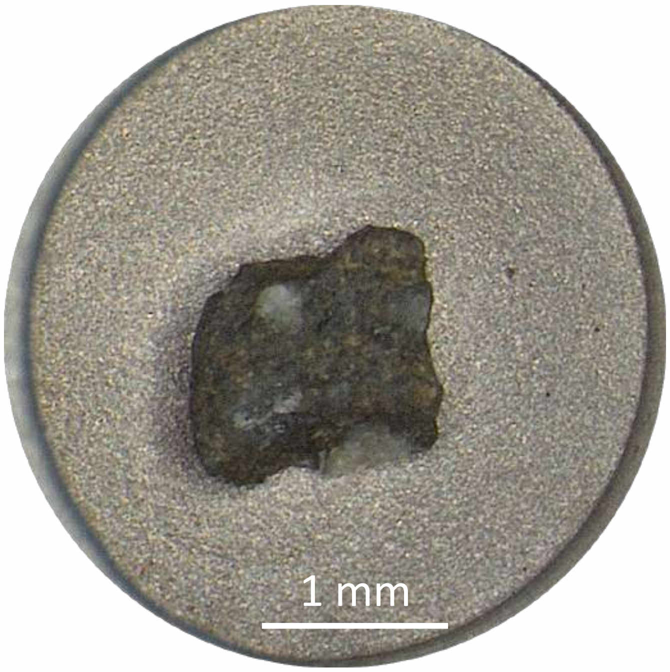

Fig. S3 continued.

C

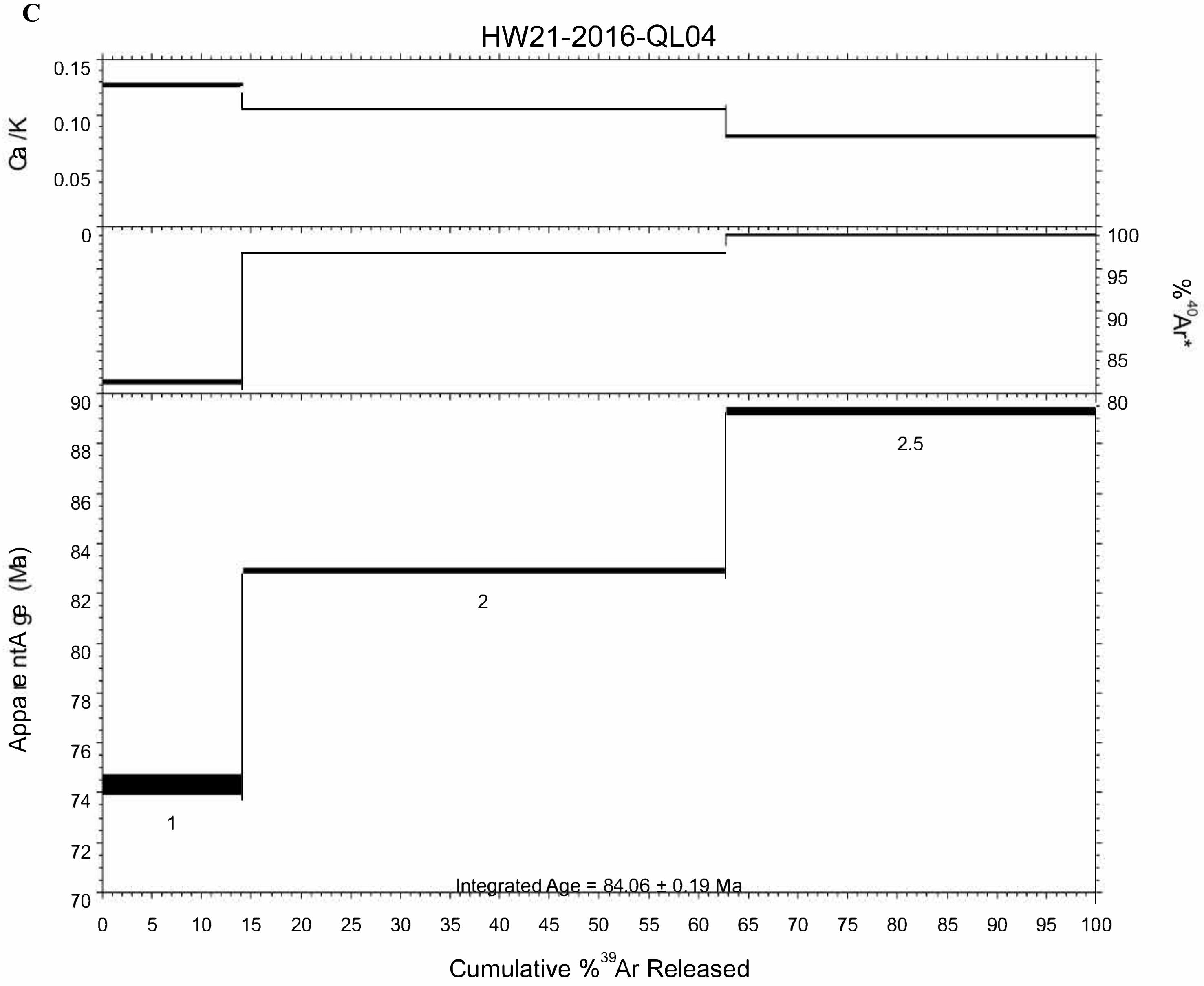

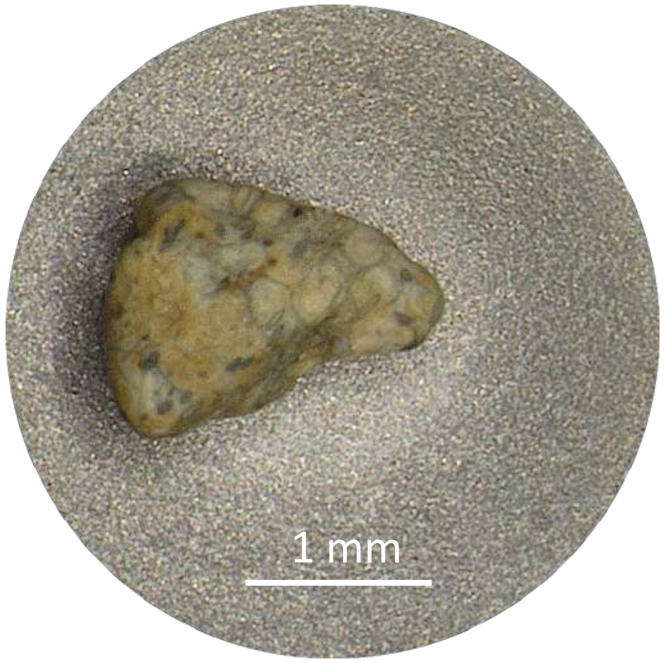

Fig. S3 continued.

D

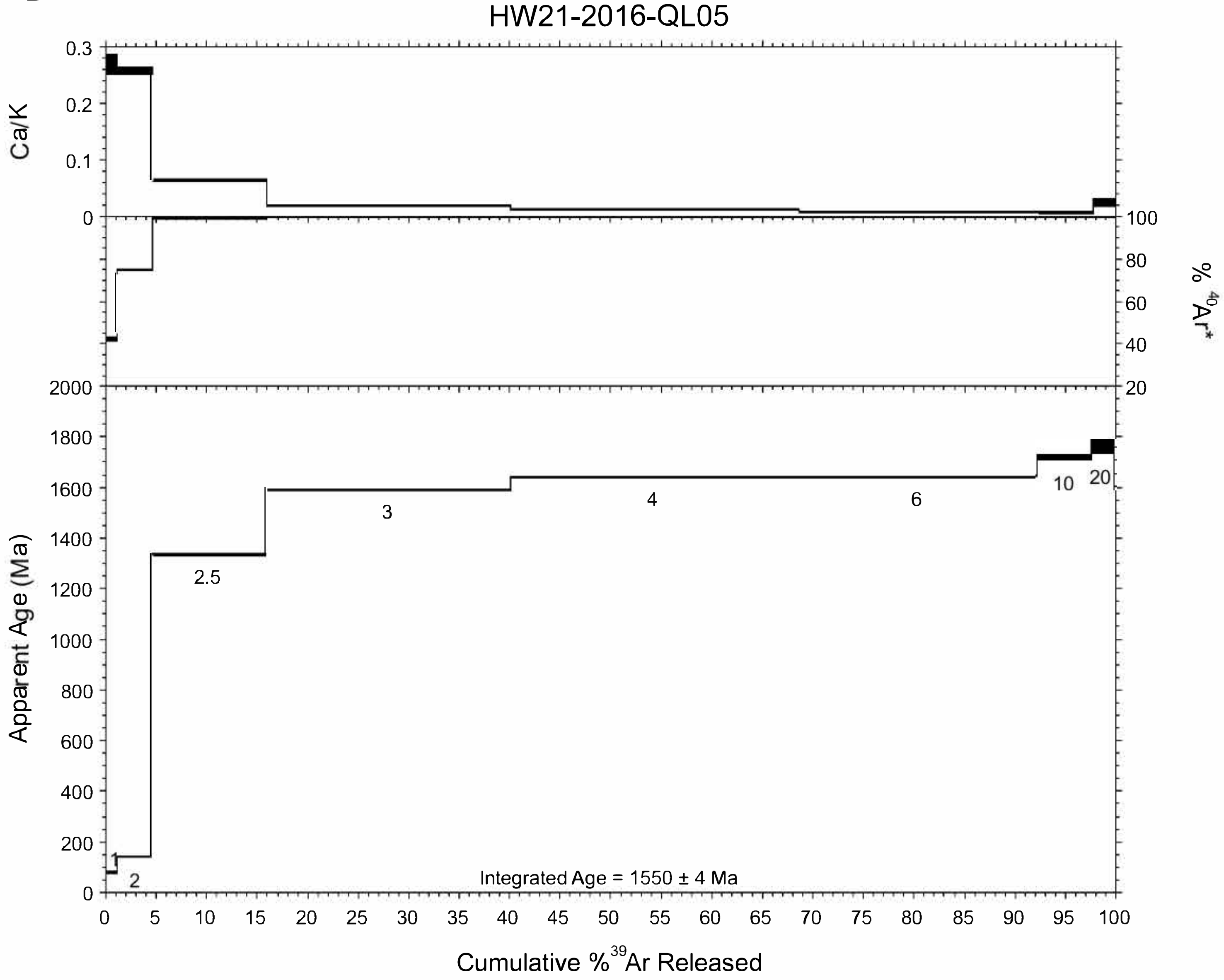

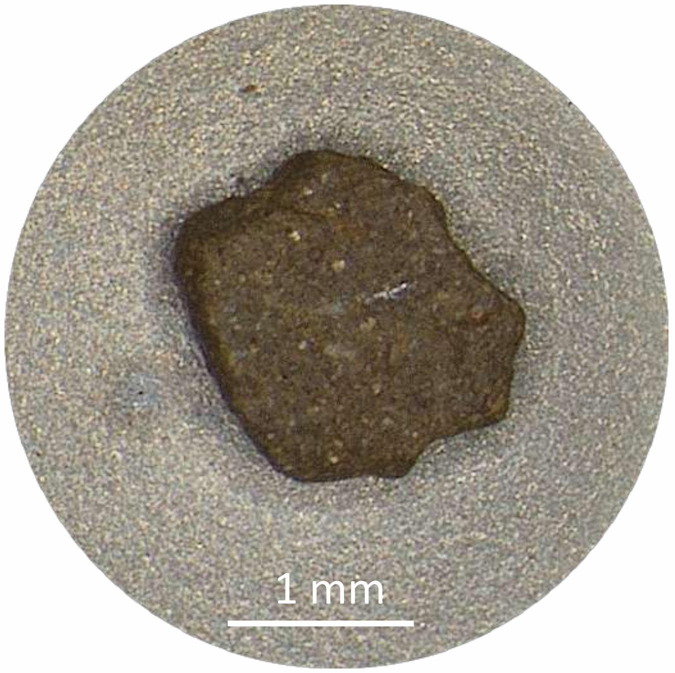

Fig. S3 continued.

E

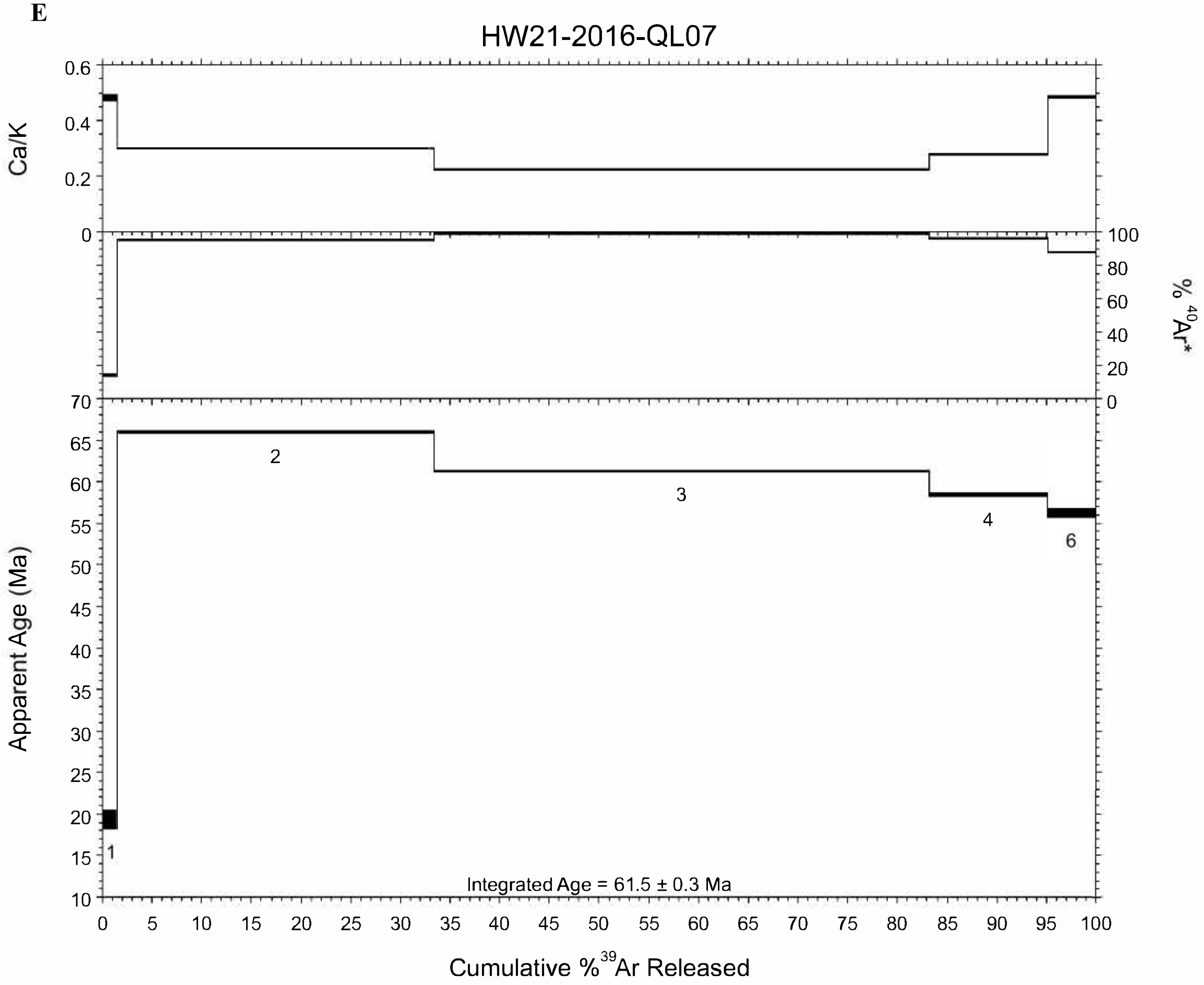

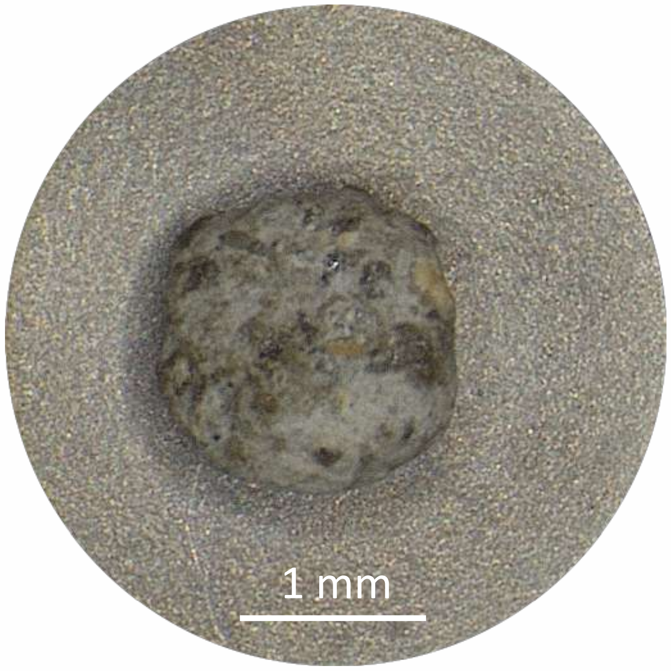

Fig. S3 continued.

F

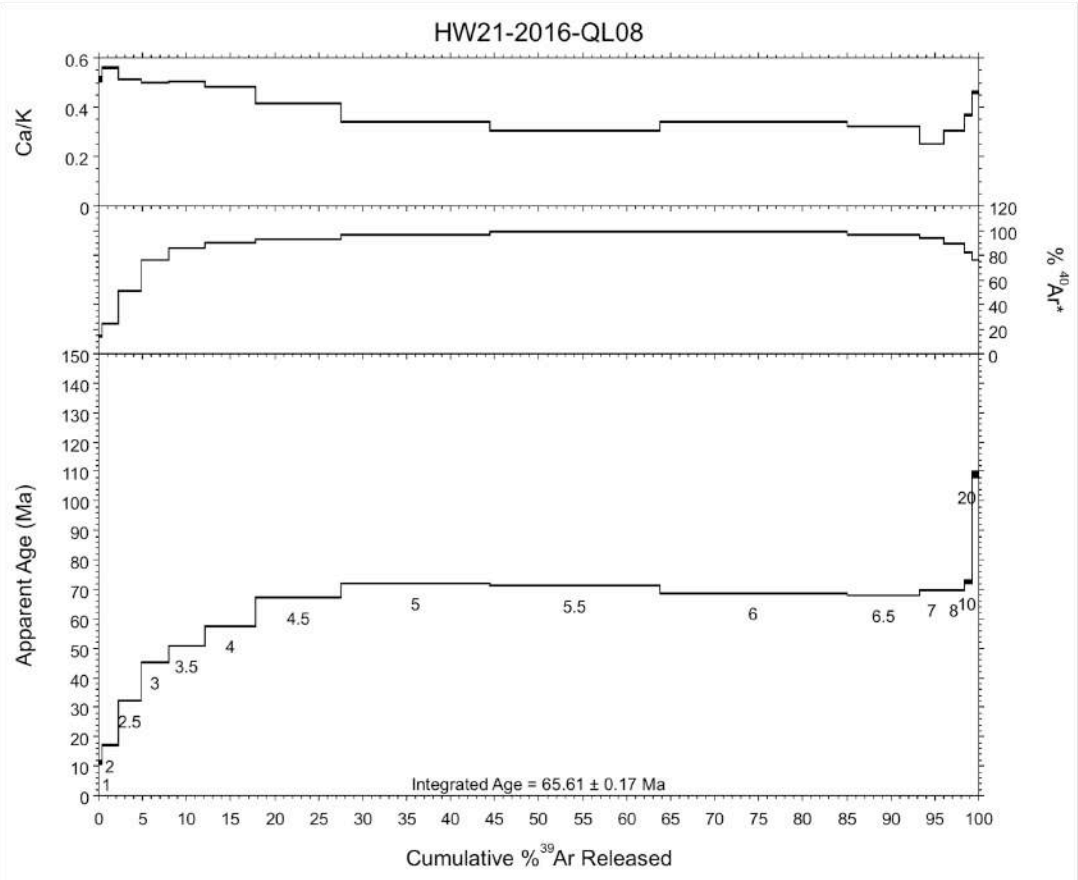

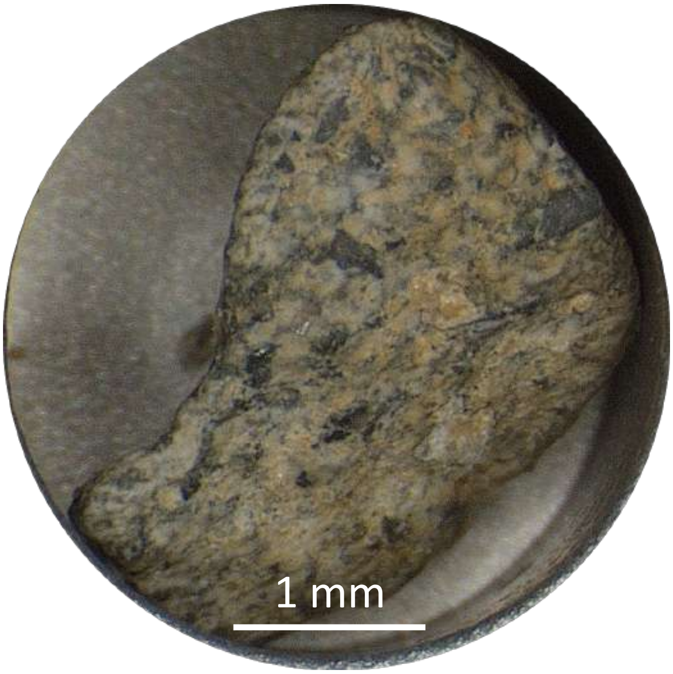

Fig. S3 continued.

G

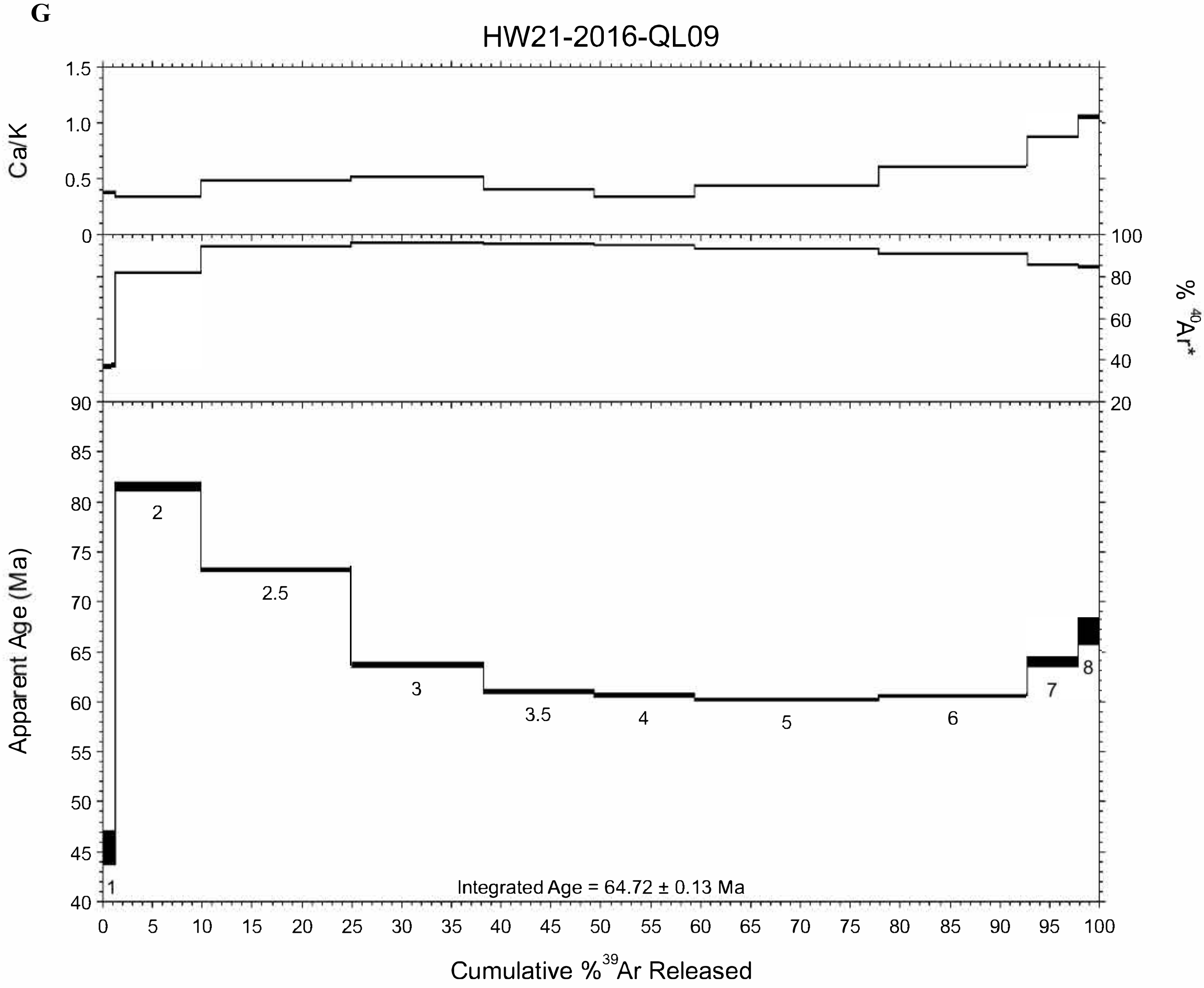

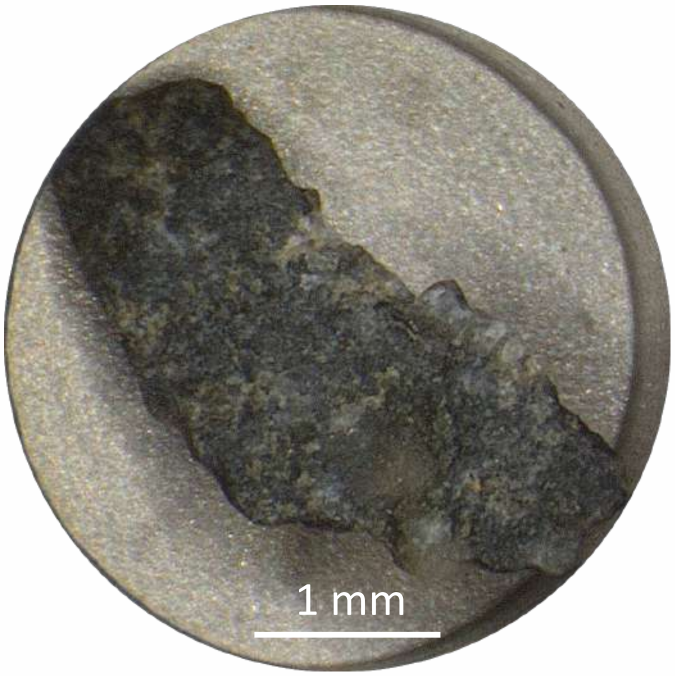

Fig. S3 continued.

H

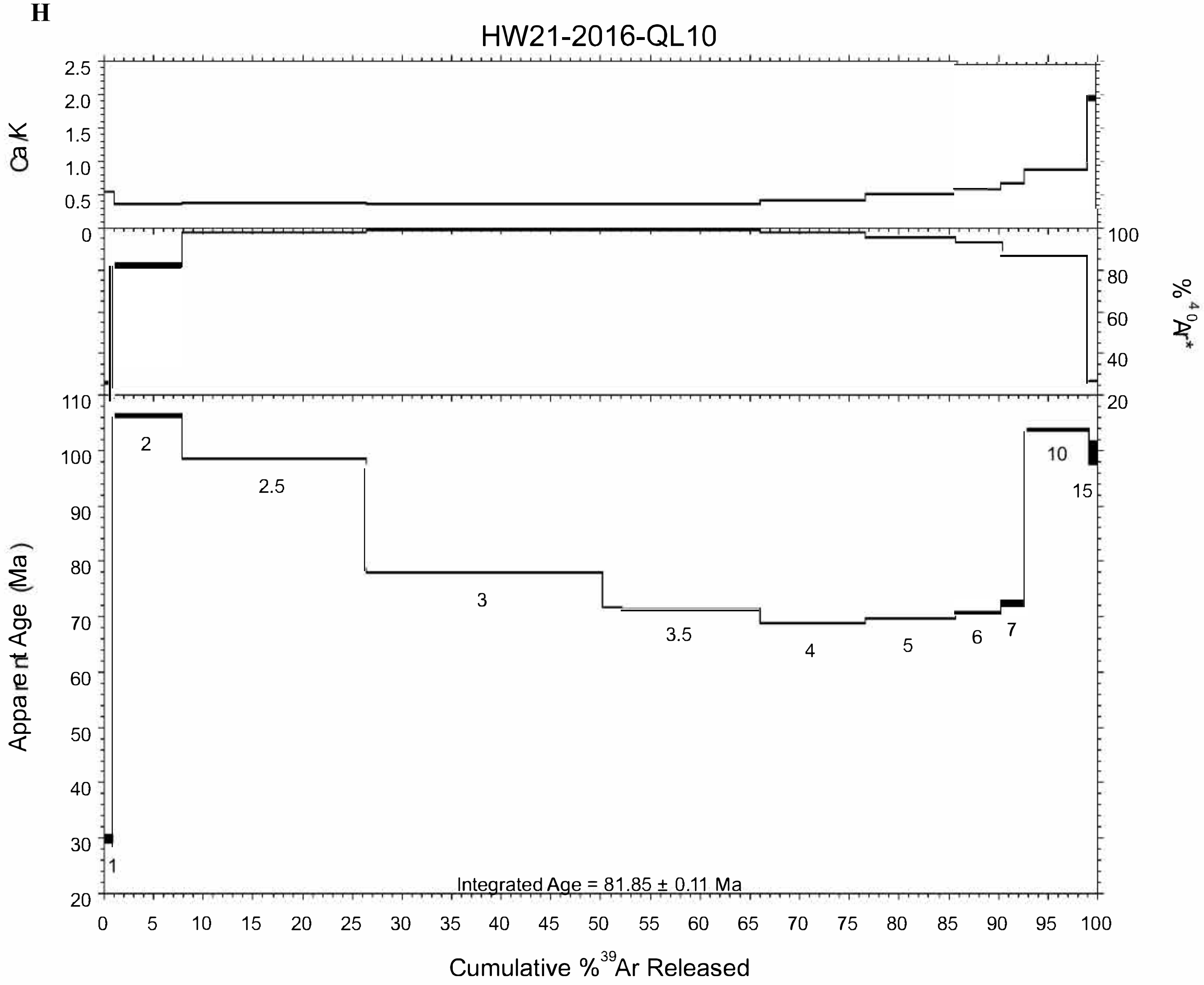

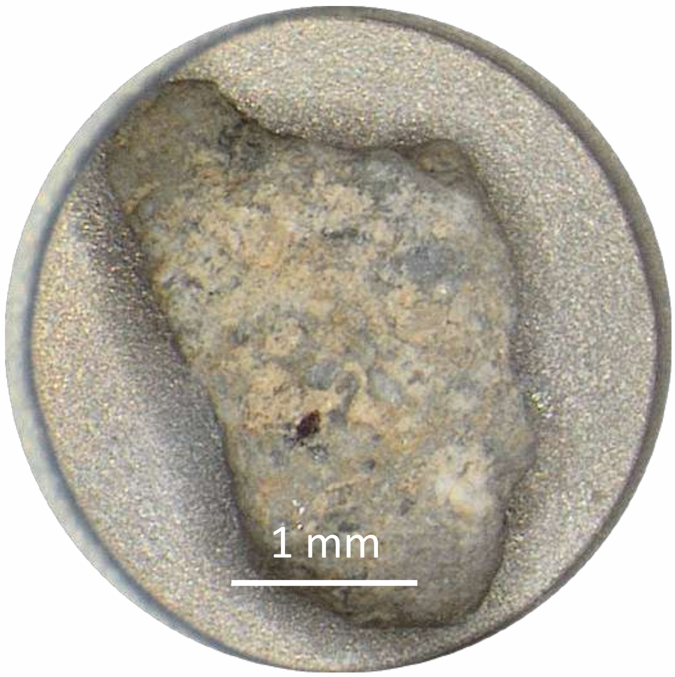

Fig. S3 continued.

I

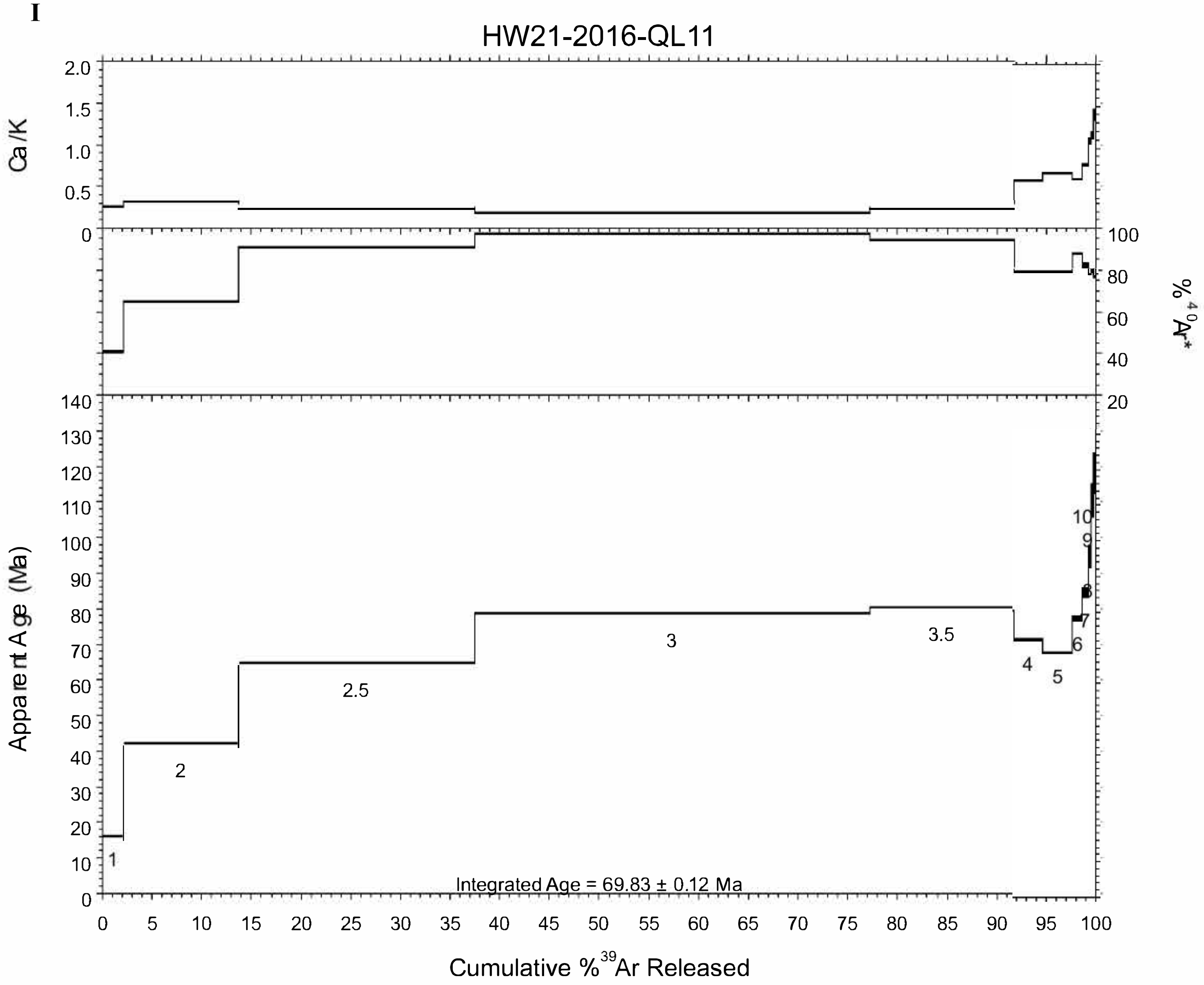

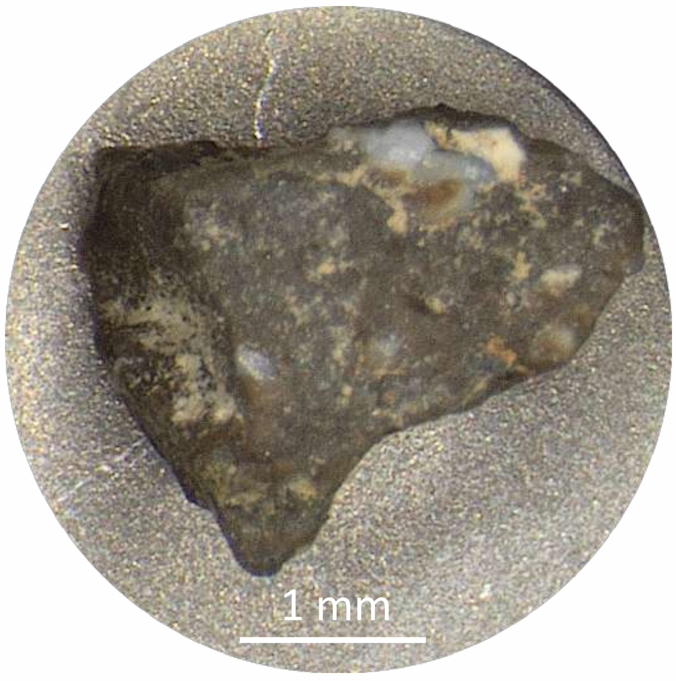

Fig. S3 continued.

J

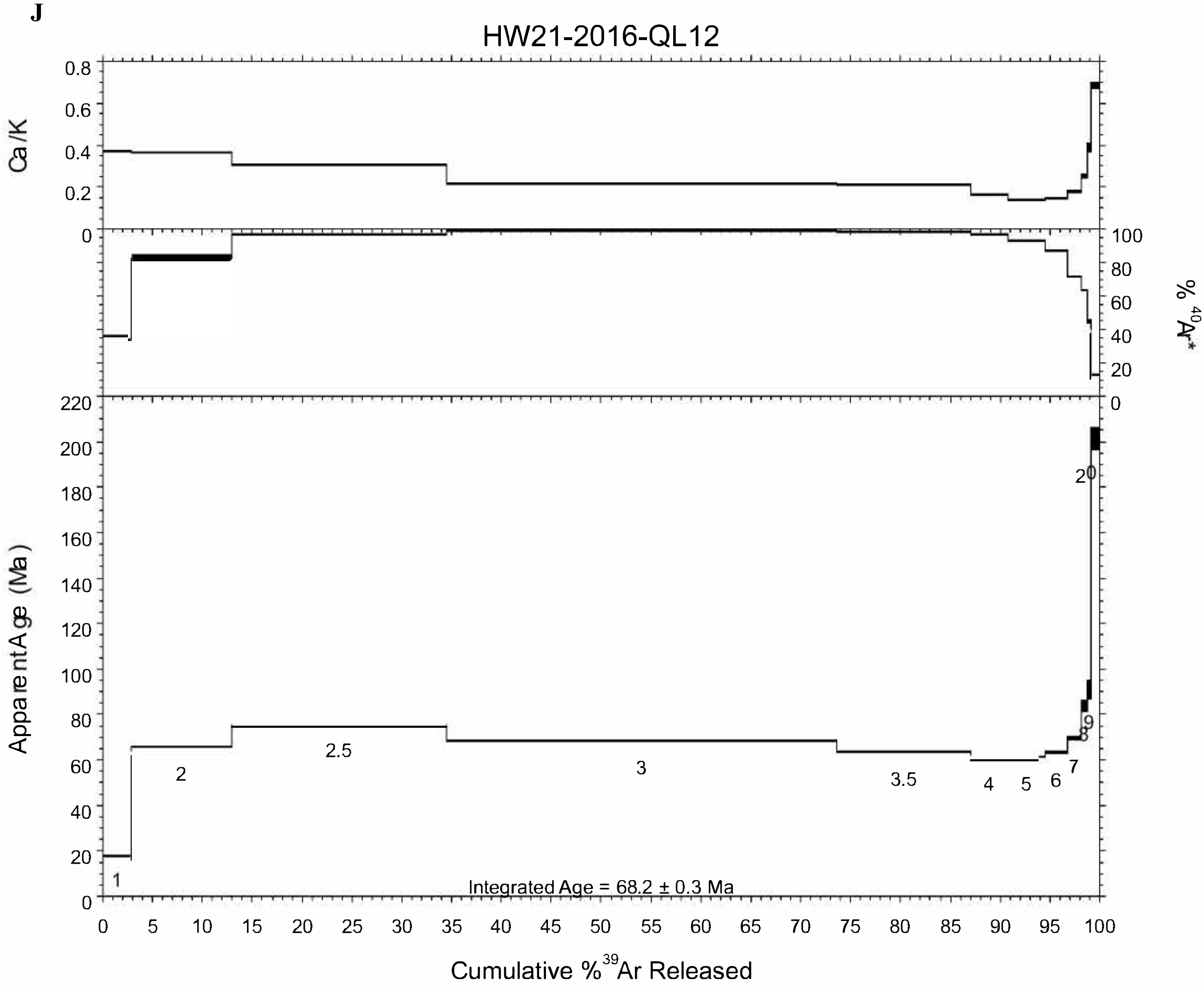

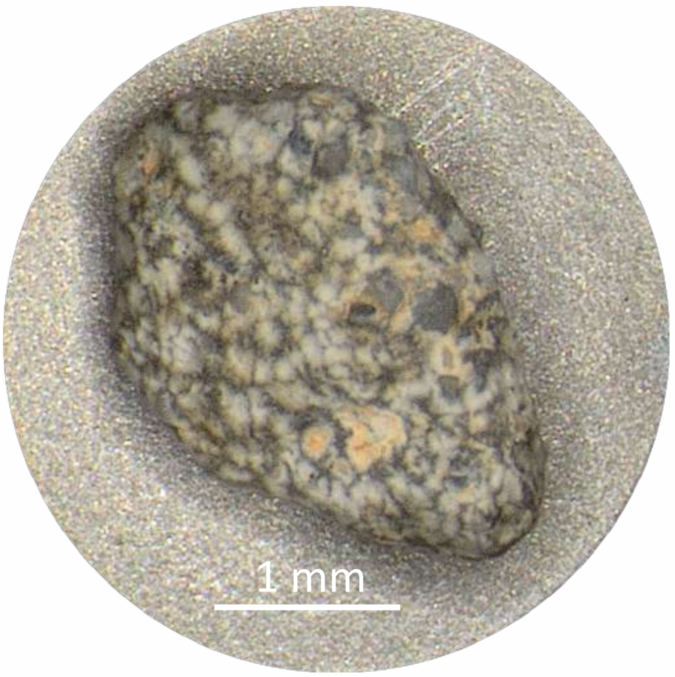

Fig. S3 continued.

K

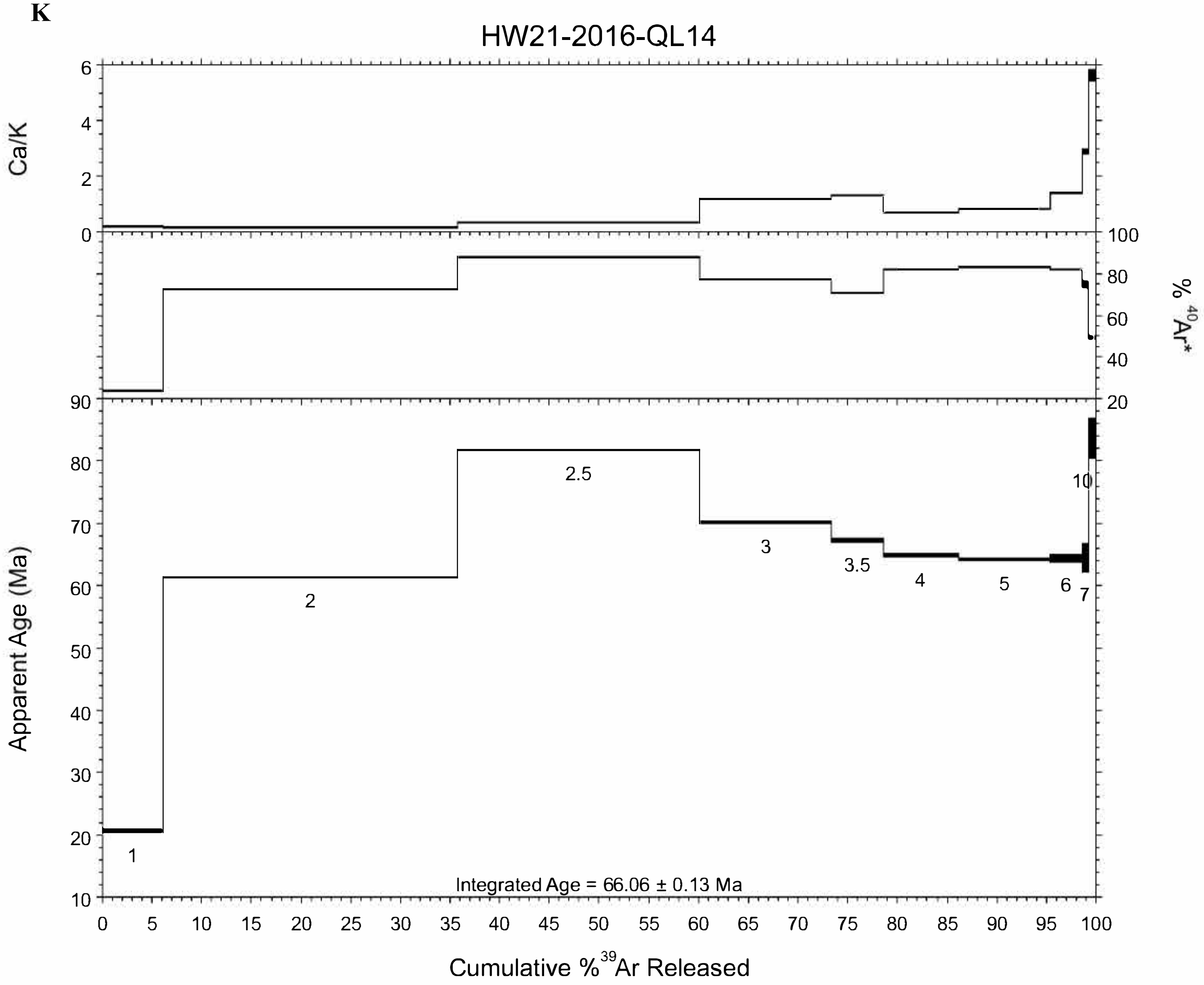

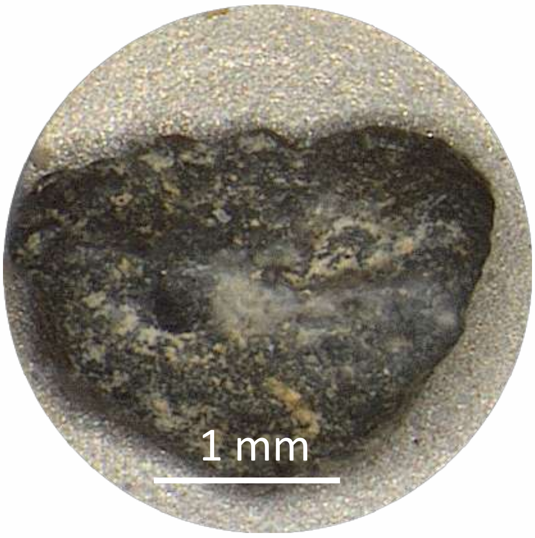

Fig. S3 continued.

L

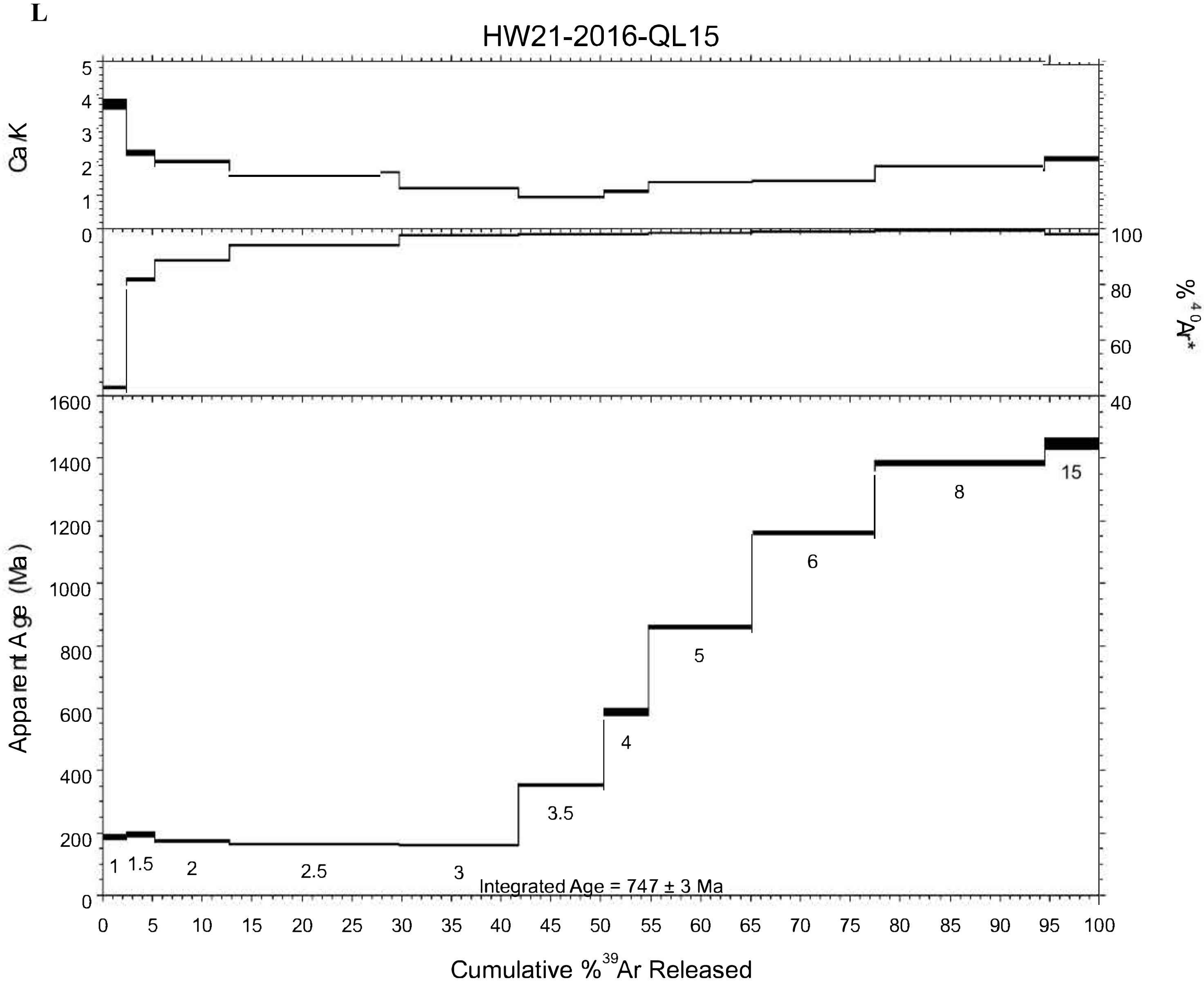

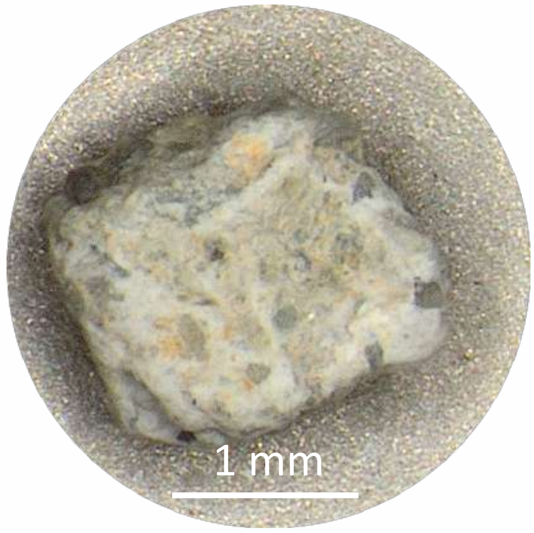

Fig. S3 continued.

M

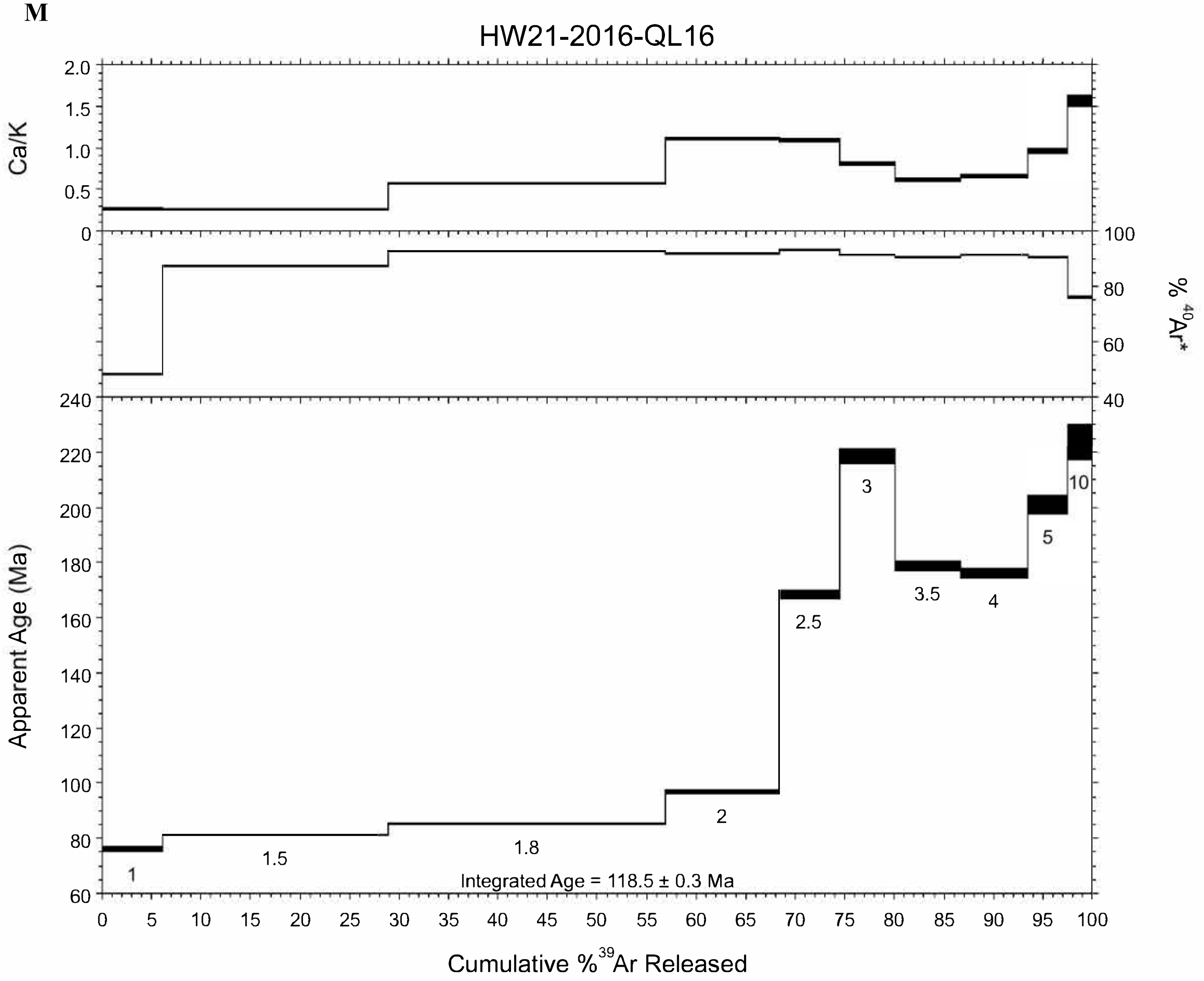

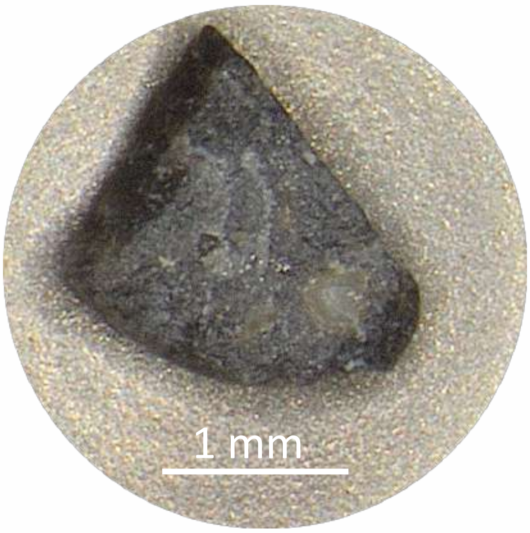

Fig. S3 continued.

N

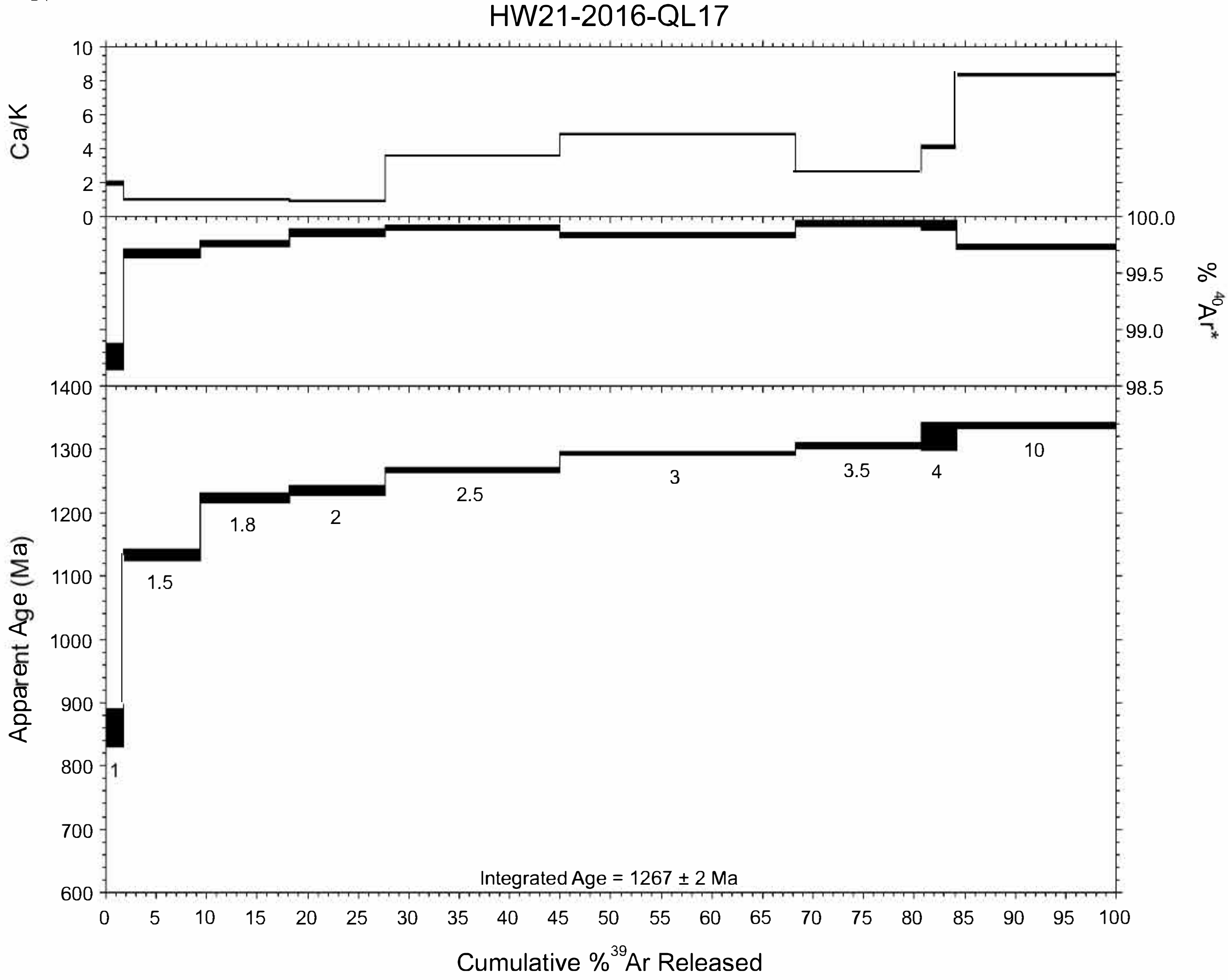

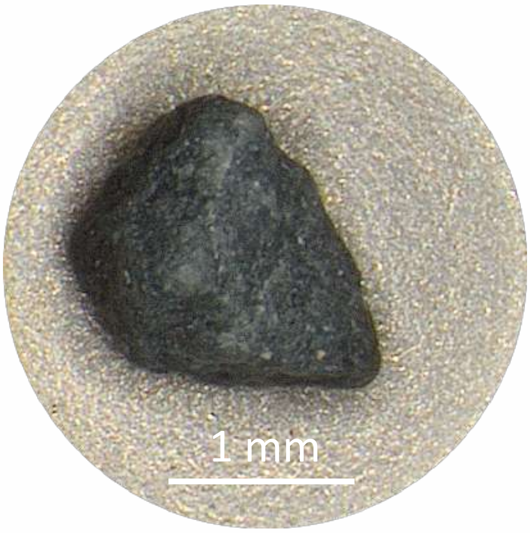

Fig. S3 continued.

O

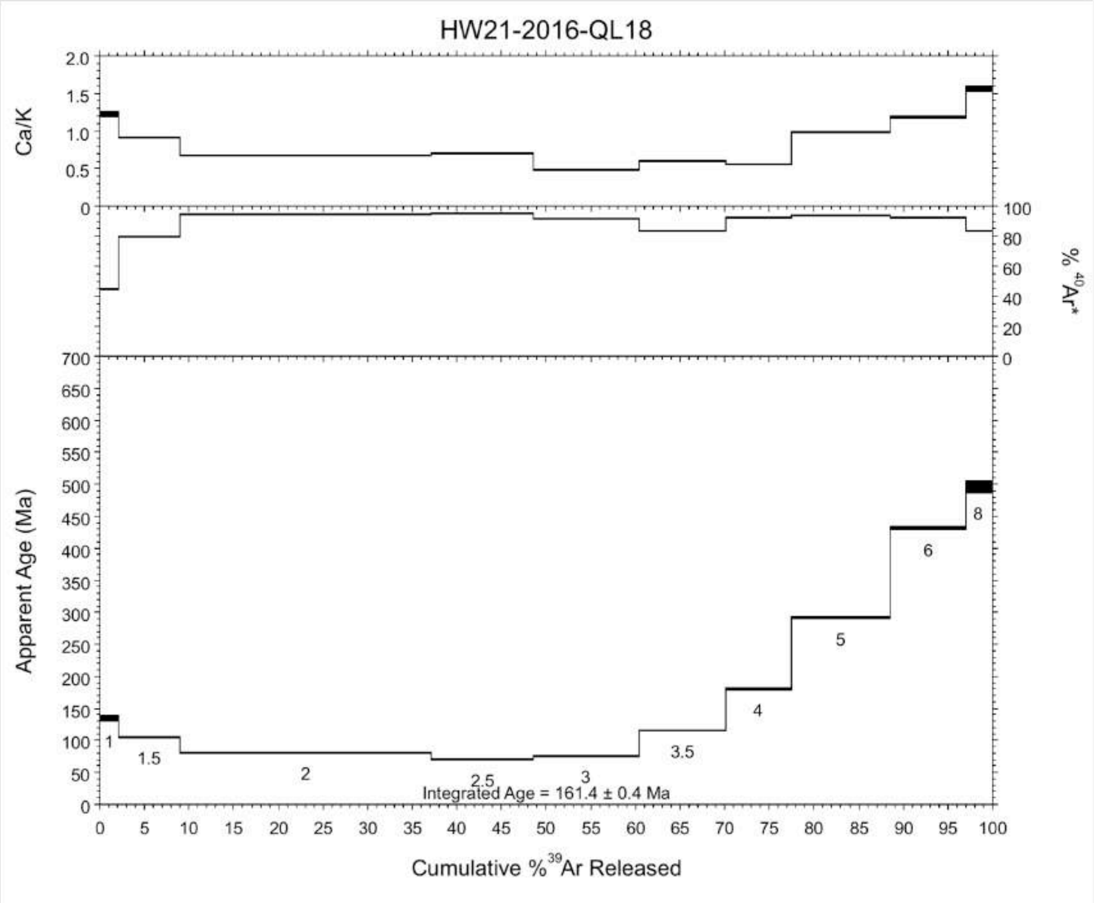

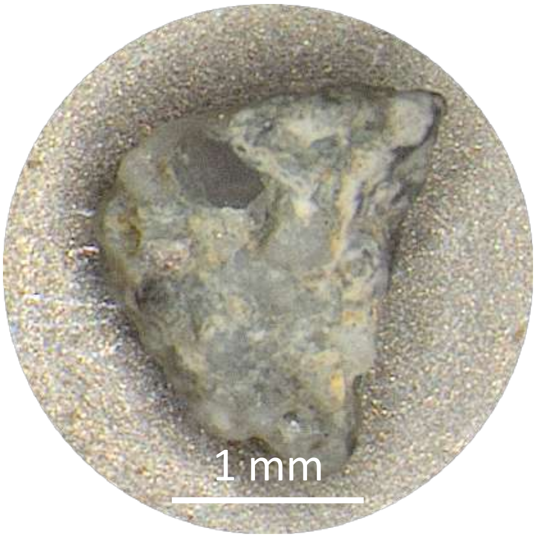

Fig. S3 continued.

P

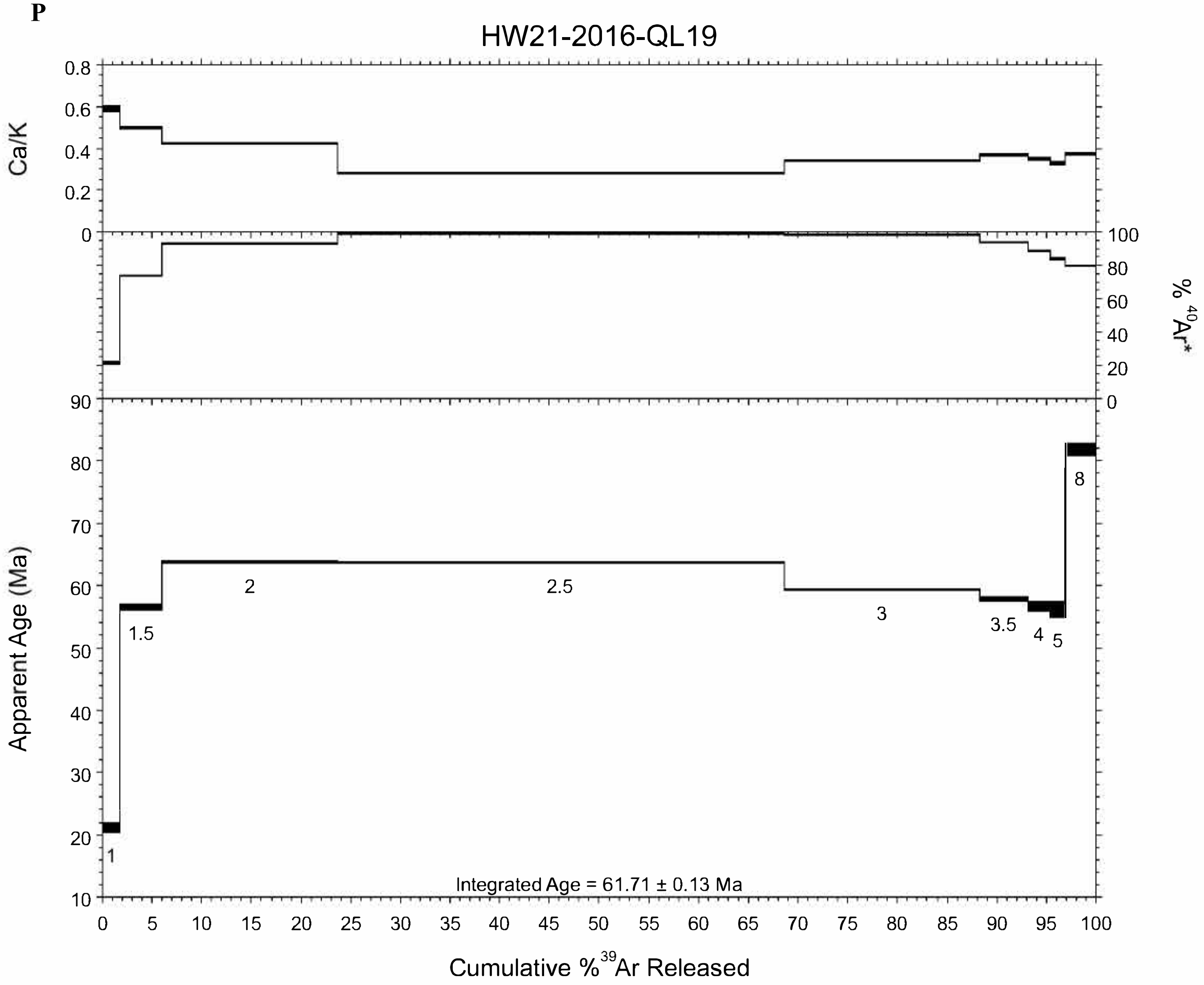

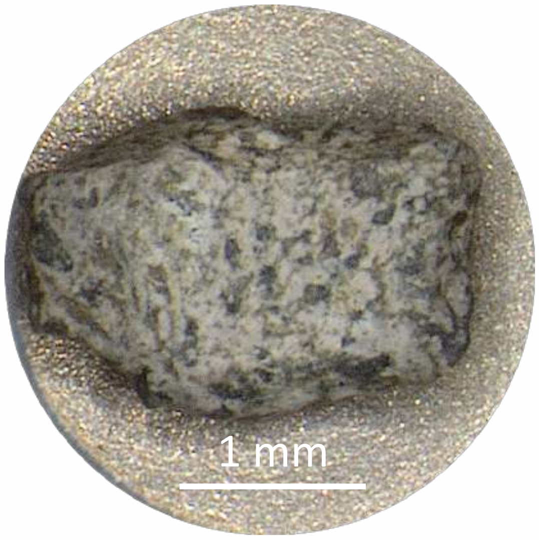

Fig. S3 continued.

Q

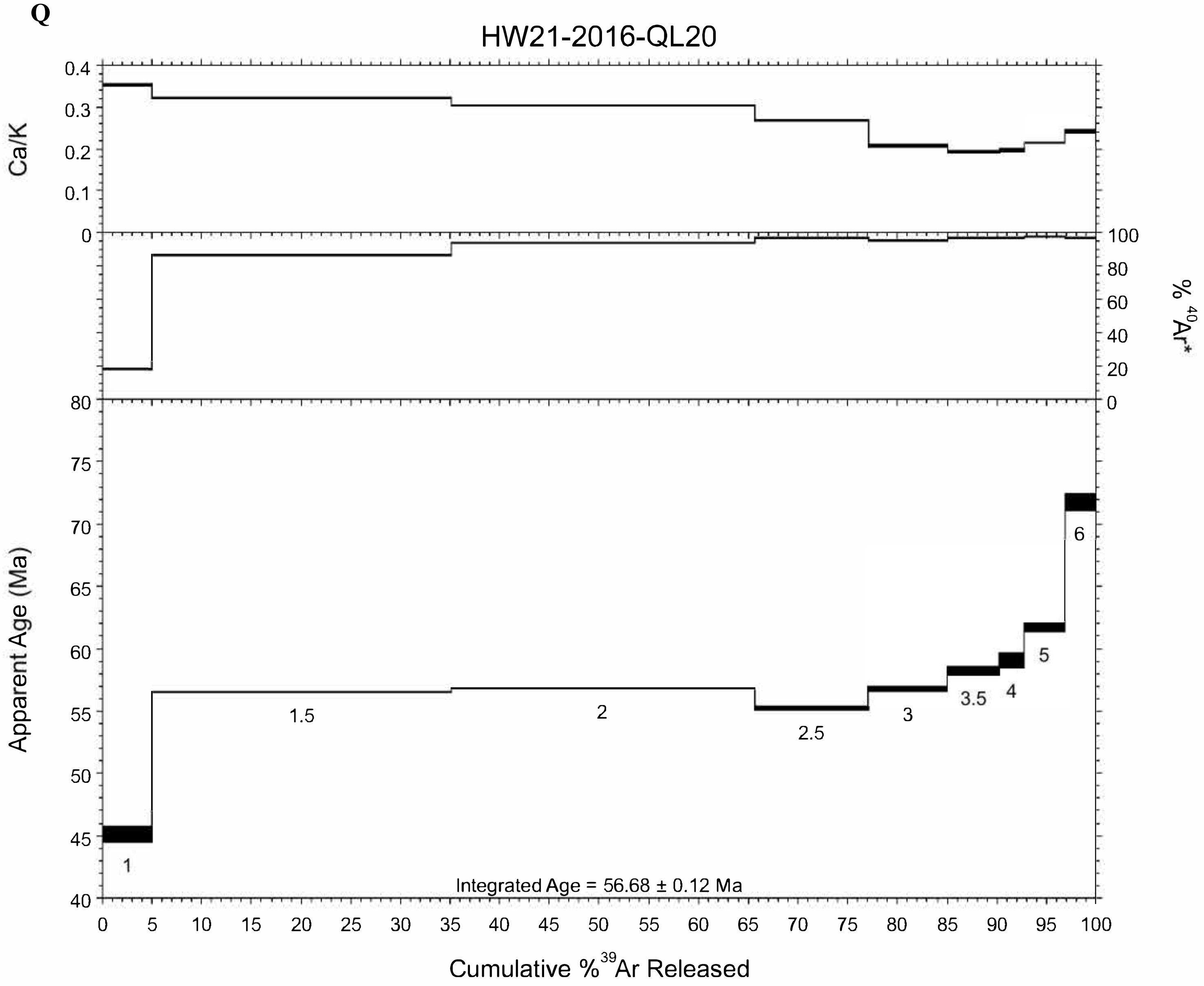

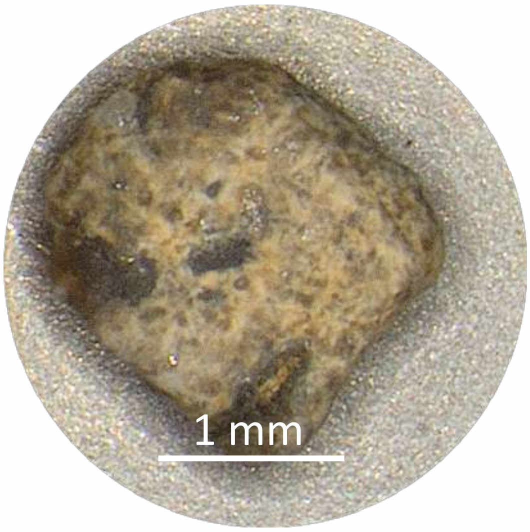

Fig. S3 continued.

R

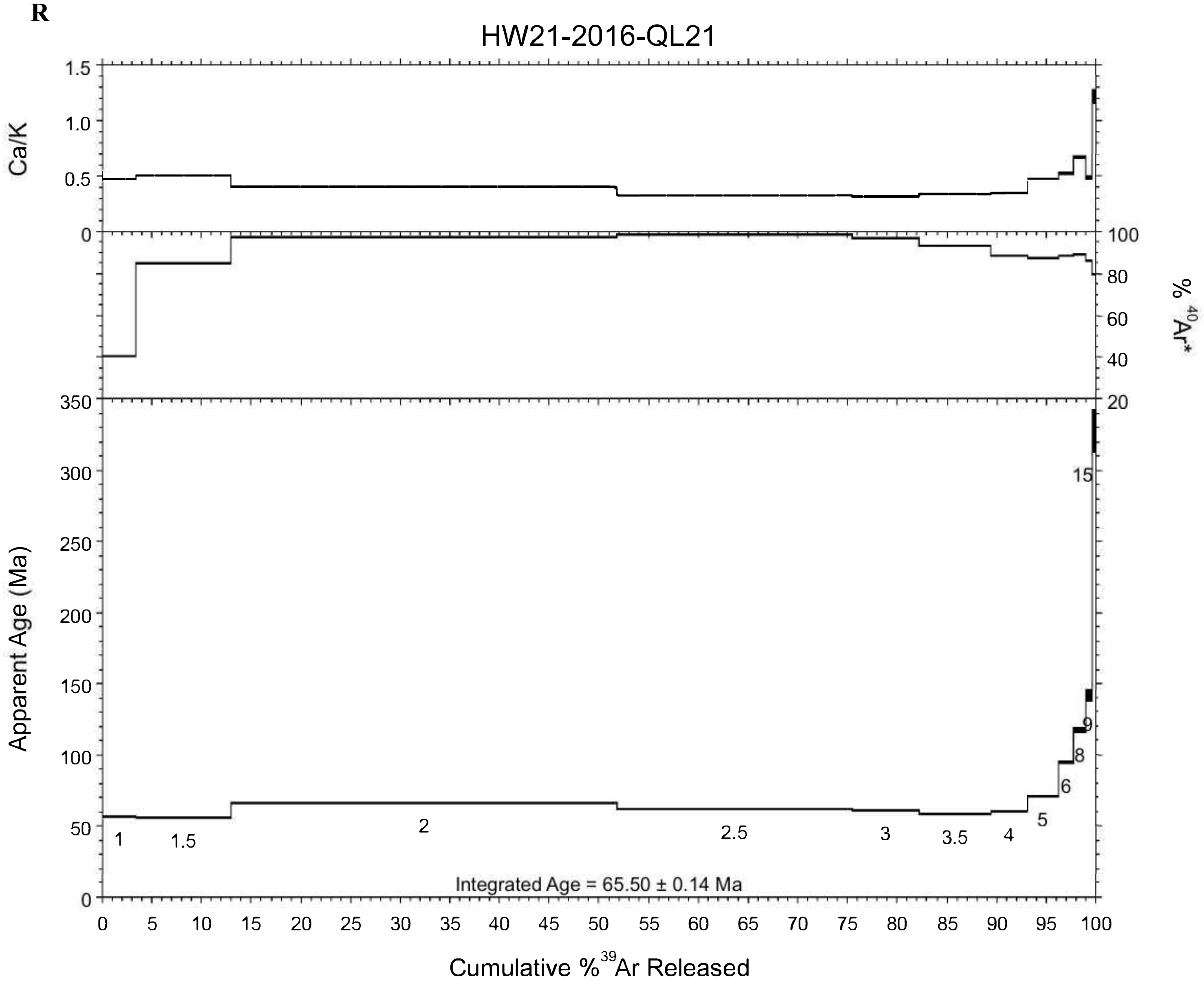

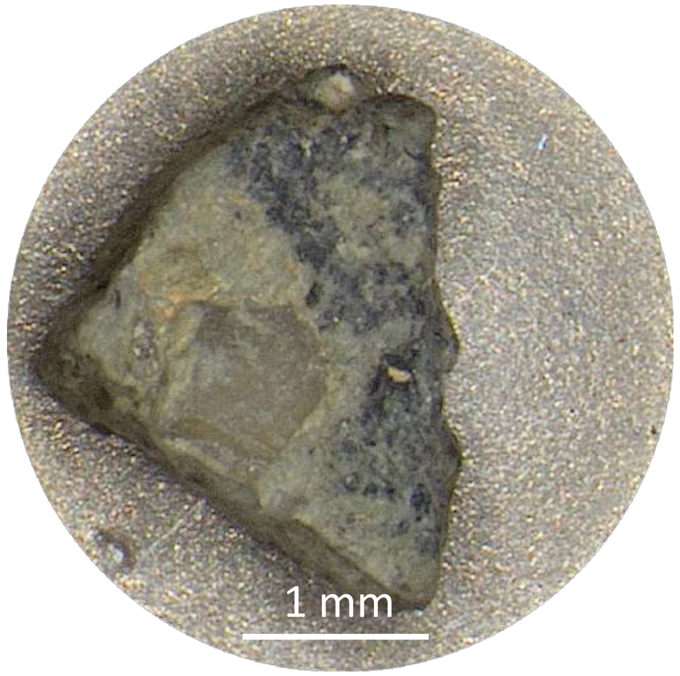

Fig. S3 continued.

S

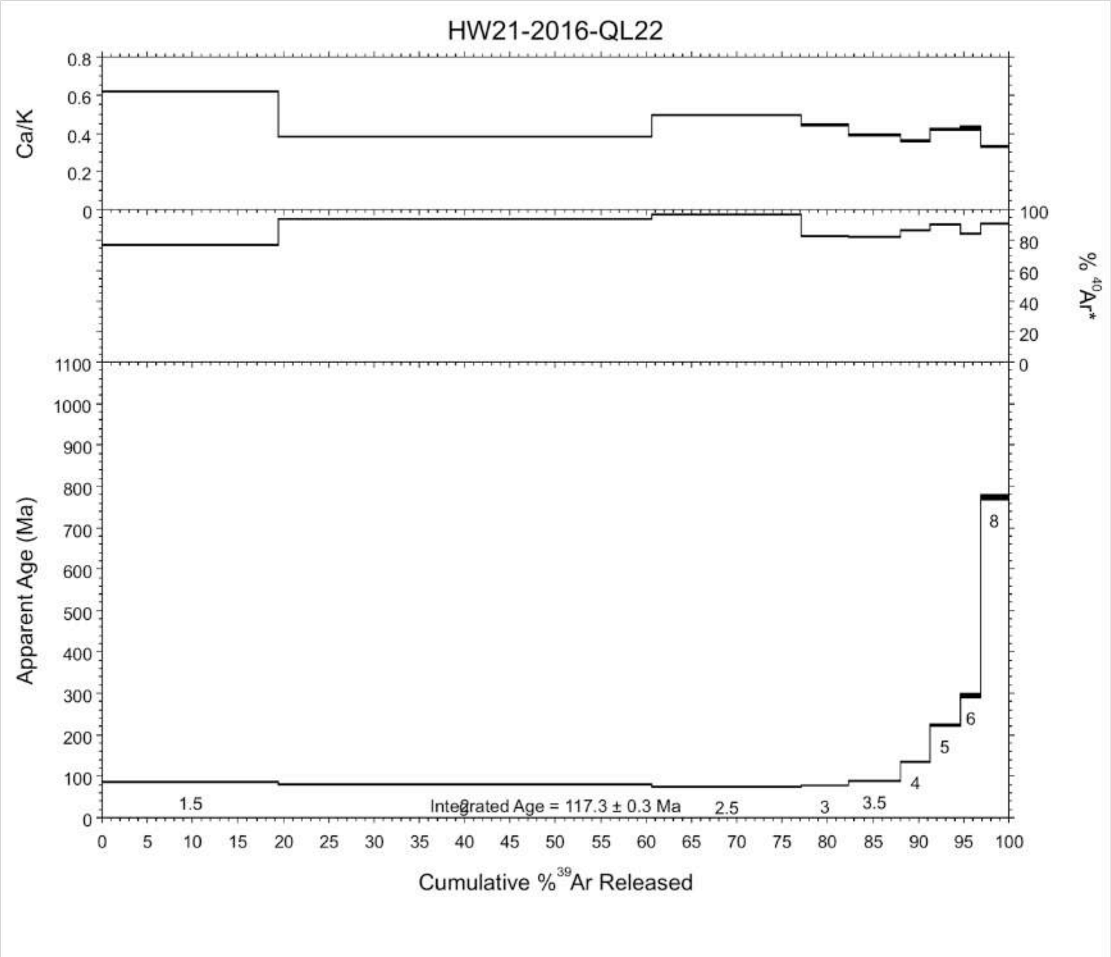

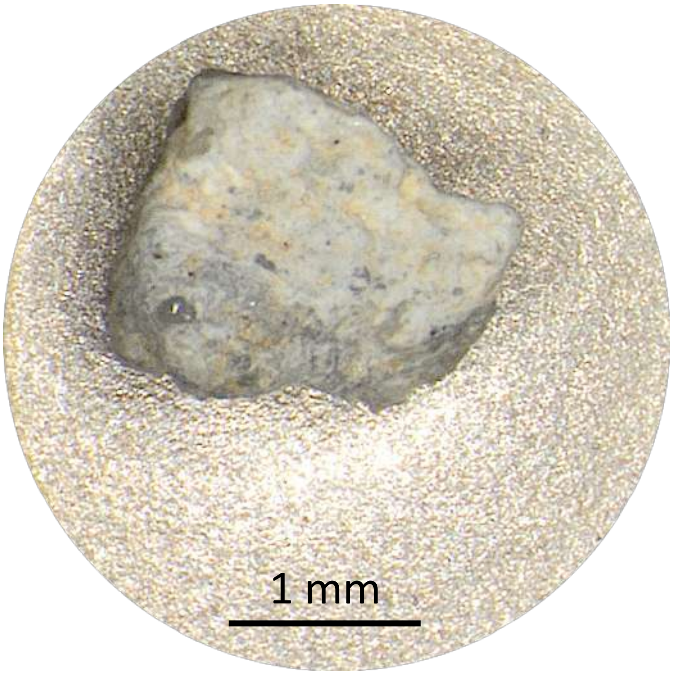

Fig. S3 continued.

T

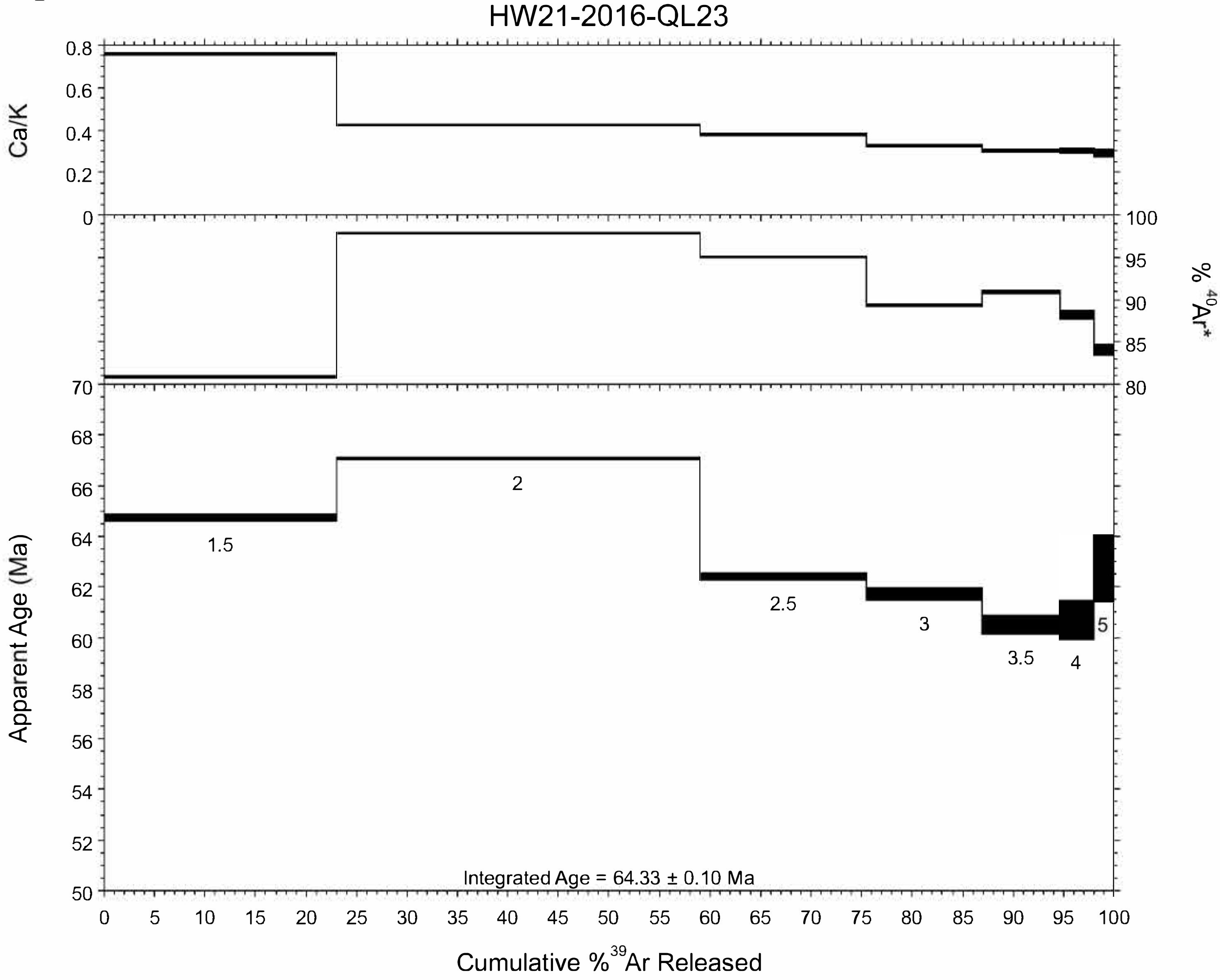

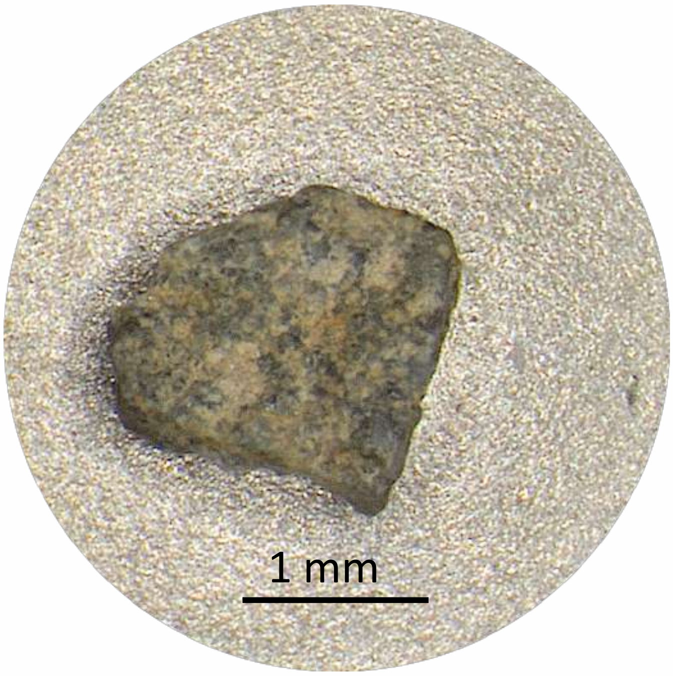

Fig. S3 continued.

U

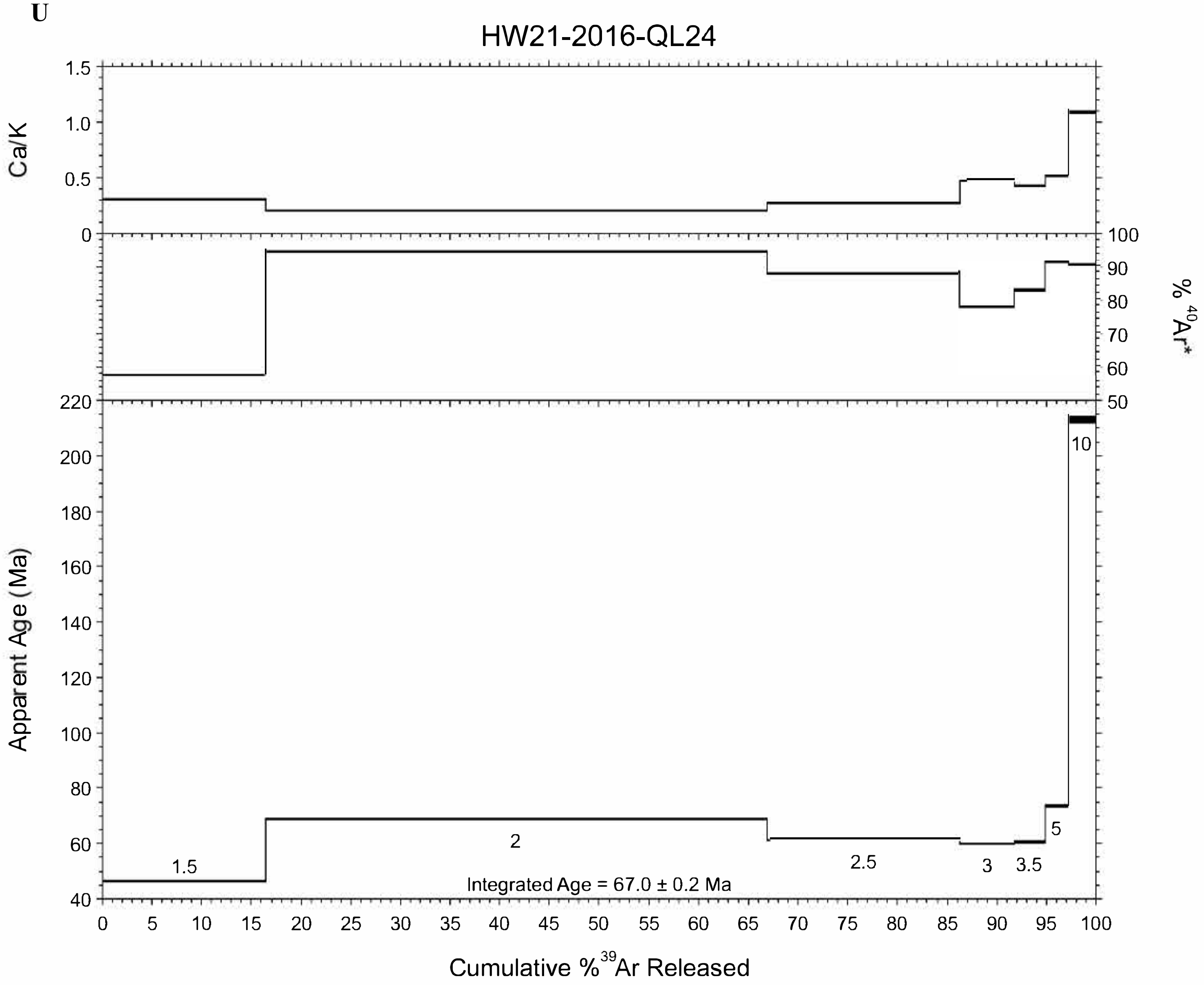

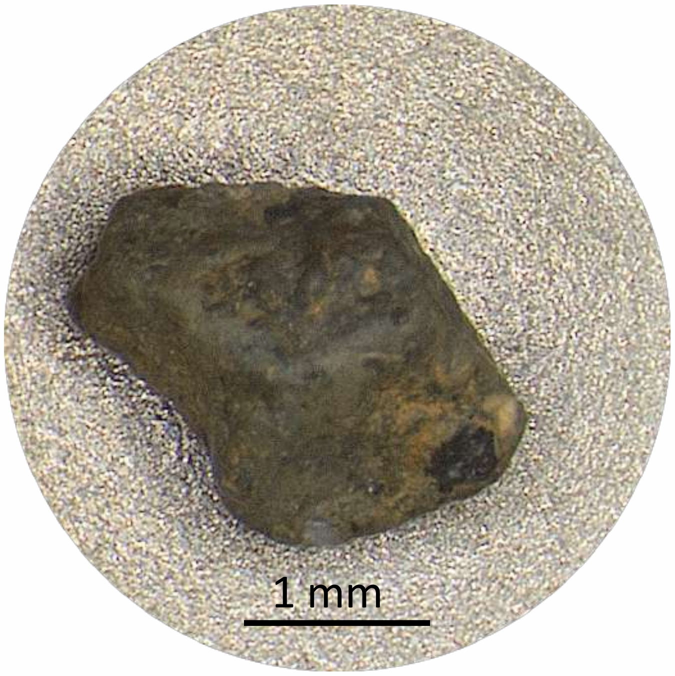

Fig. S3 continued.

V

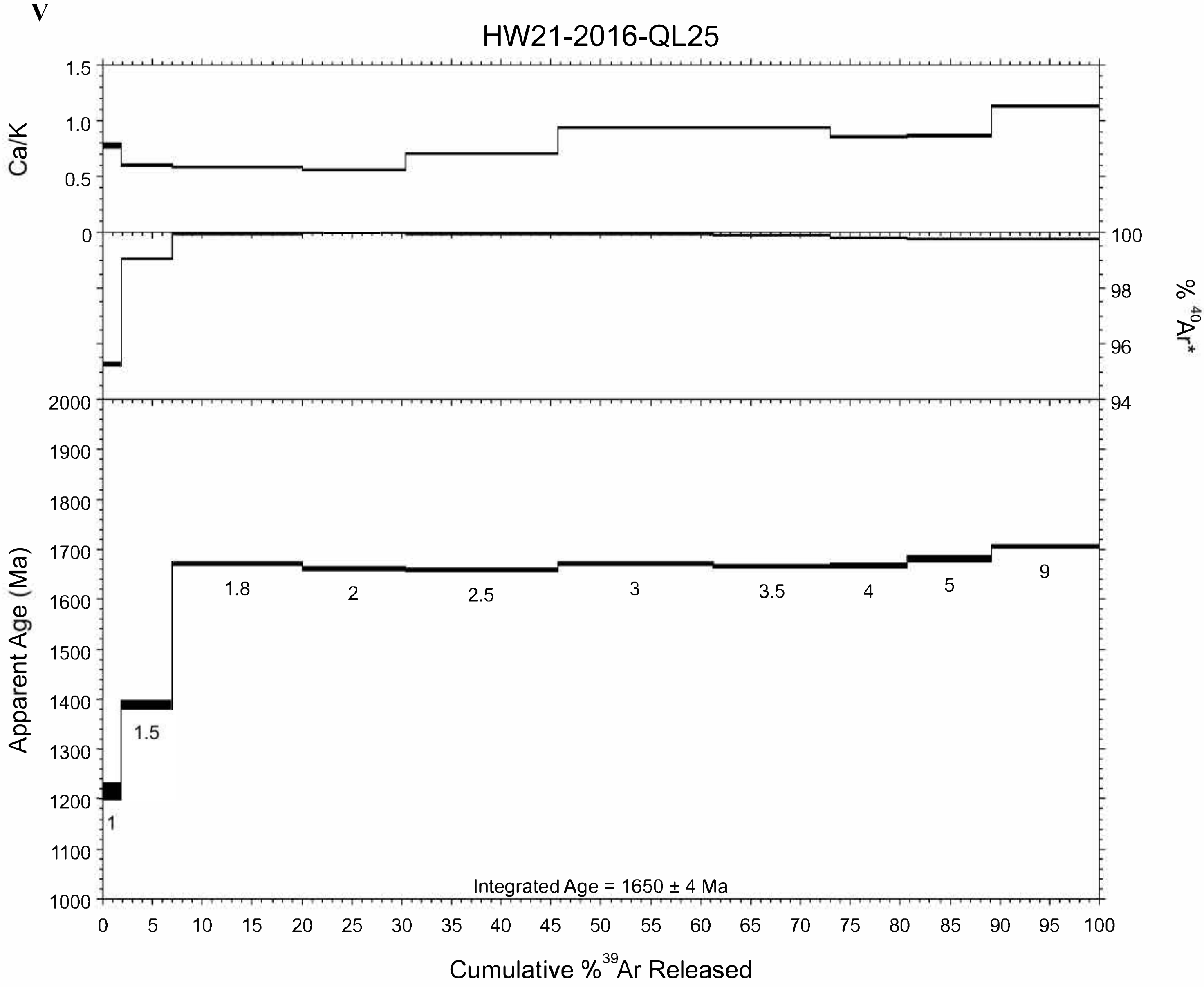

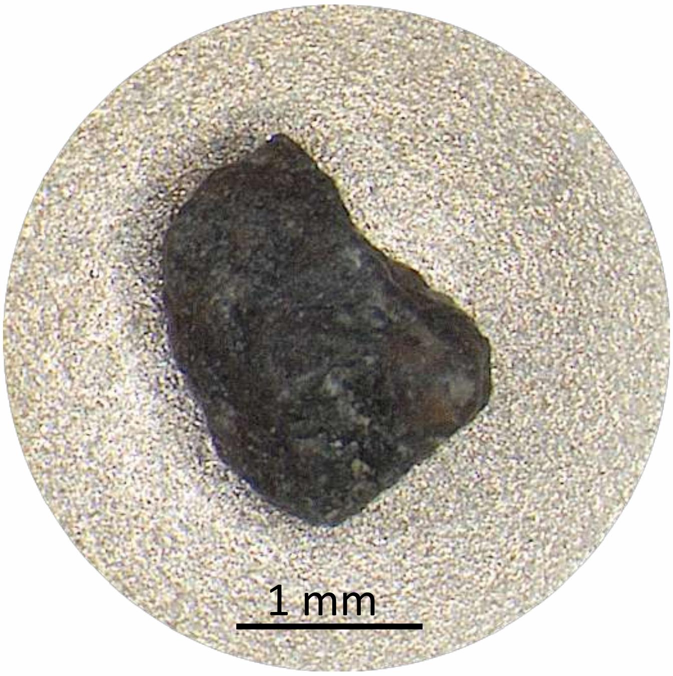

Fig. S3 continued.

W

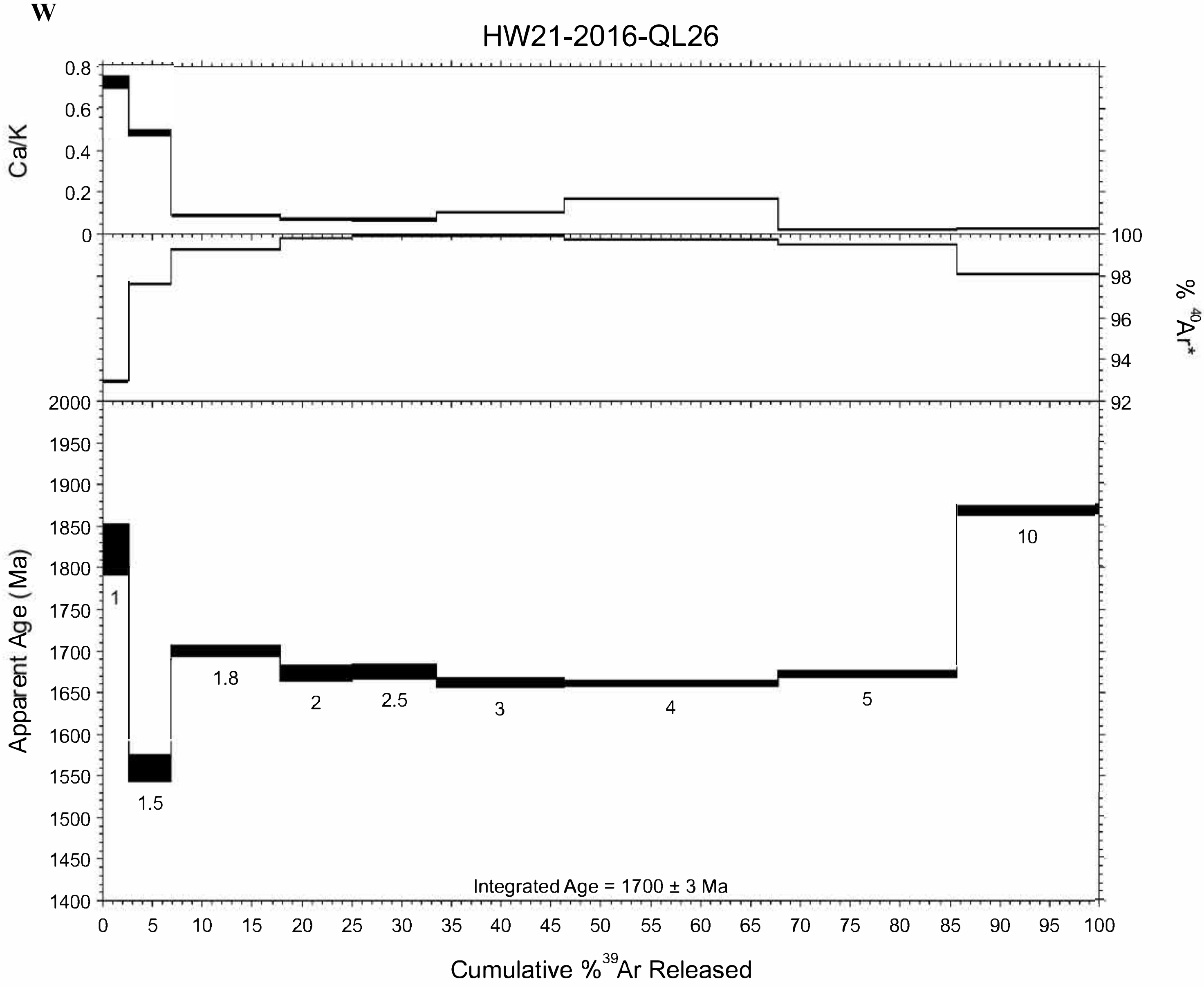

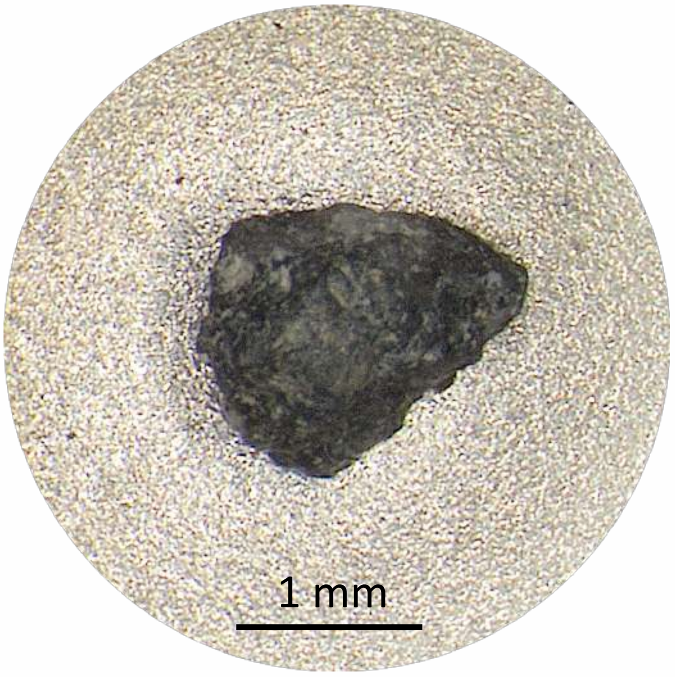

Fig. S3 continued.

X

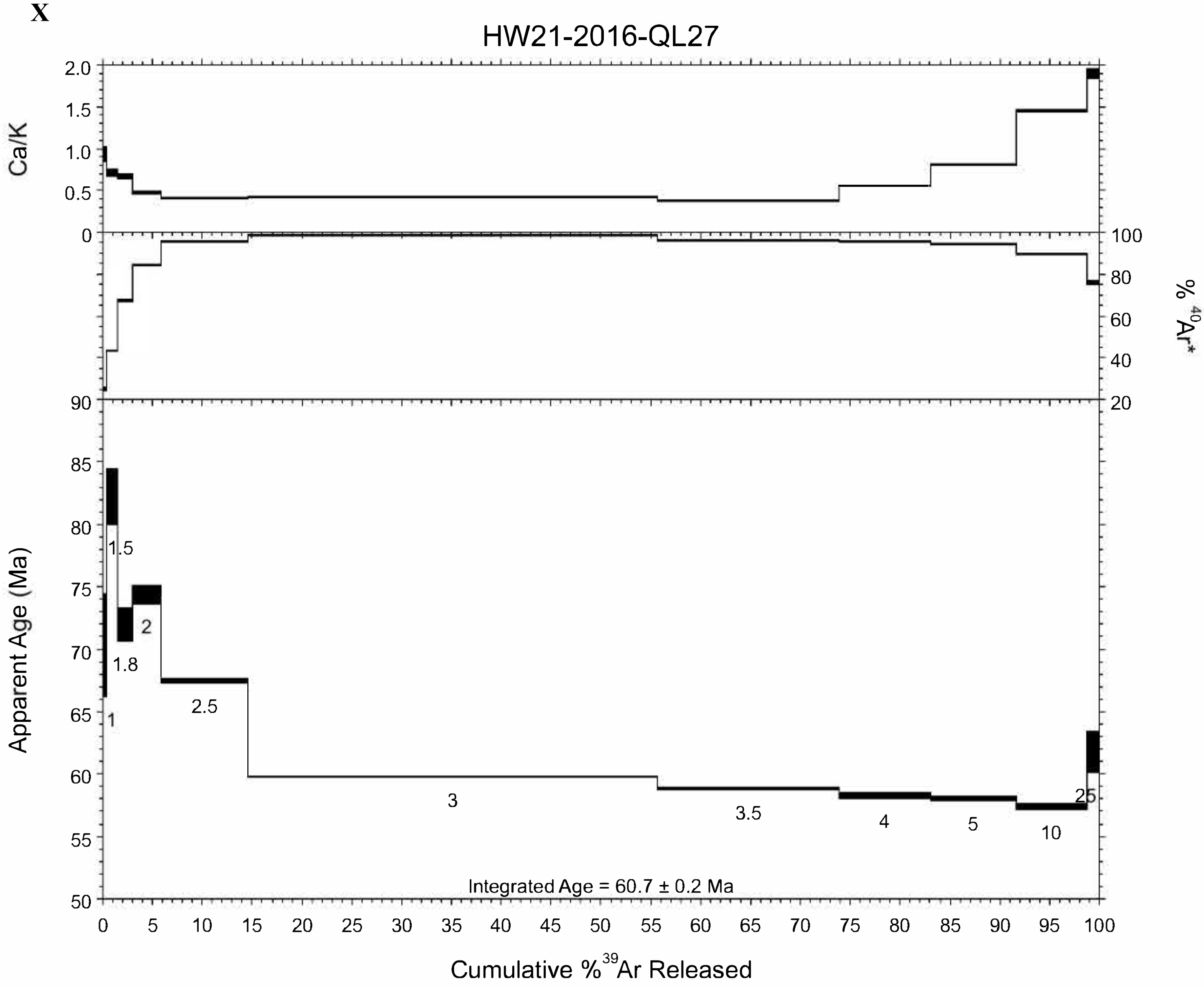

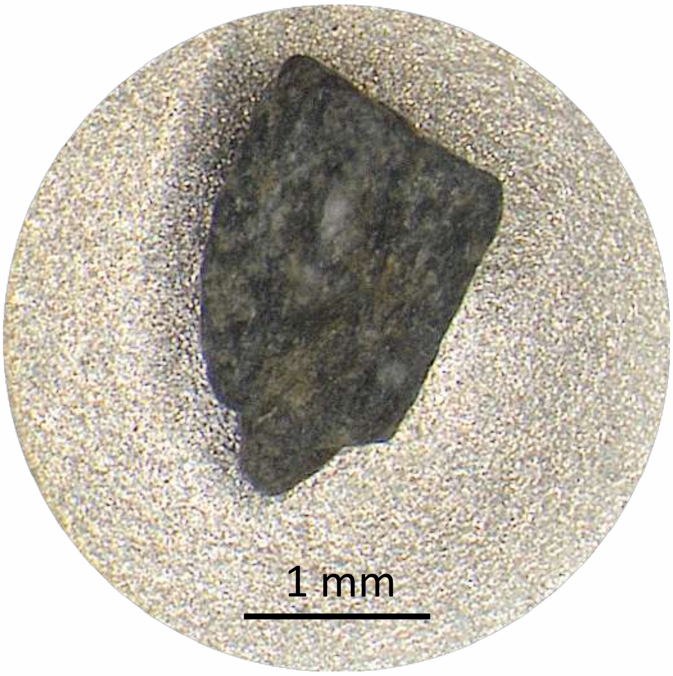

Fig. S3 continued.

Y

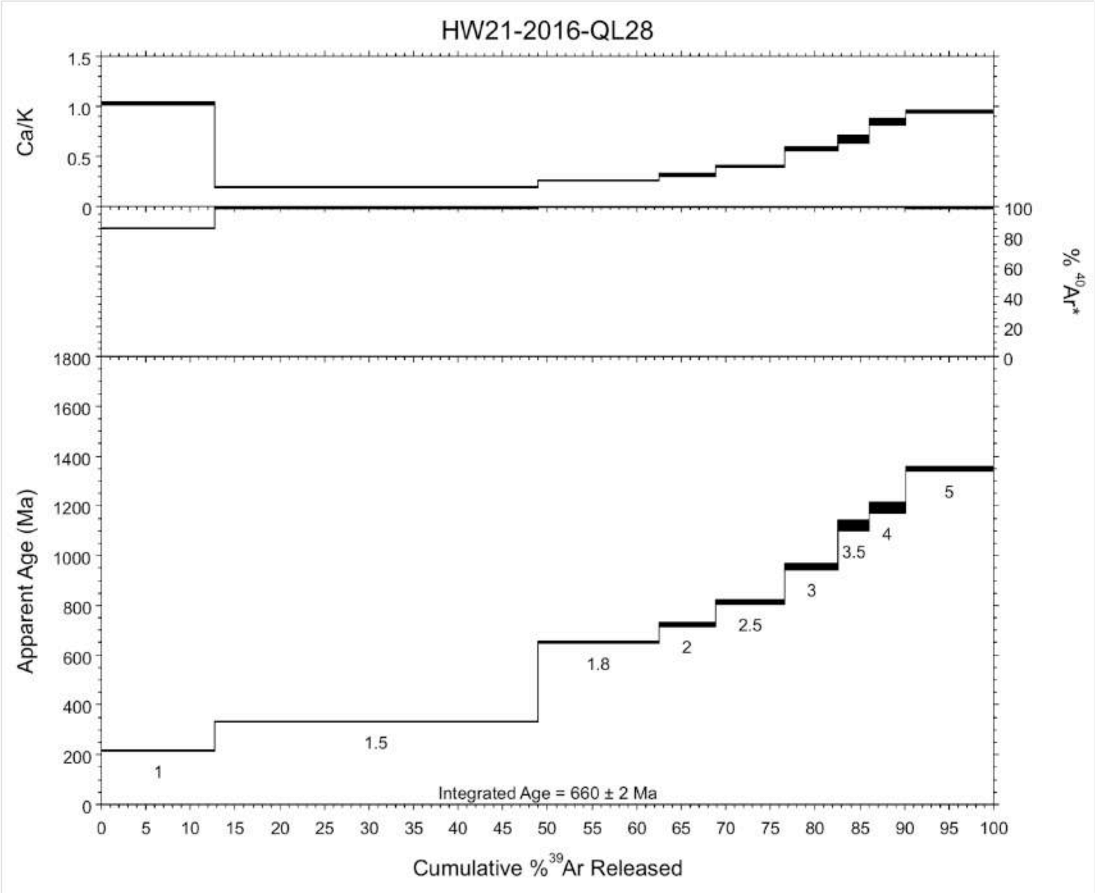

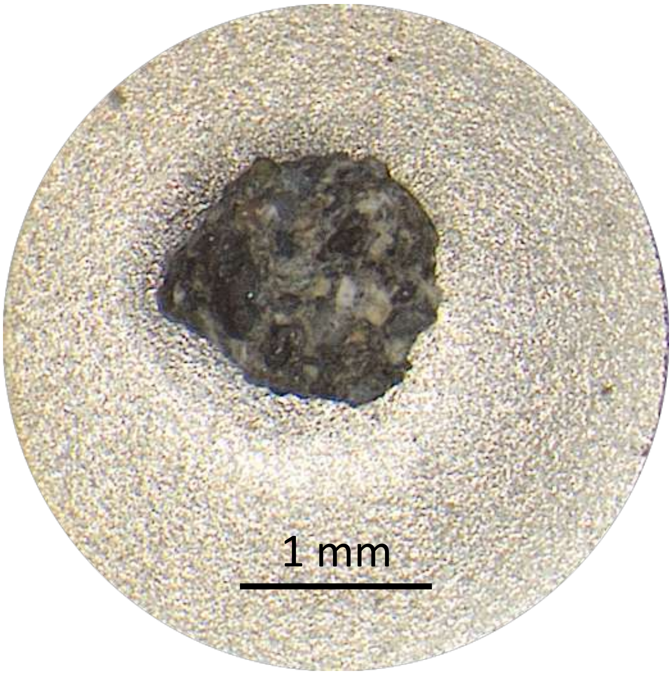

Fig. S3 continued.

Z

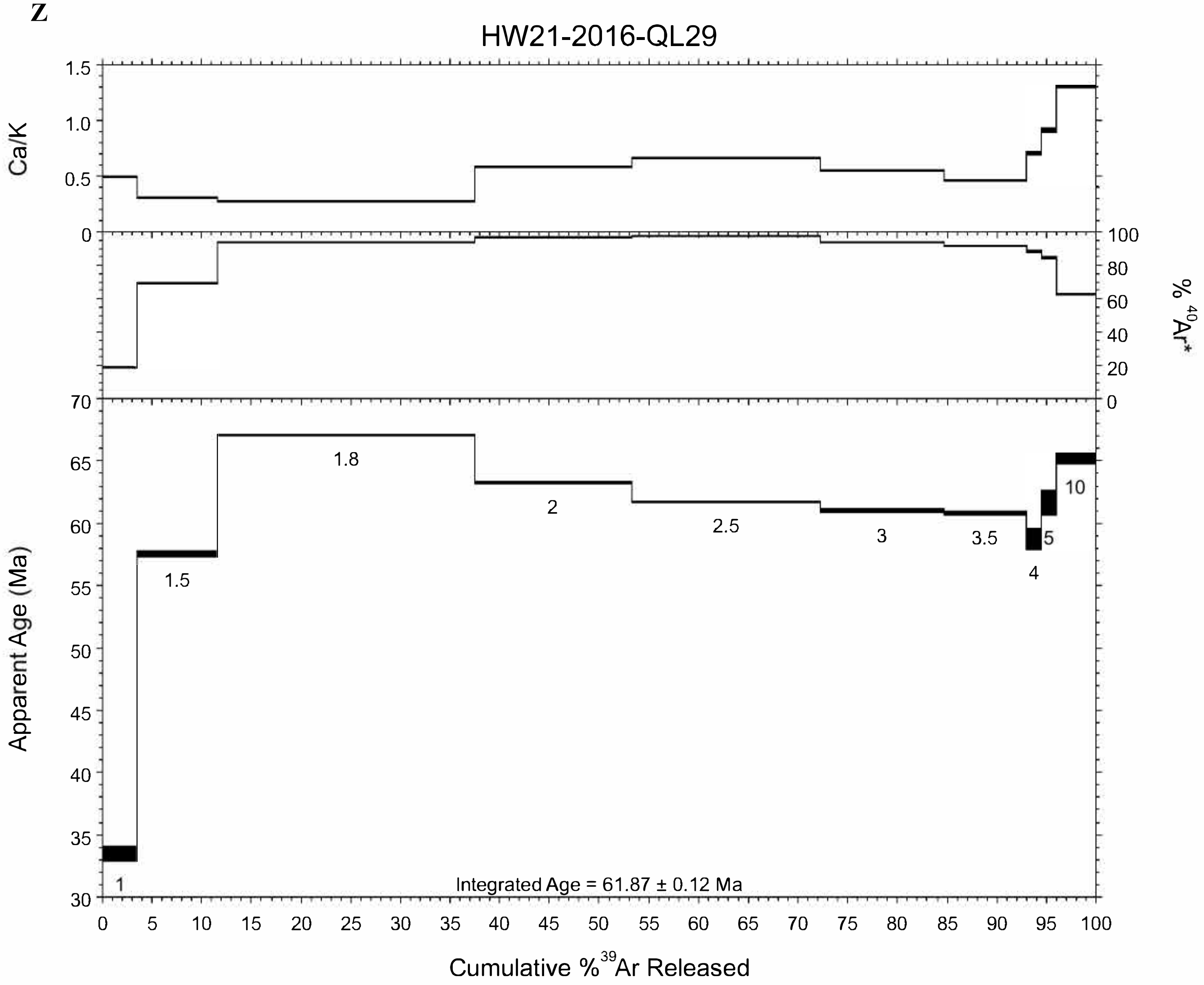

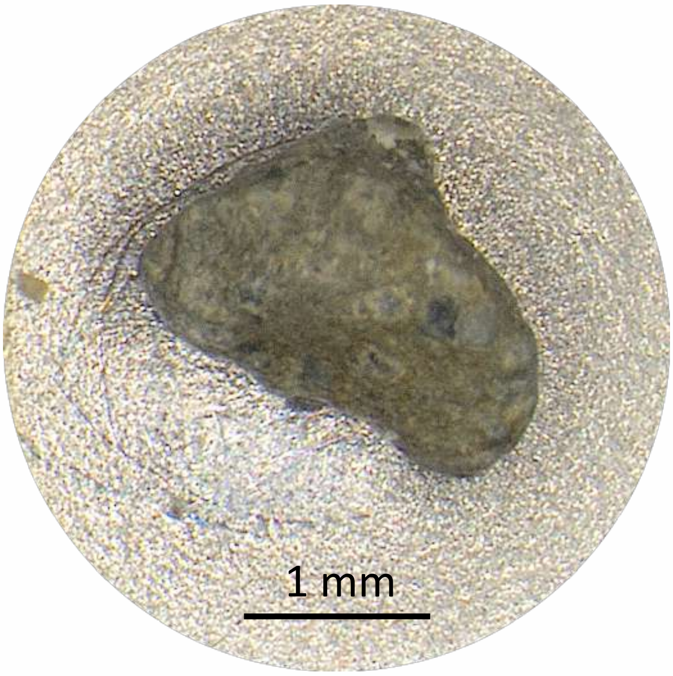

Fig. S3 continued.

AA

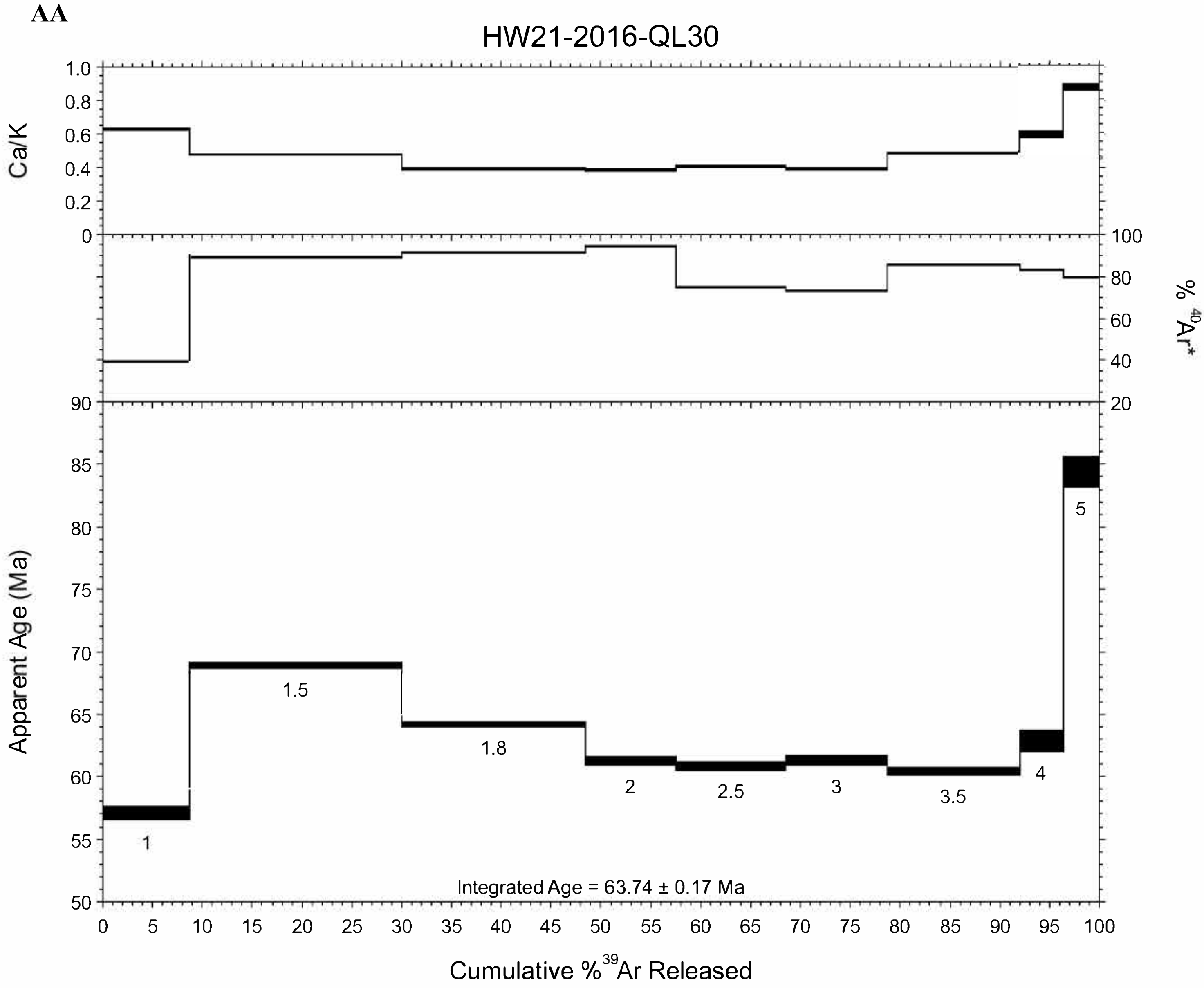

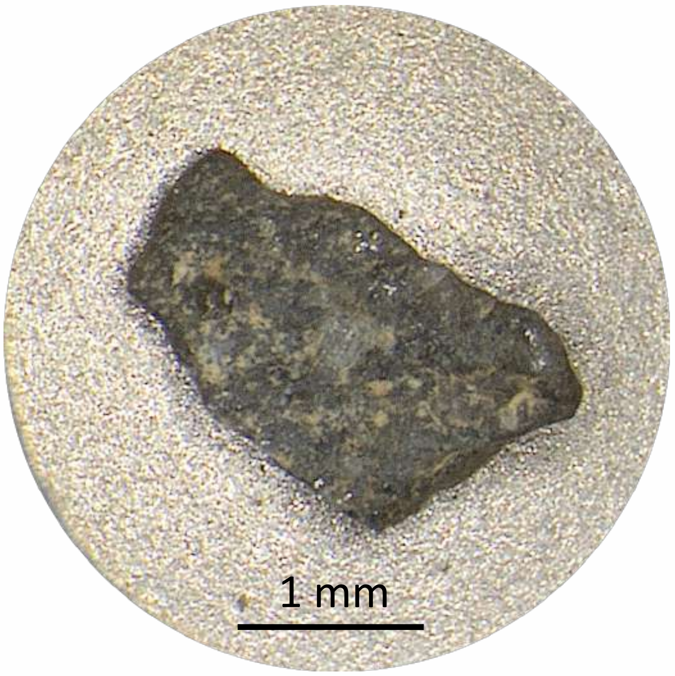

Fig. S3 continued.

AB

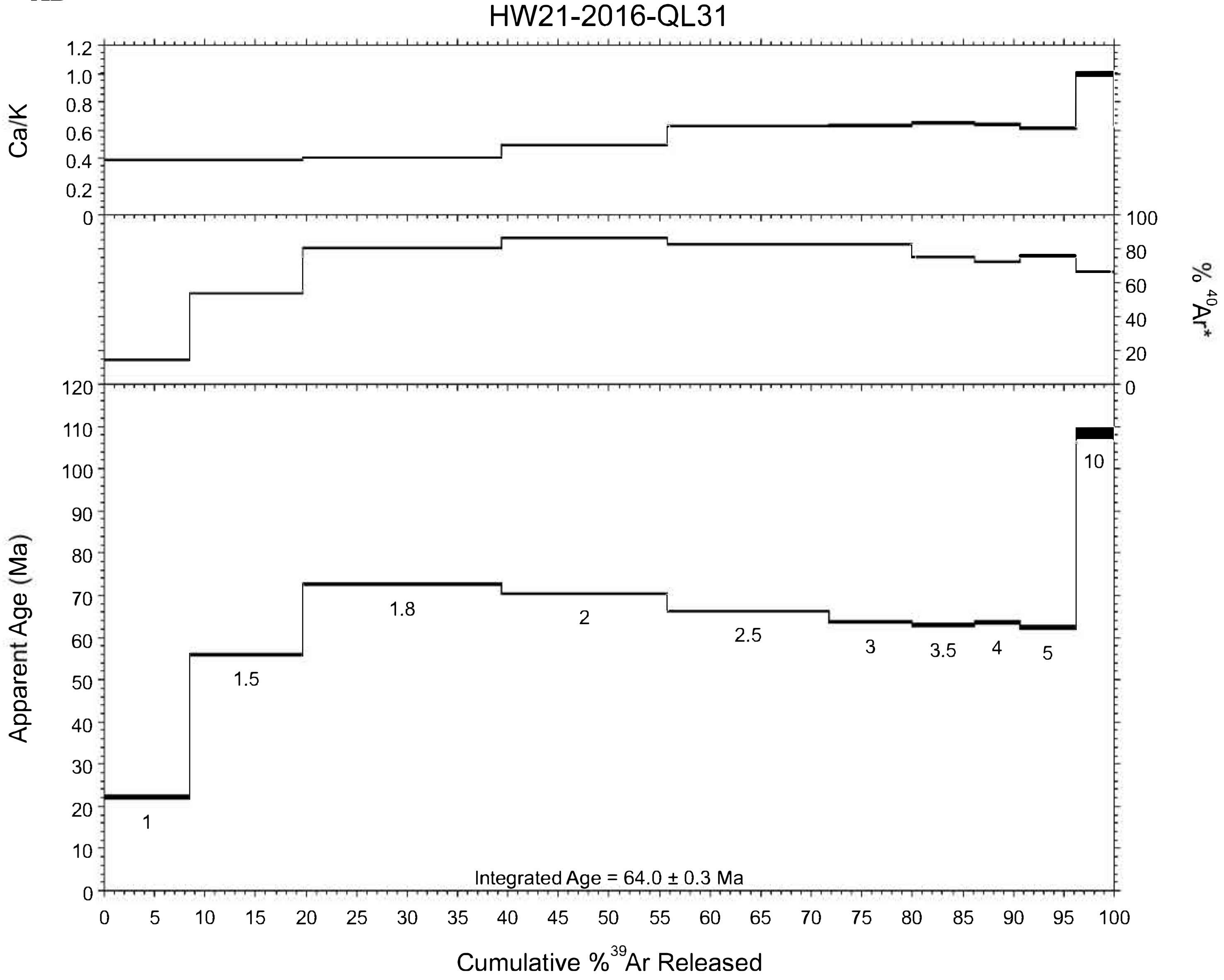

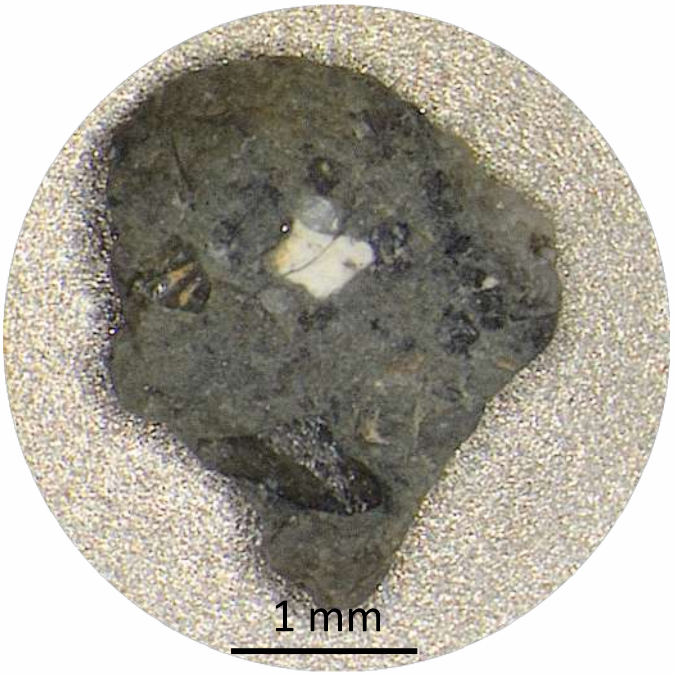

Fig. S3 continued.

AC

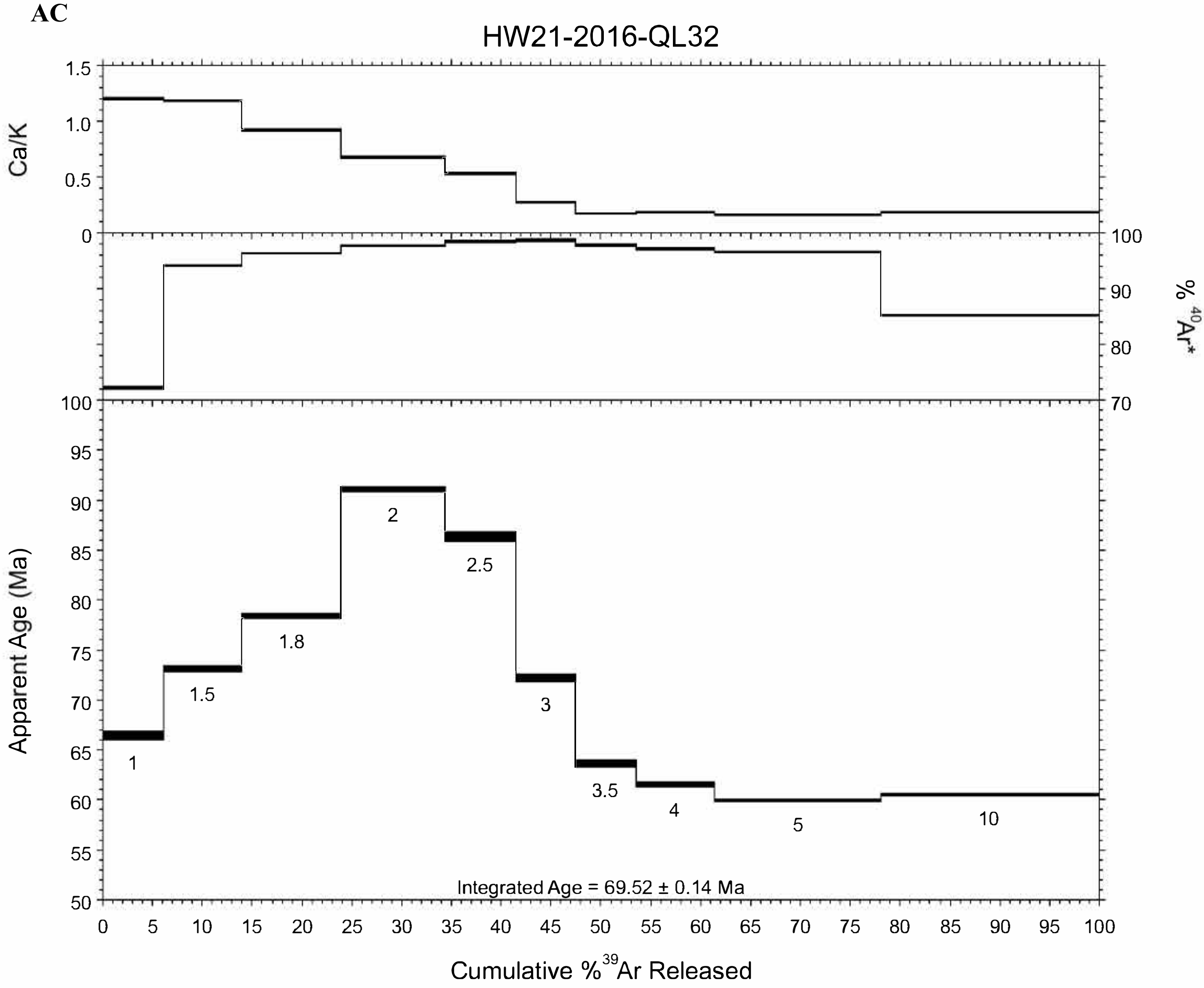

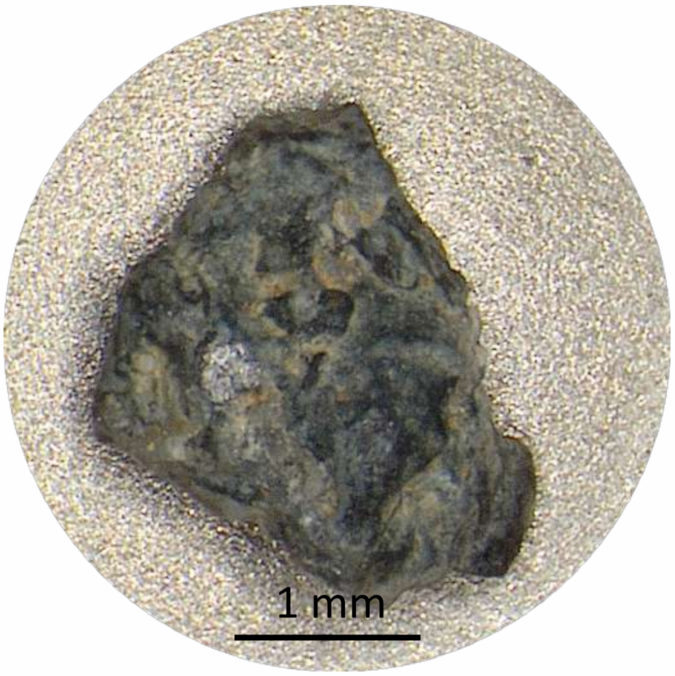

Fig. S3 continued.

AD

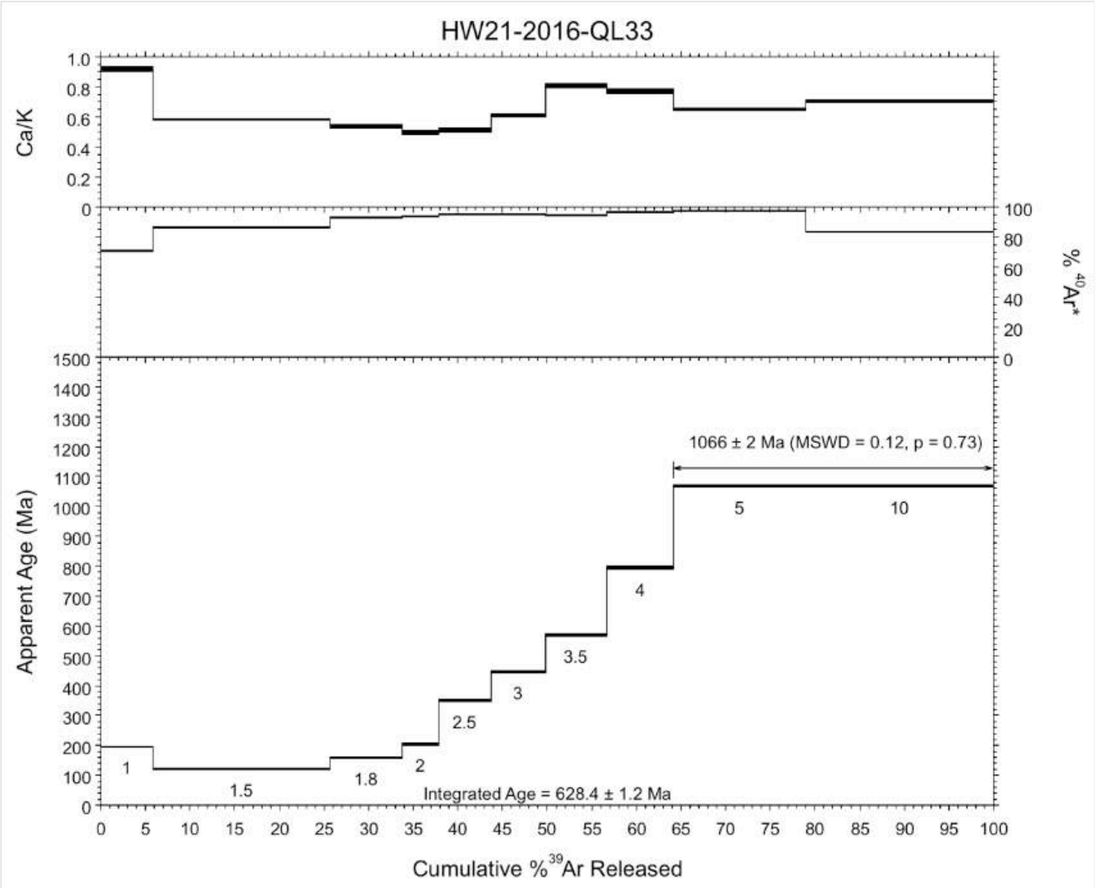

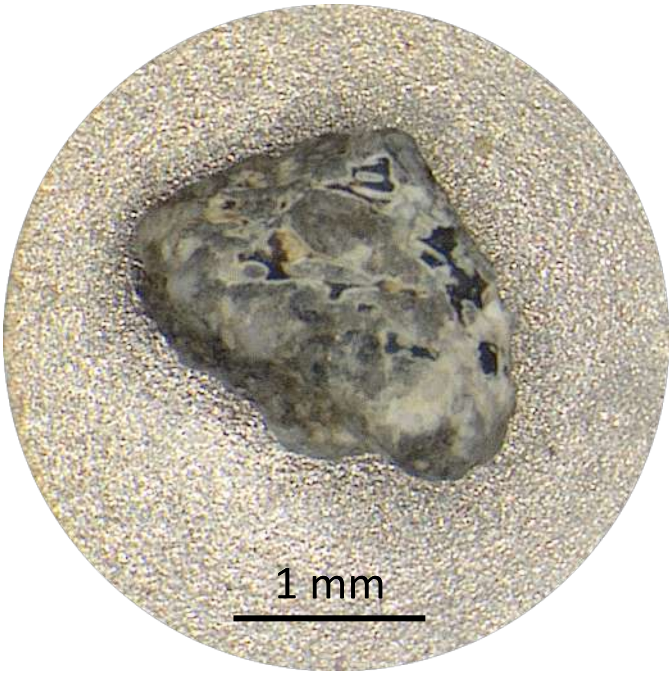

Fig. S3 continued.

AE

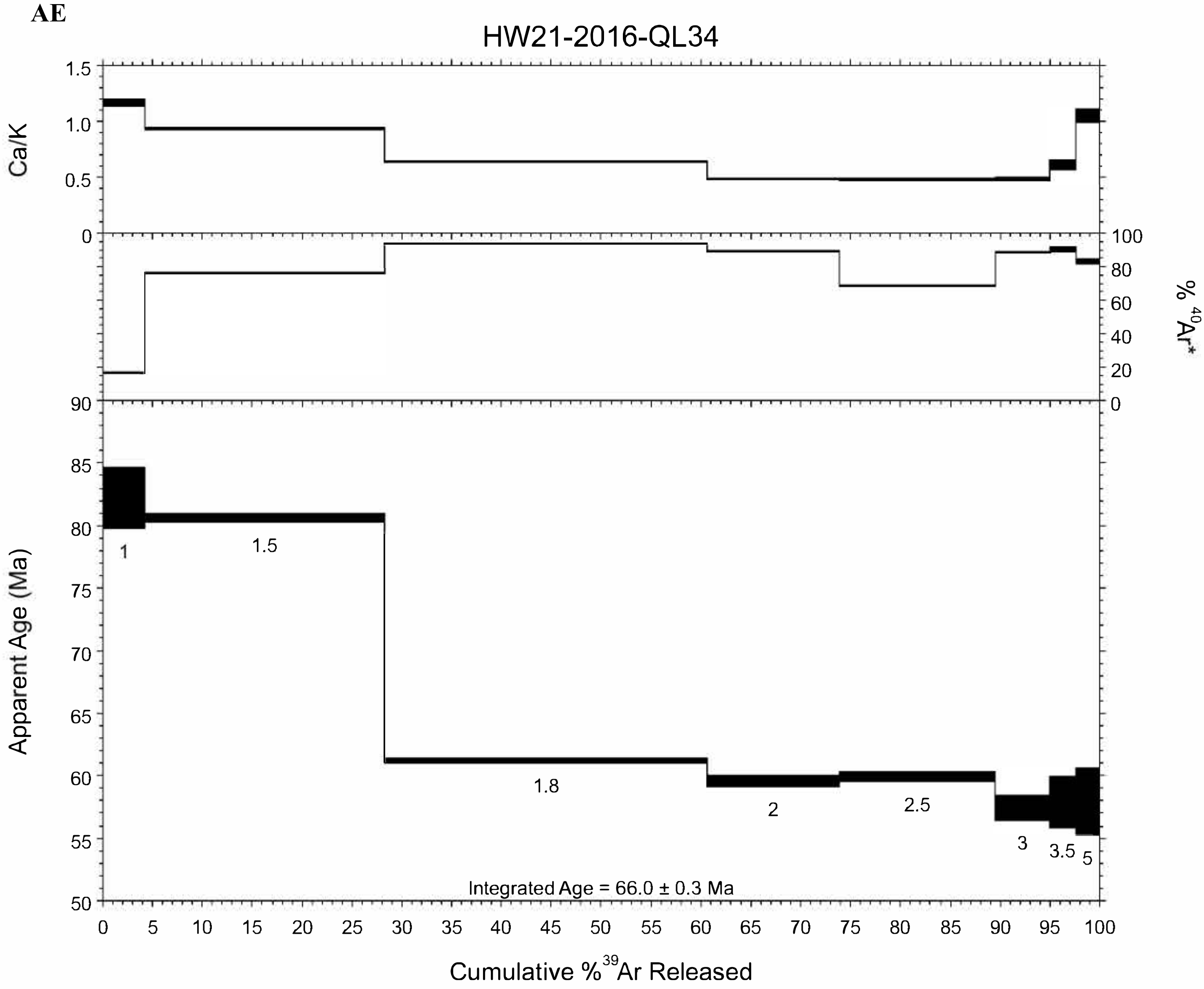

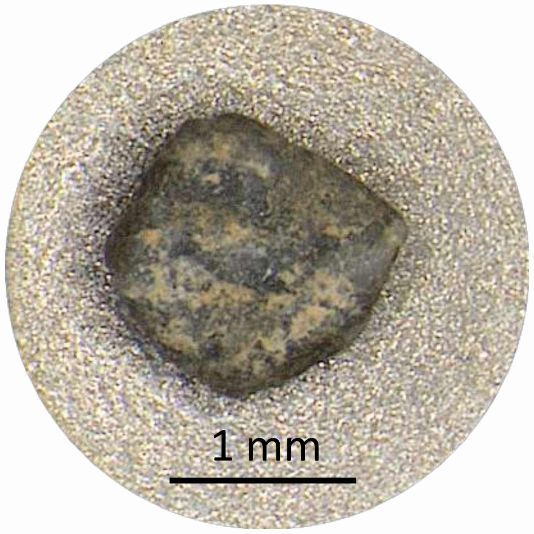

Fig. S3 continued.

AF

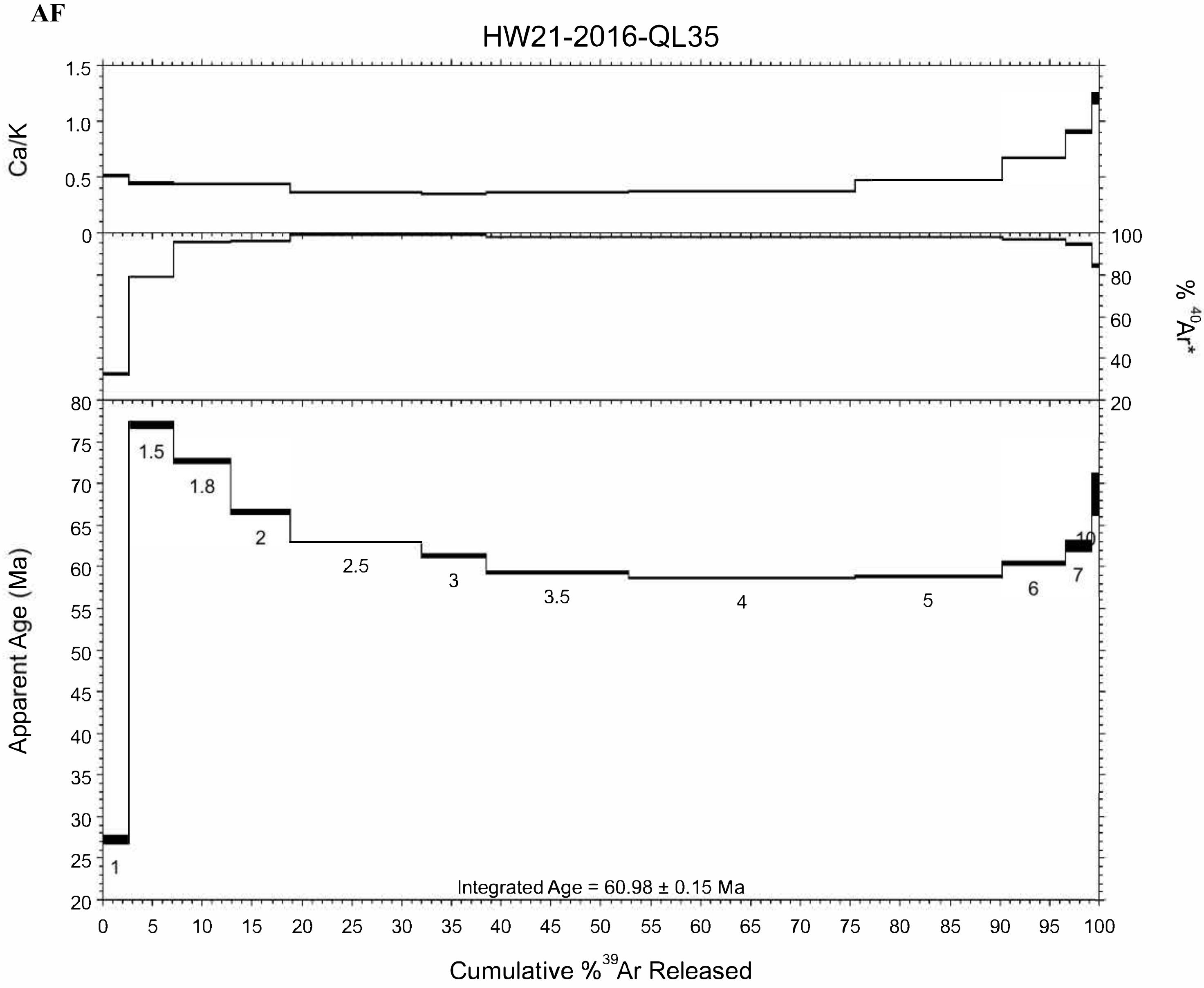

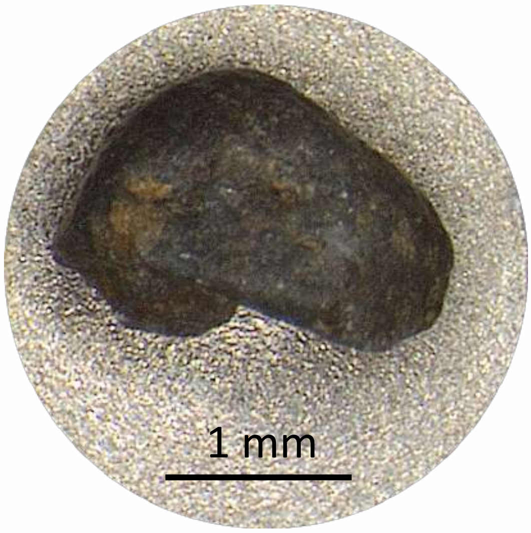

Fig. S3 continued.

AG

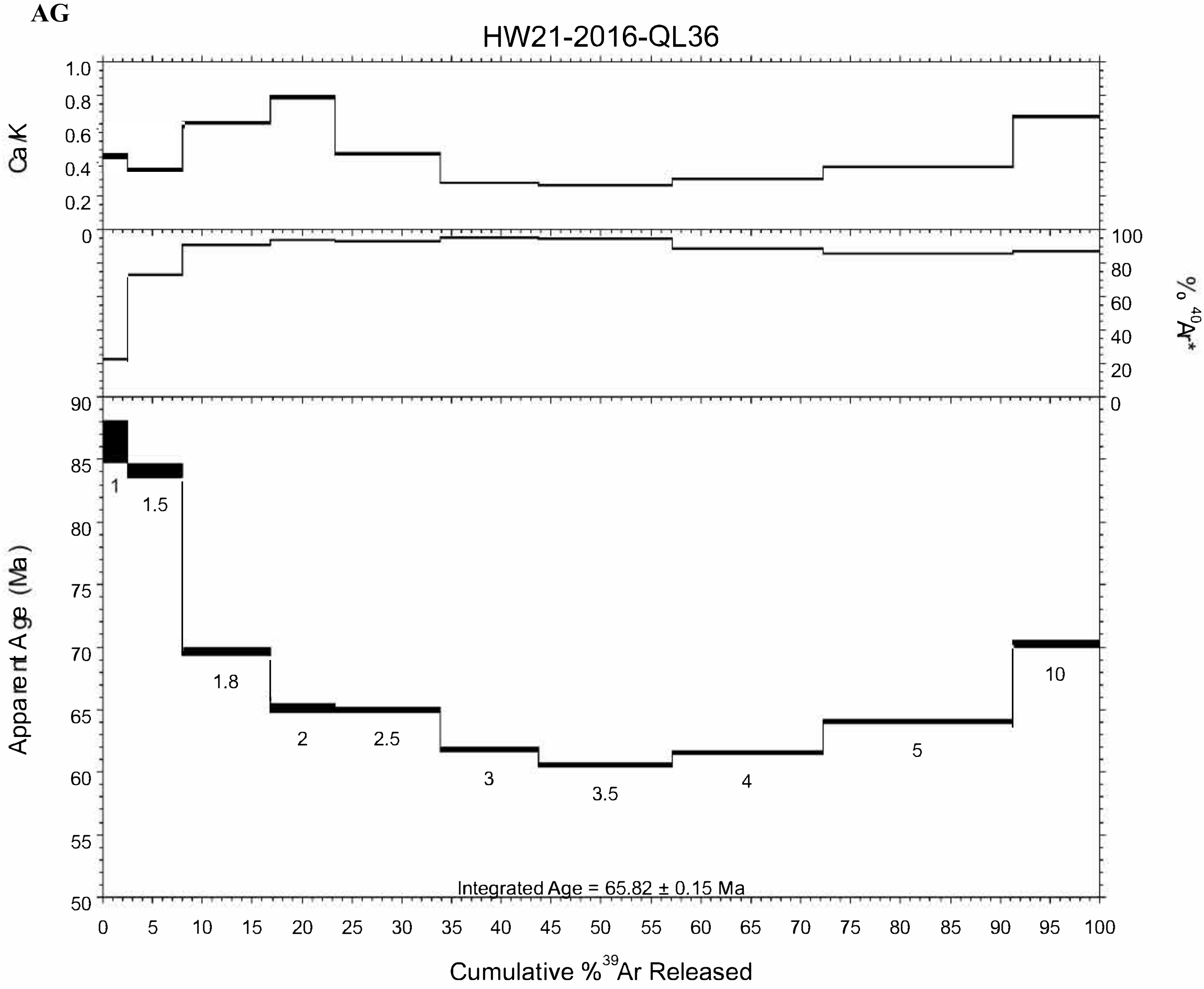

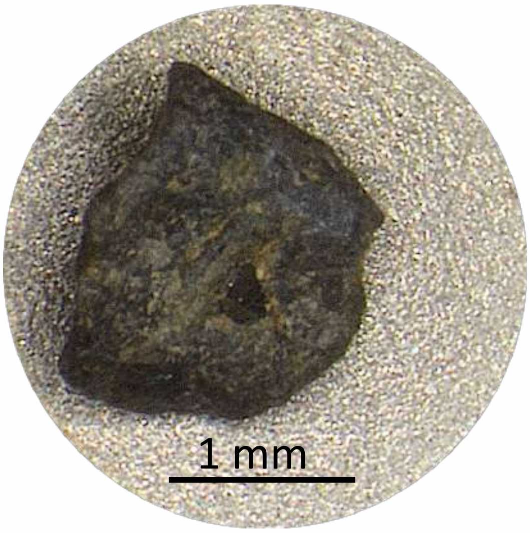

Fig. S3 continued.

AH

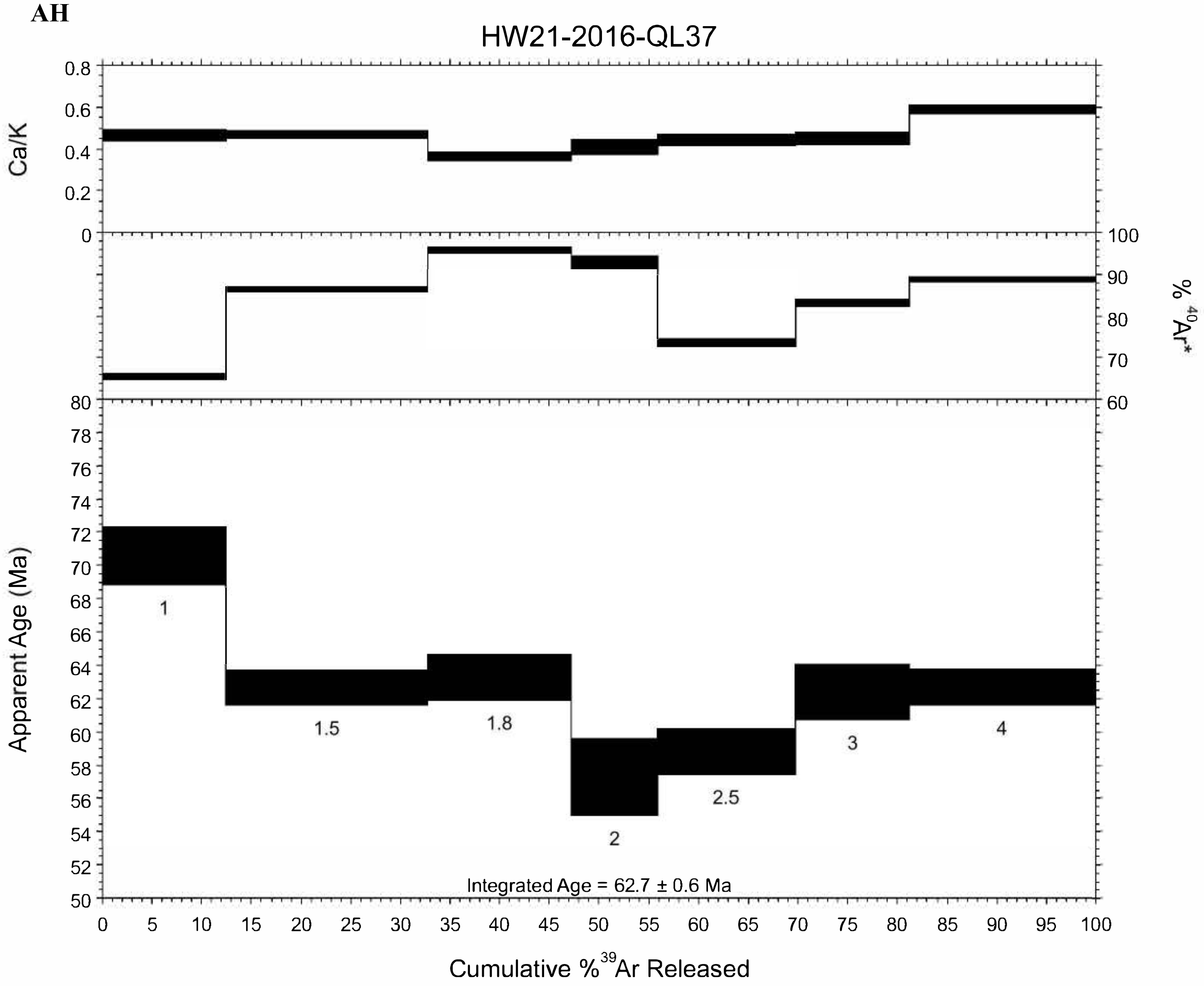

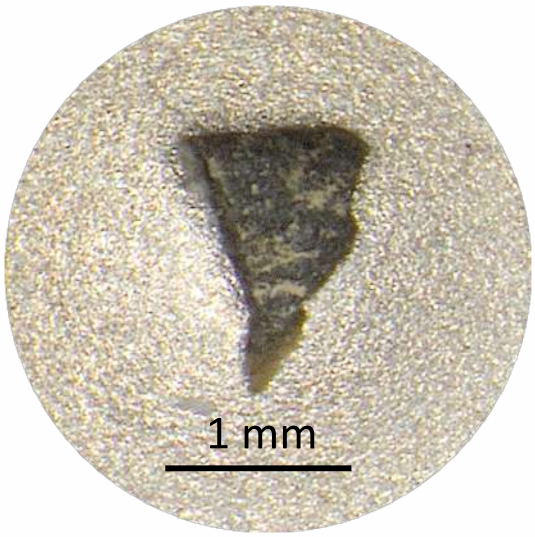

Fig. S3 continued.

AI

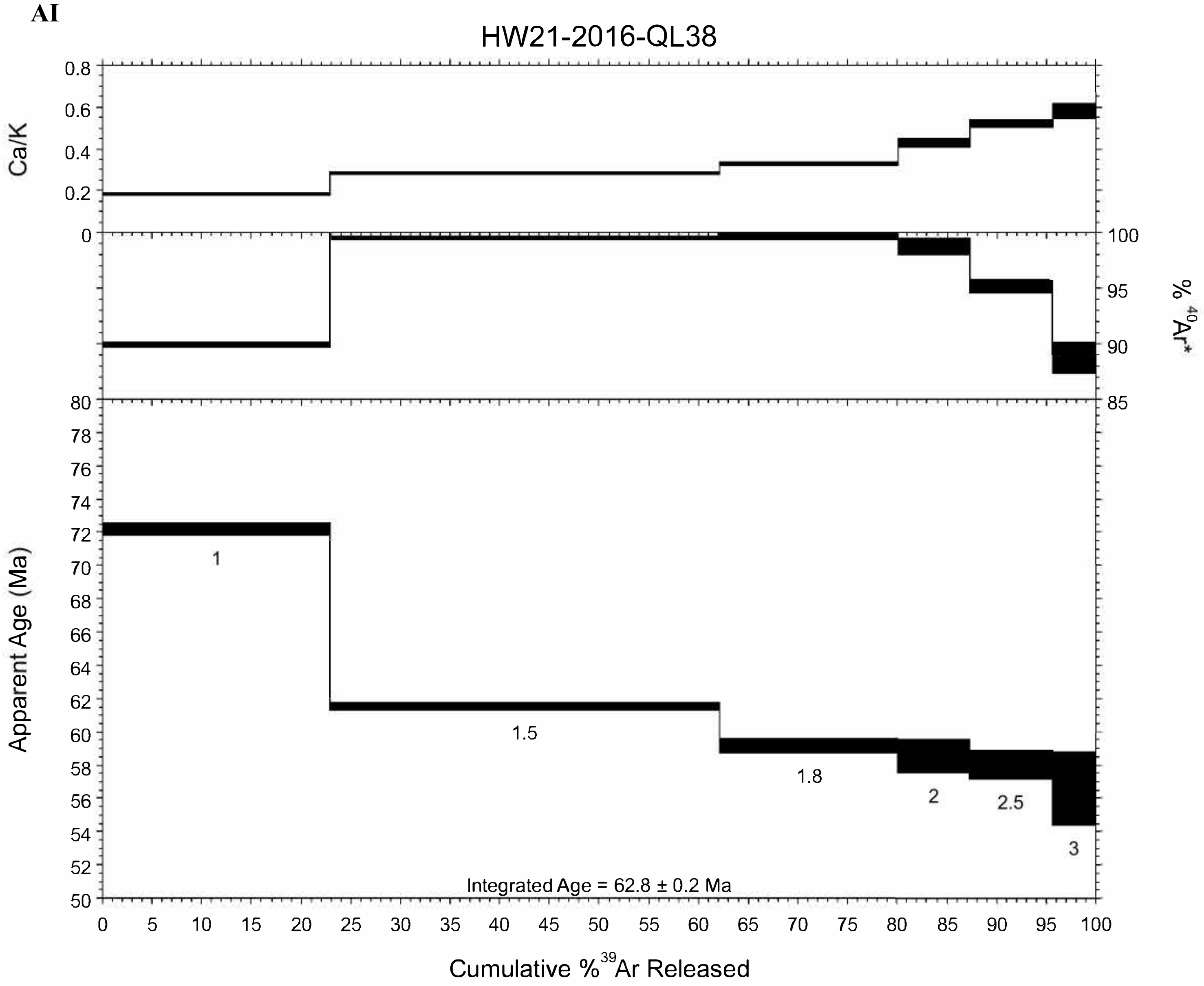

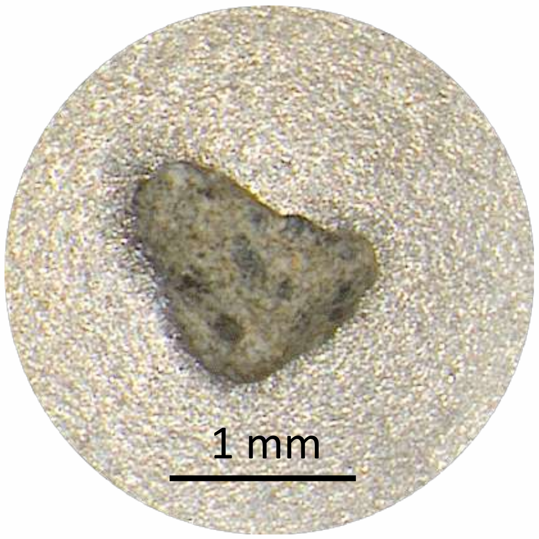

Fig. S3 continued.

AJ

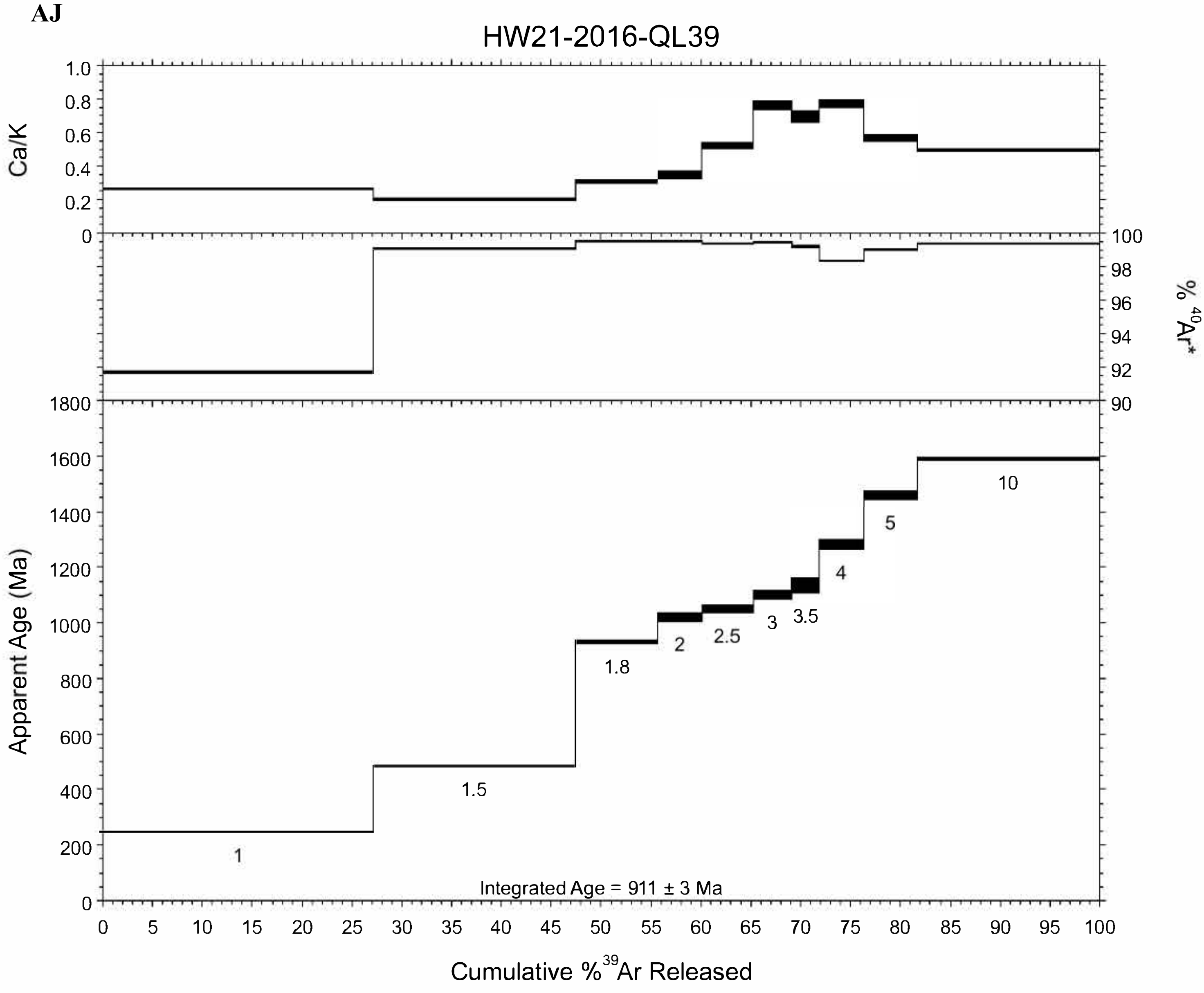

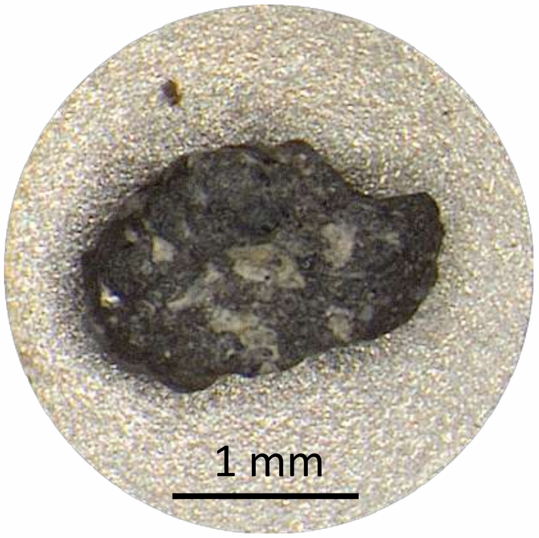

Fig. S3 continued.

AK

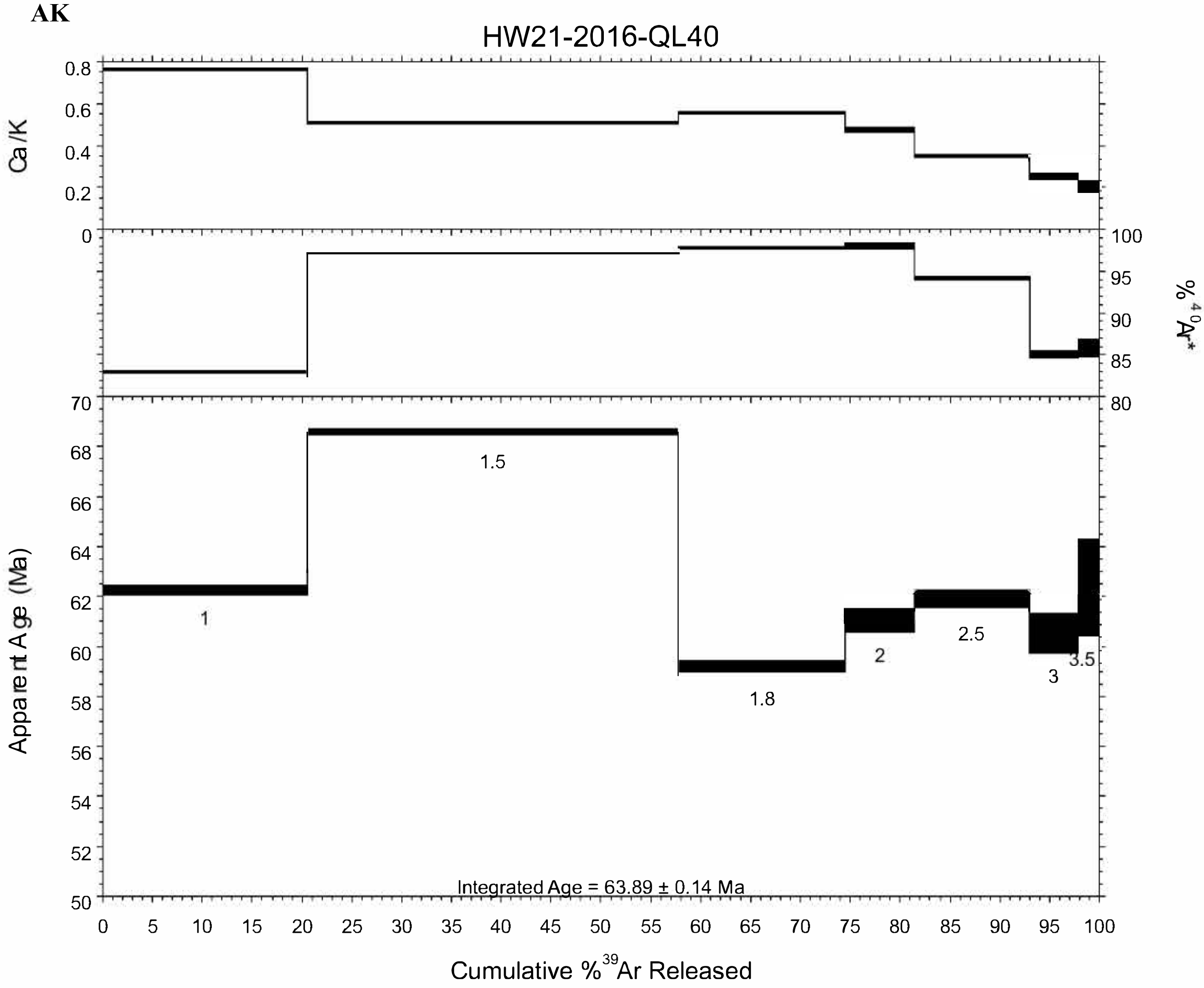

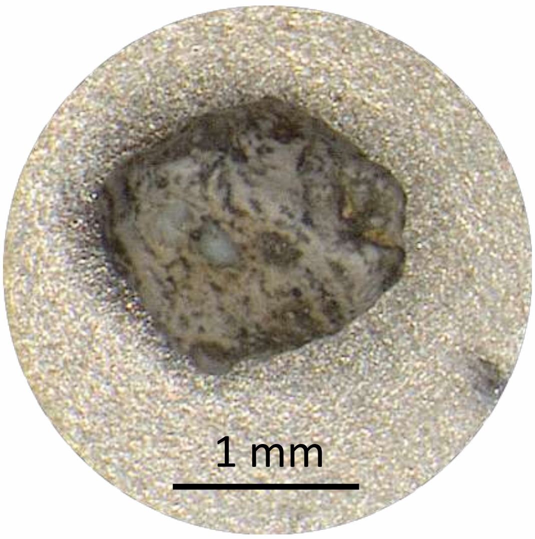

Fig. S3 continued.

AL

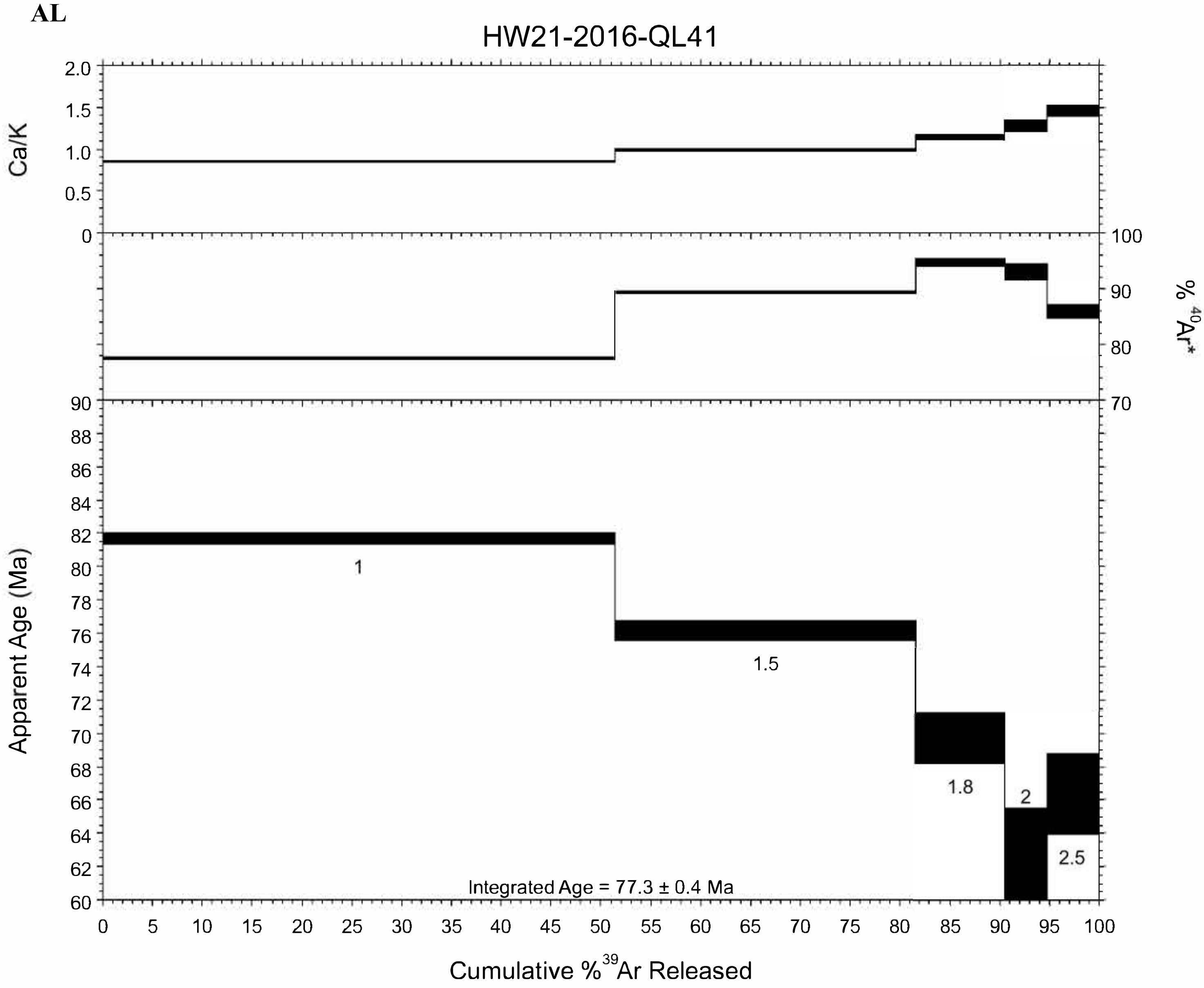

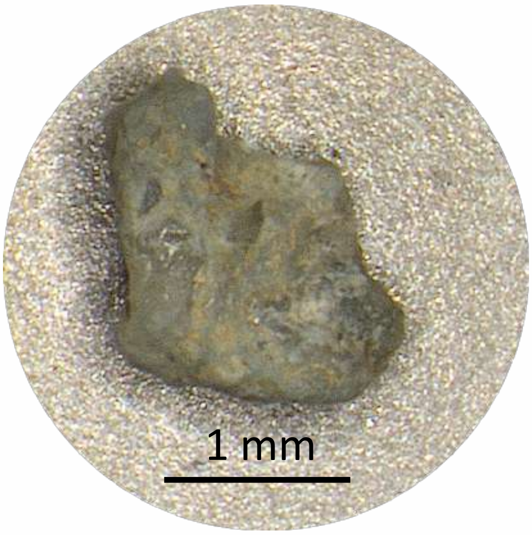

Fig. S3 continued.

AM

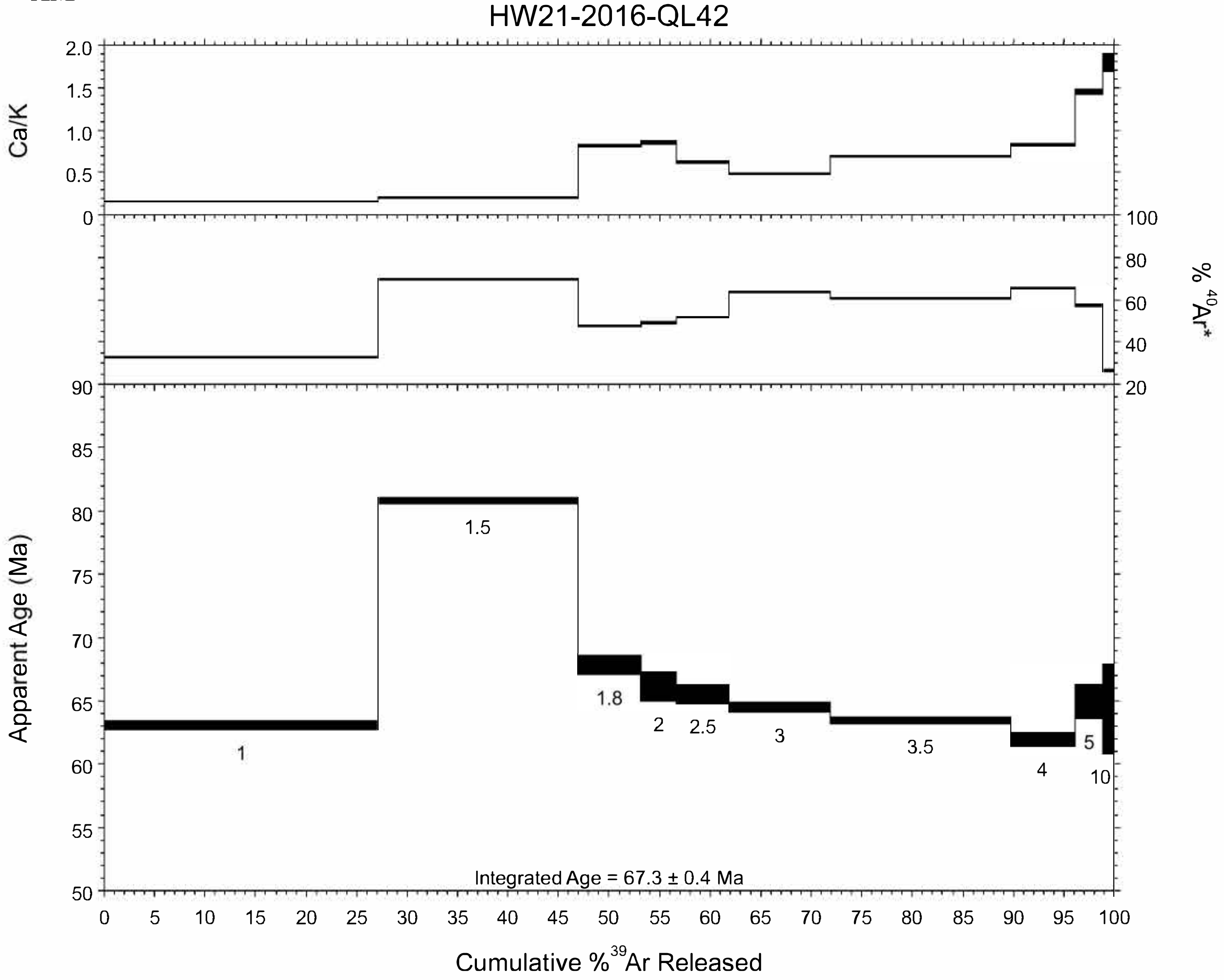

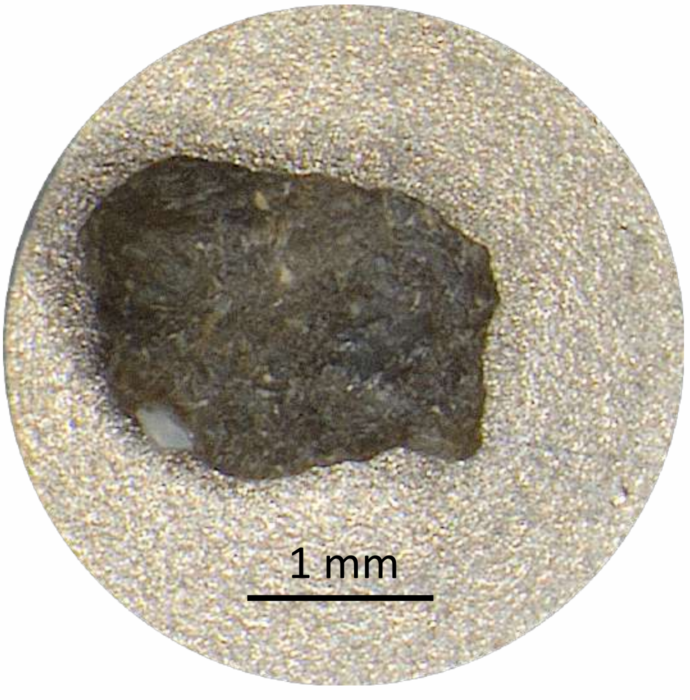

Fig. S3 continued.

AN

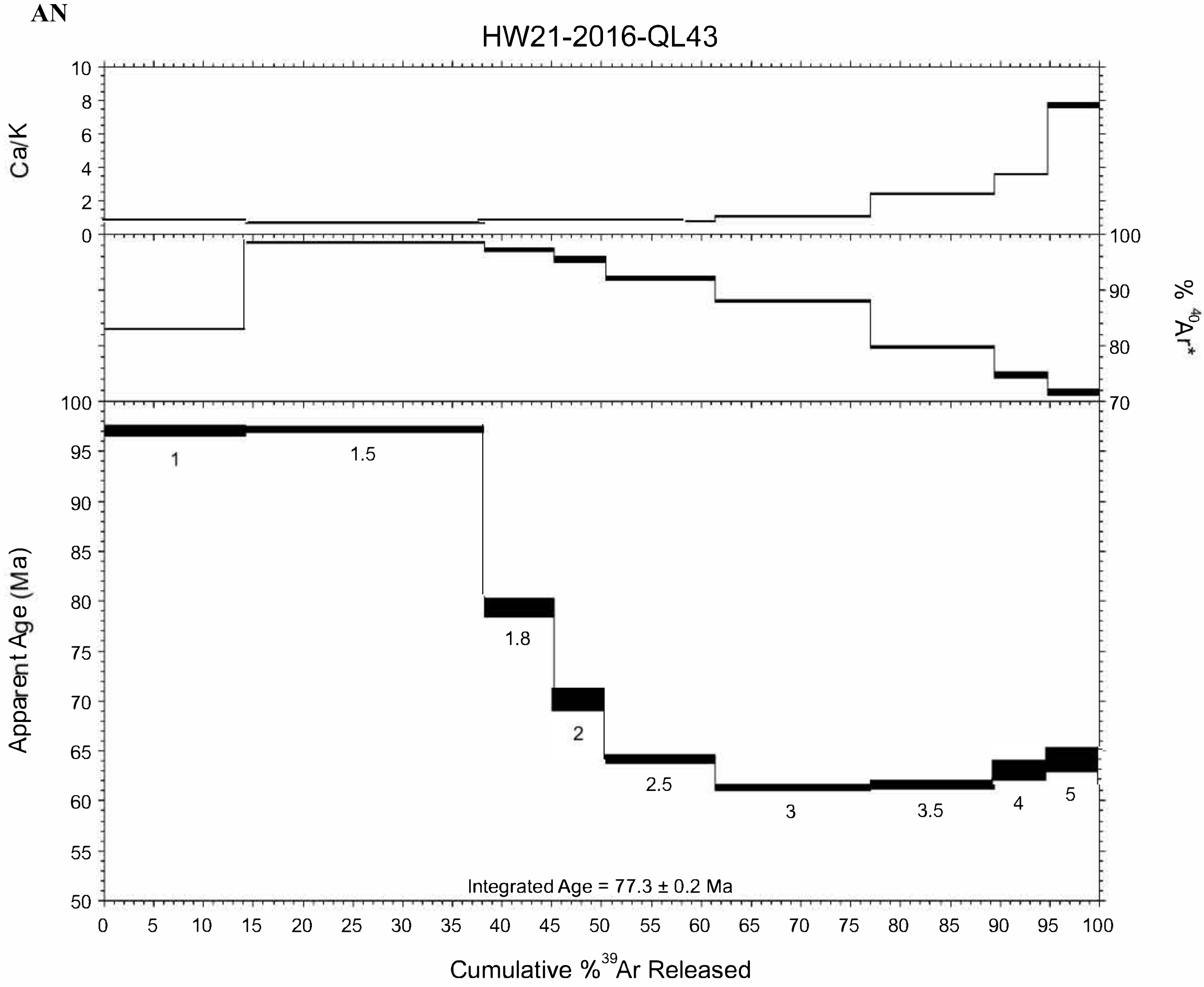

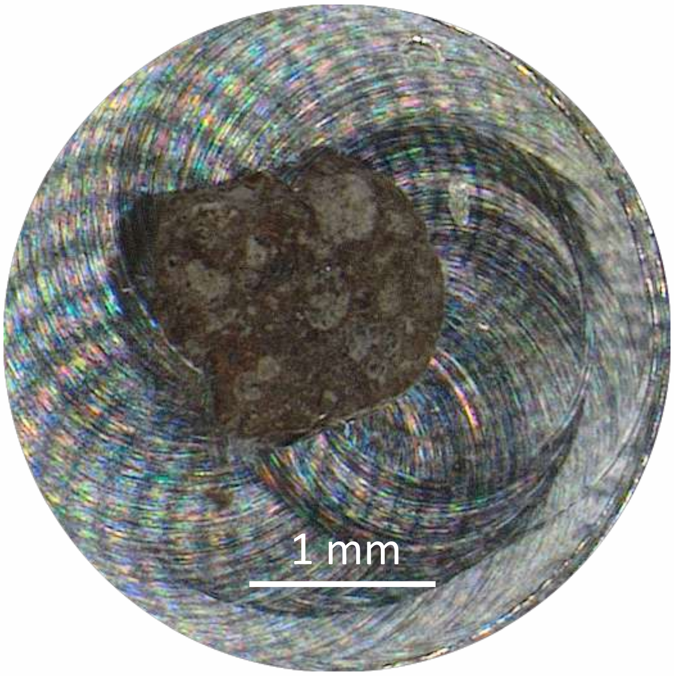

Fig. S3 continued.

AO

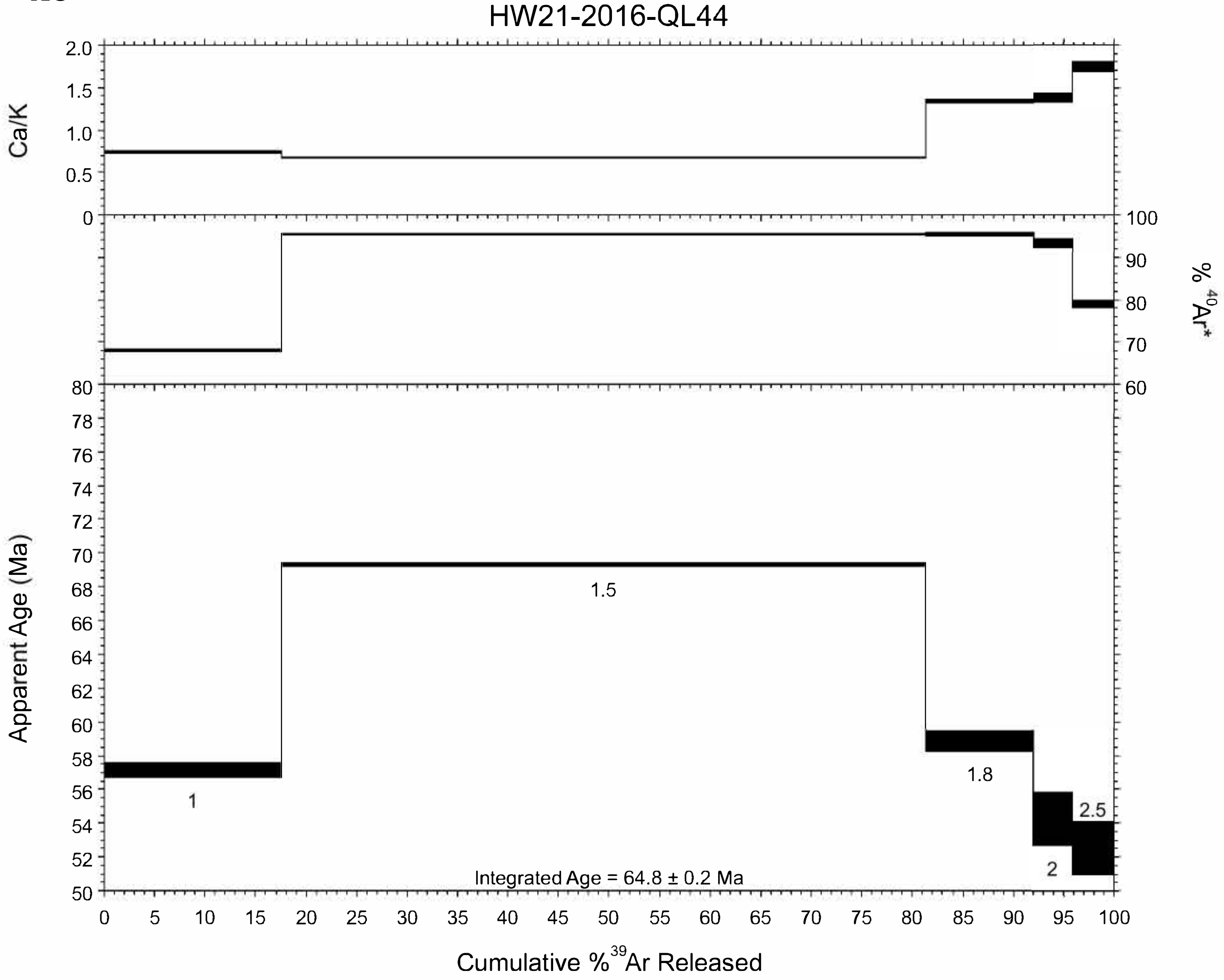

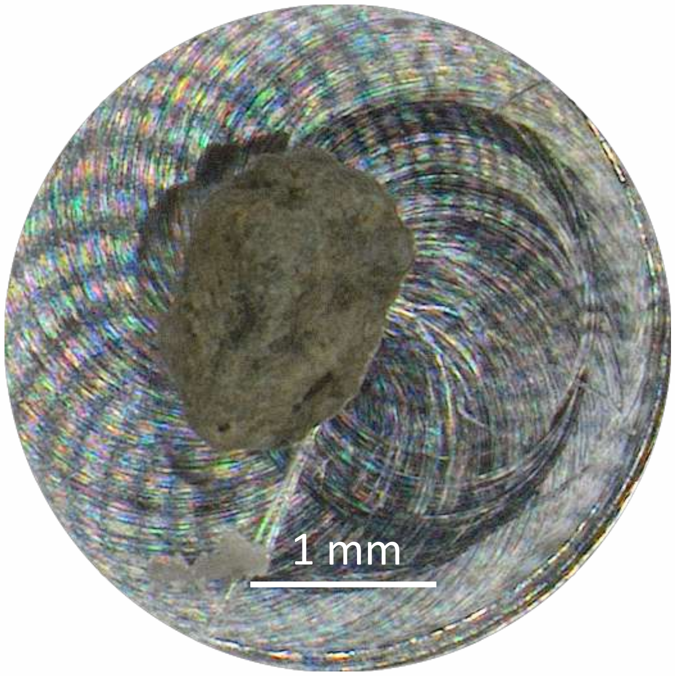

Fig. S3 continued.

AP

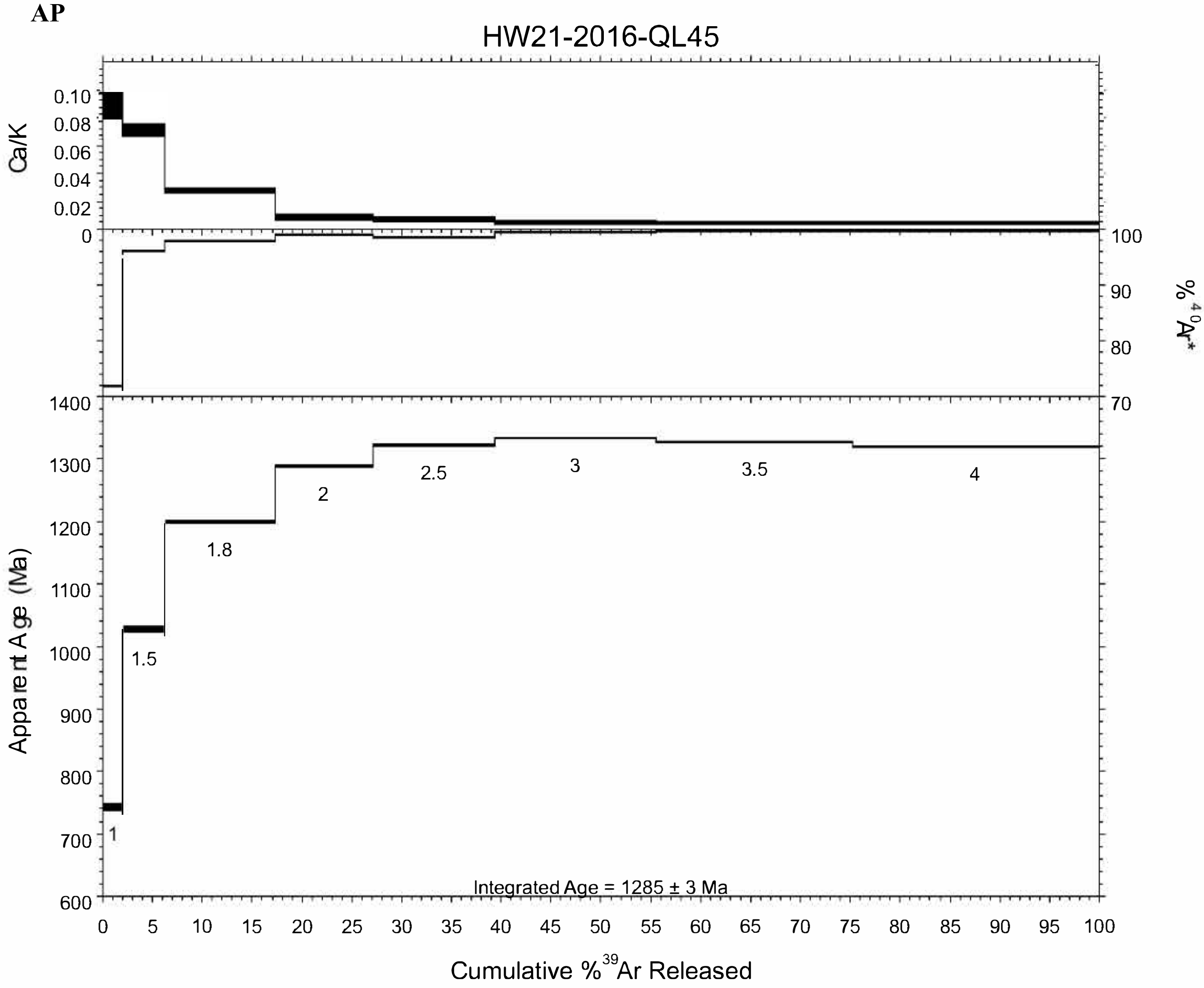

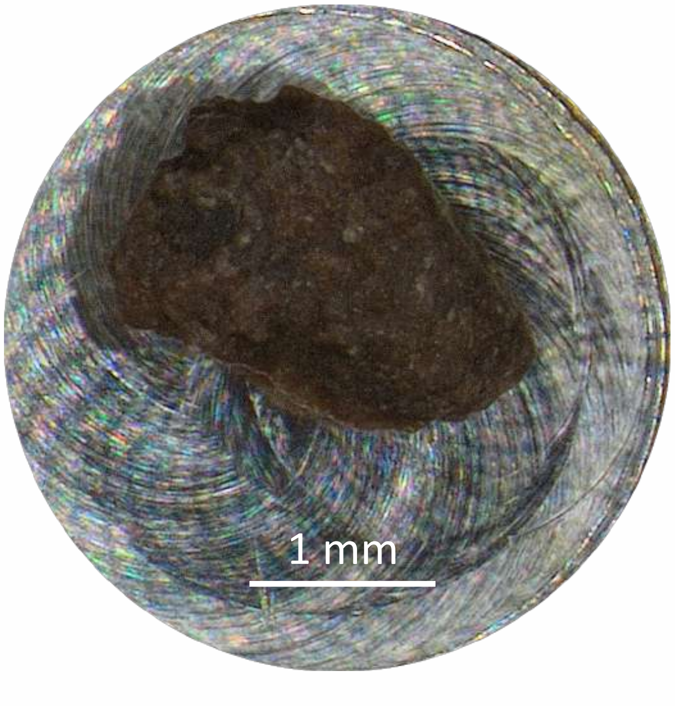

Fig. S3 continued.

AQ

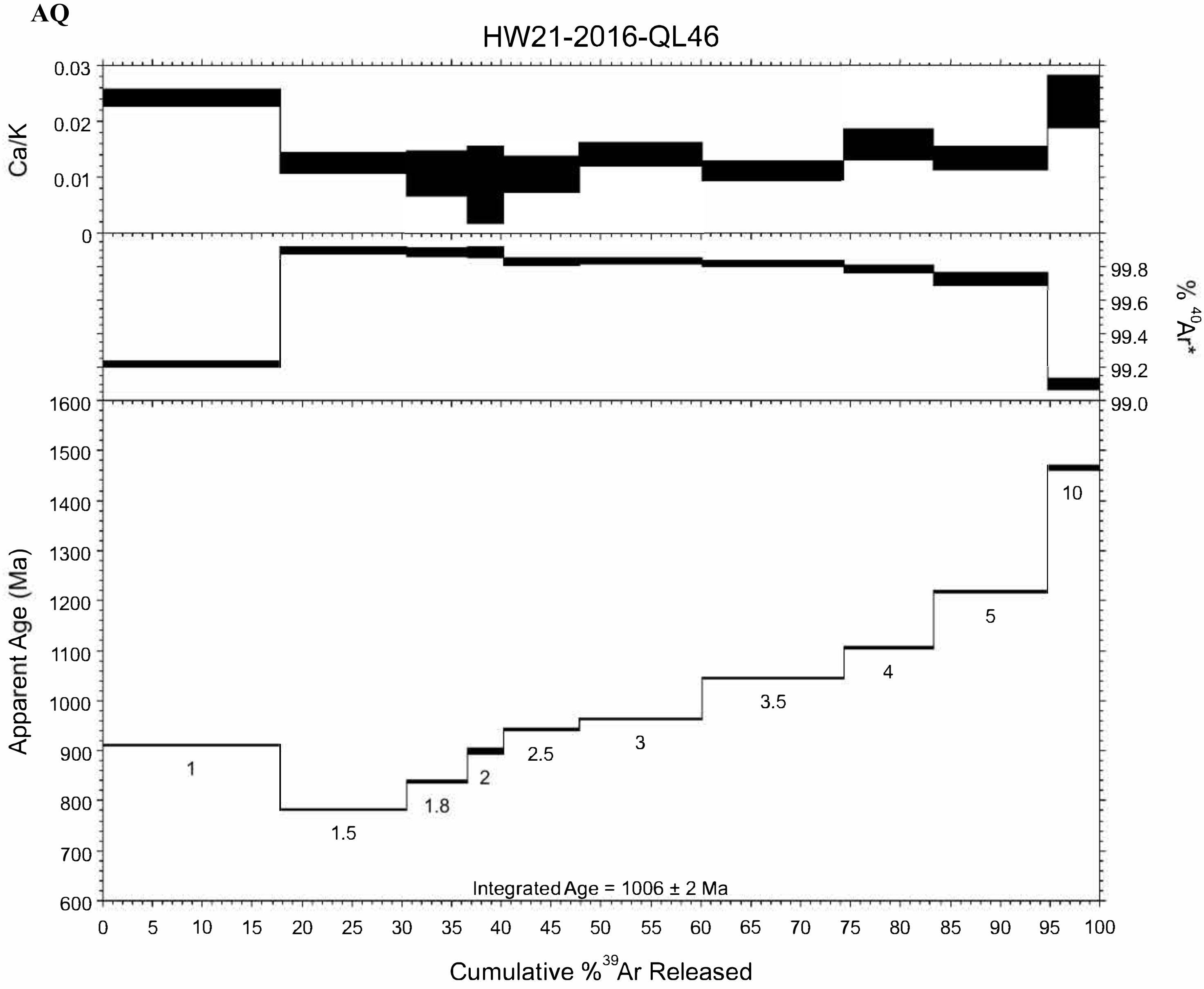

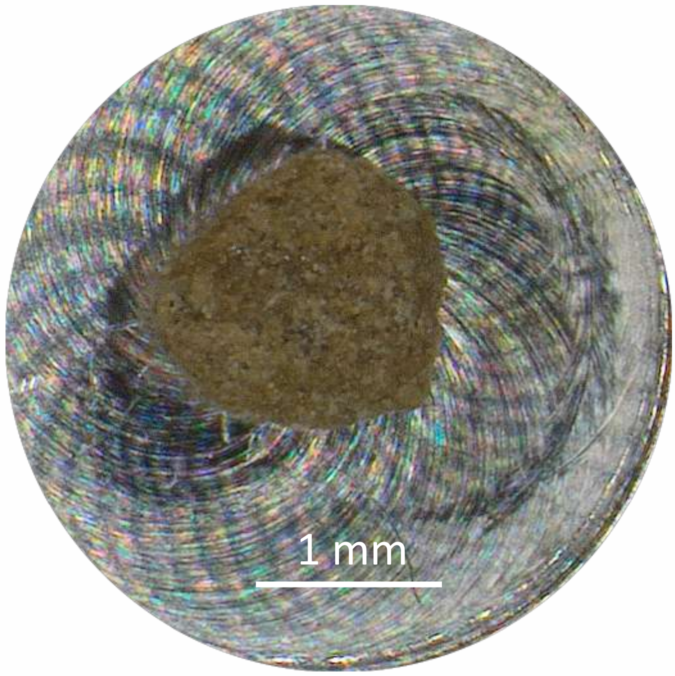

Fig. S3 continued.

AR

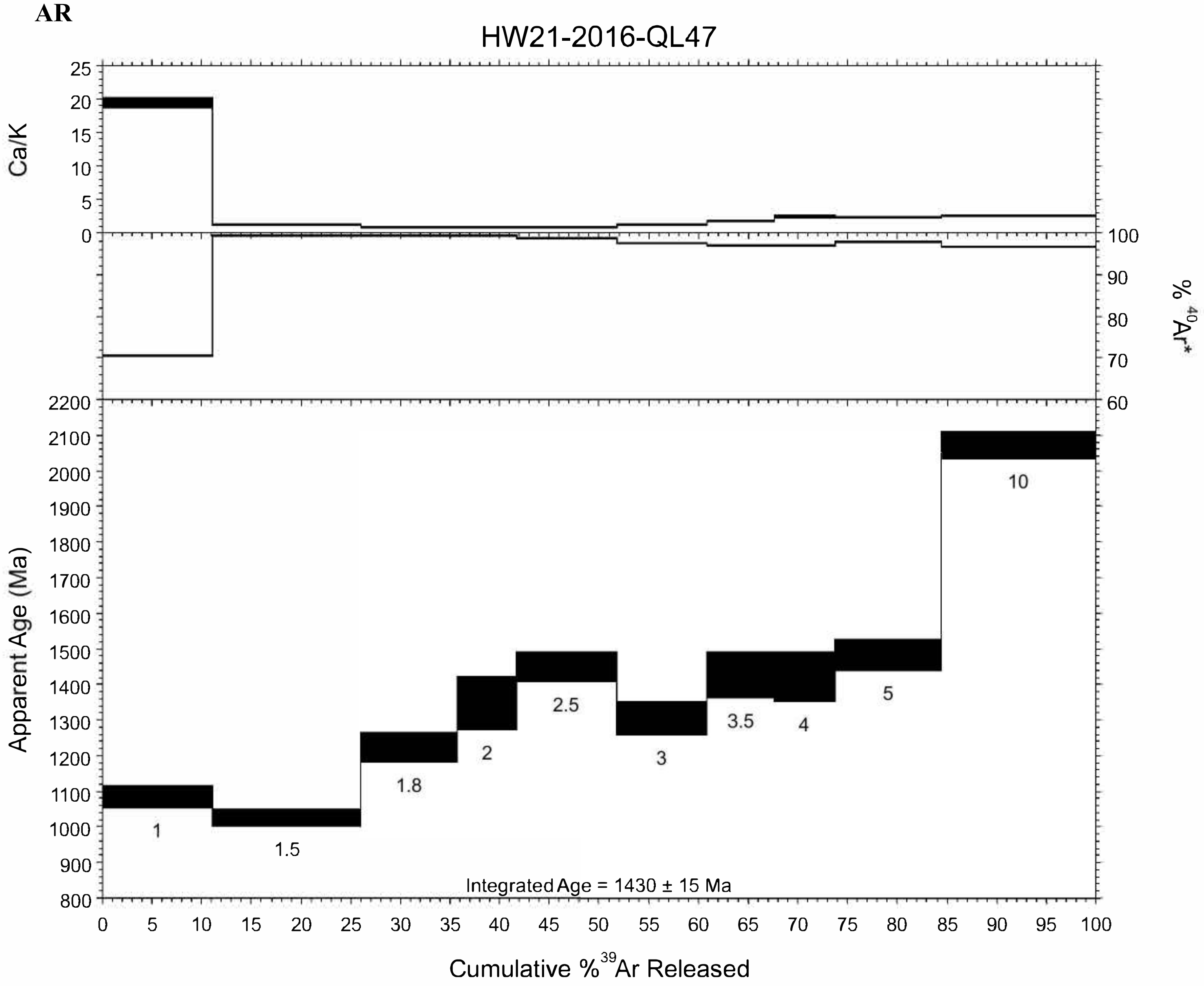

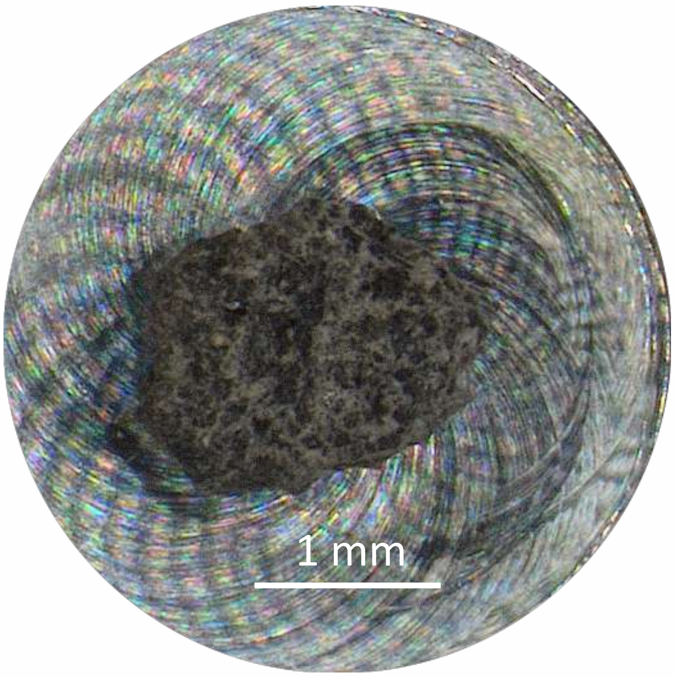

Fig. S3 continued.

AS

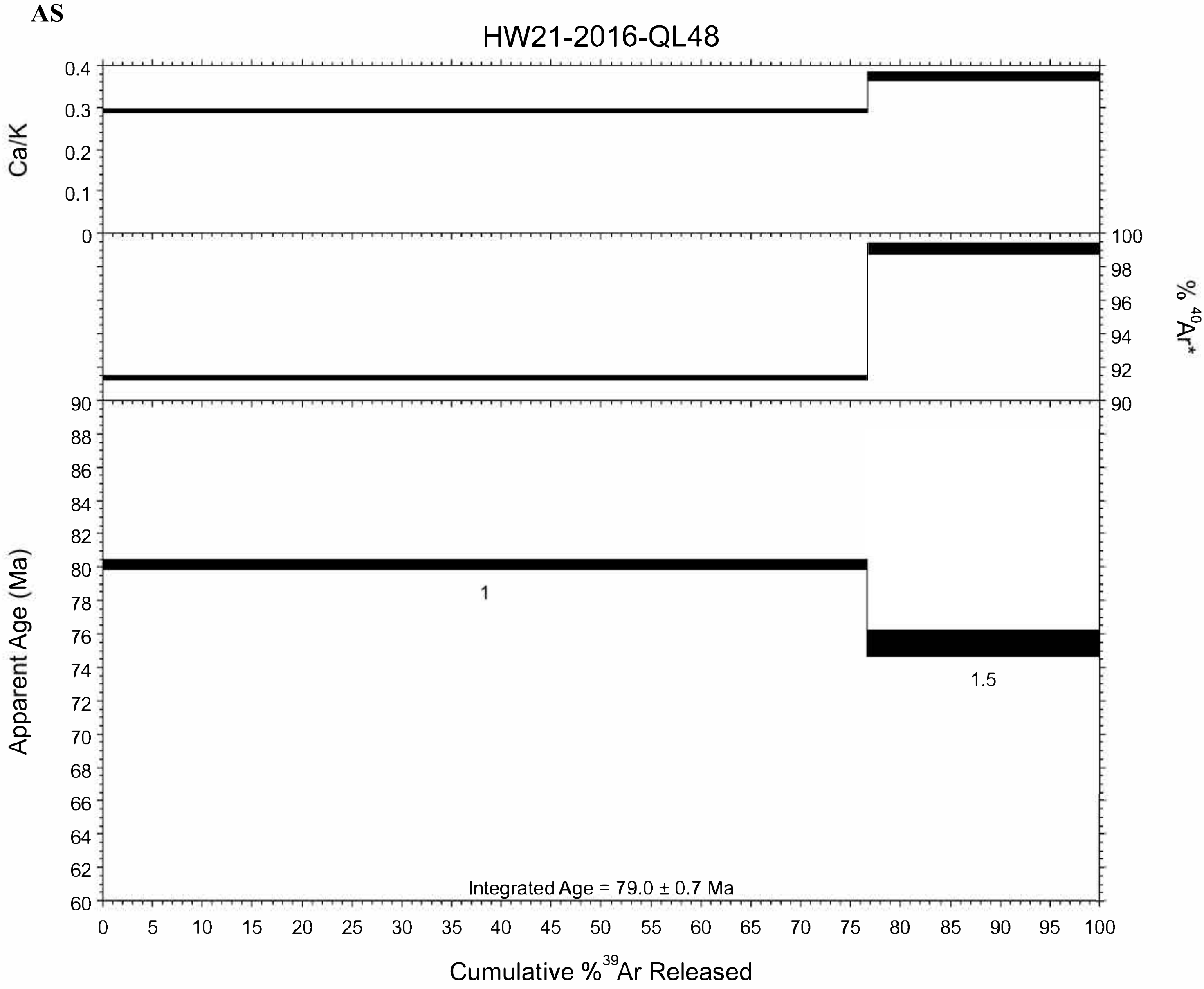

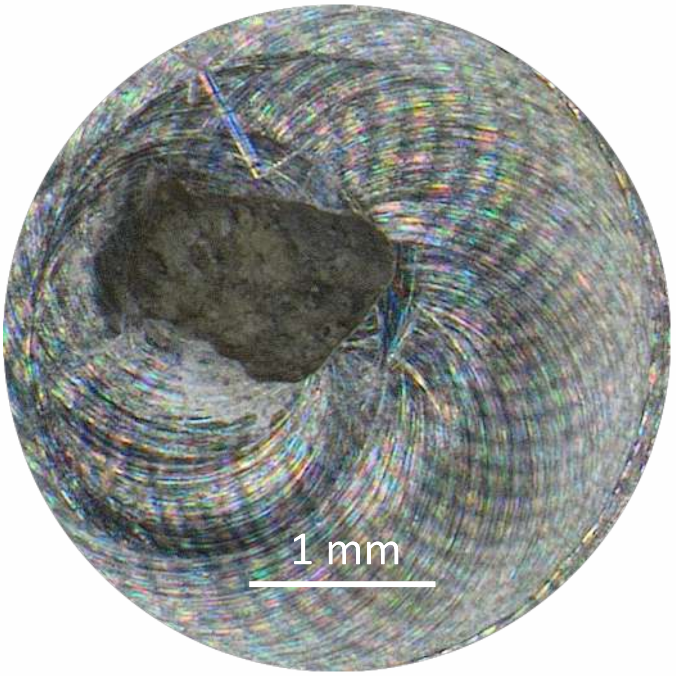

Fig. S3 continued.

AT

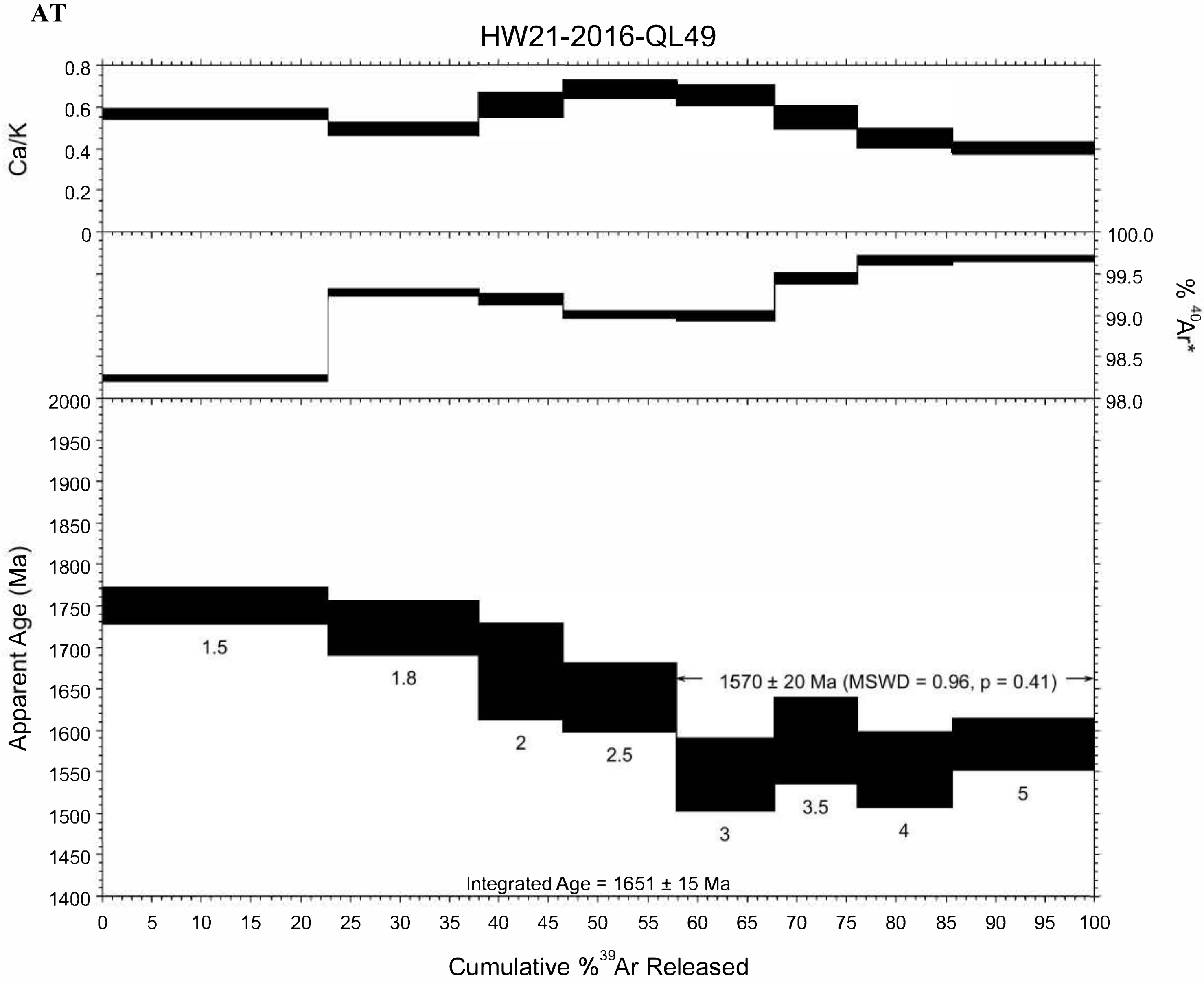

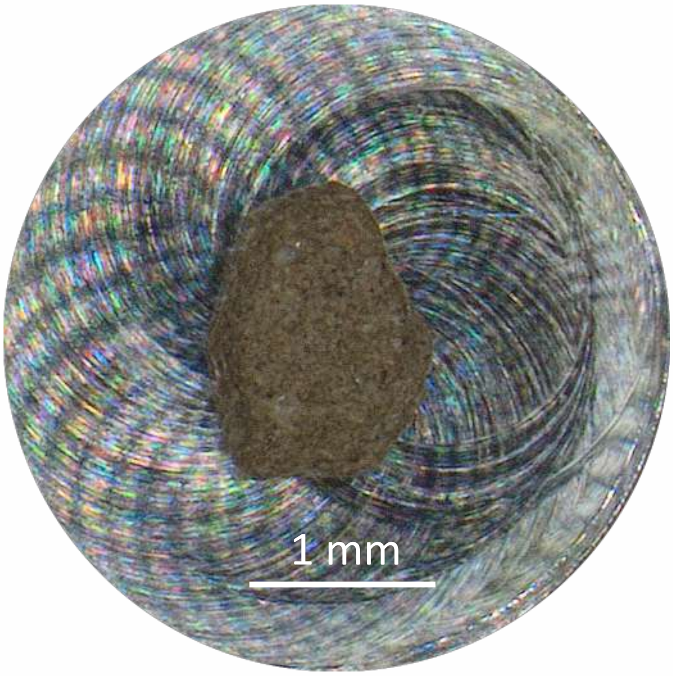

Fig. S3 continued.

AU

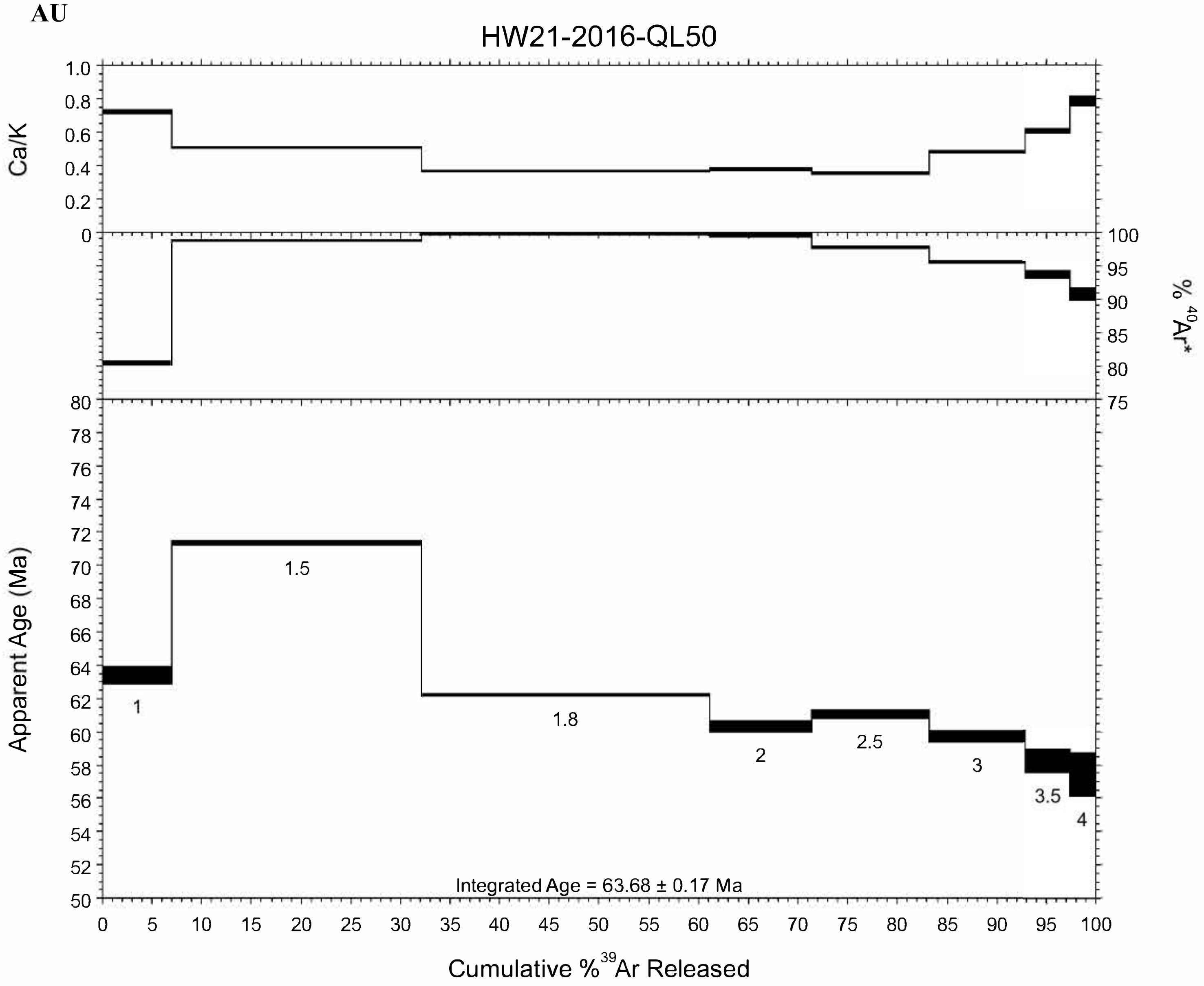

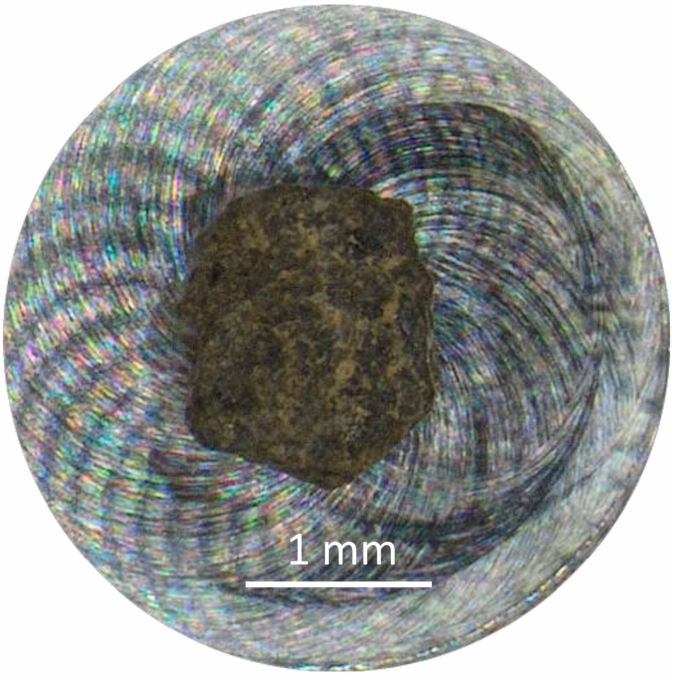

Fig. S3 continued.

AV

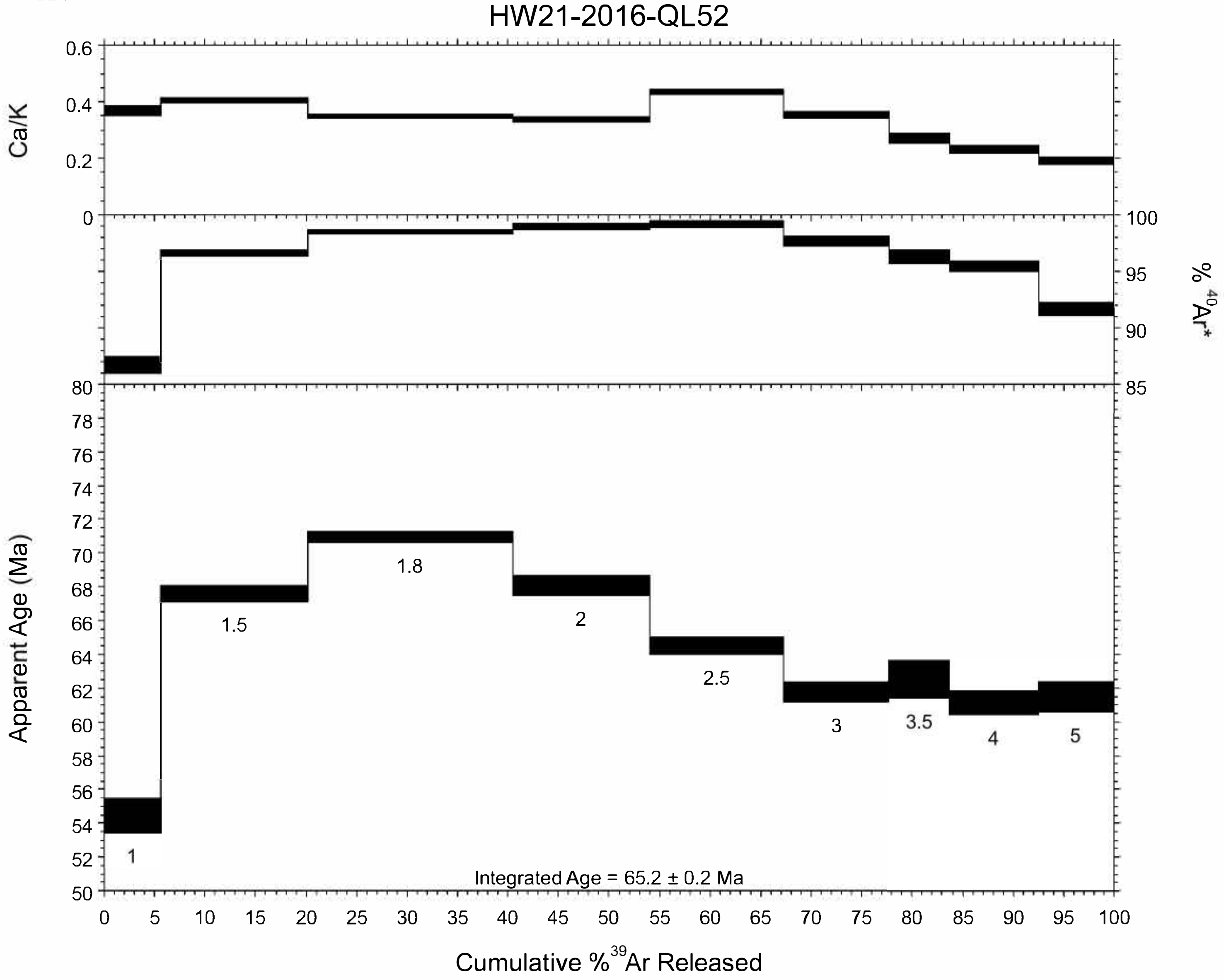

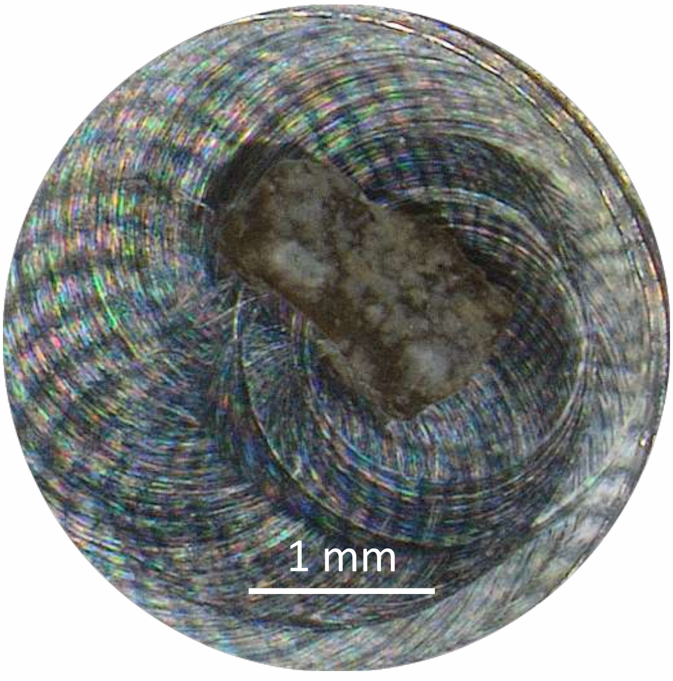

Fig. S3 continued.

AW

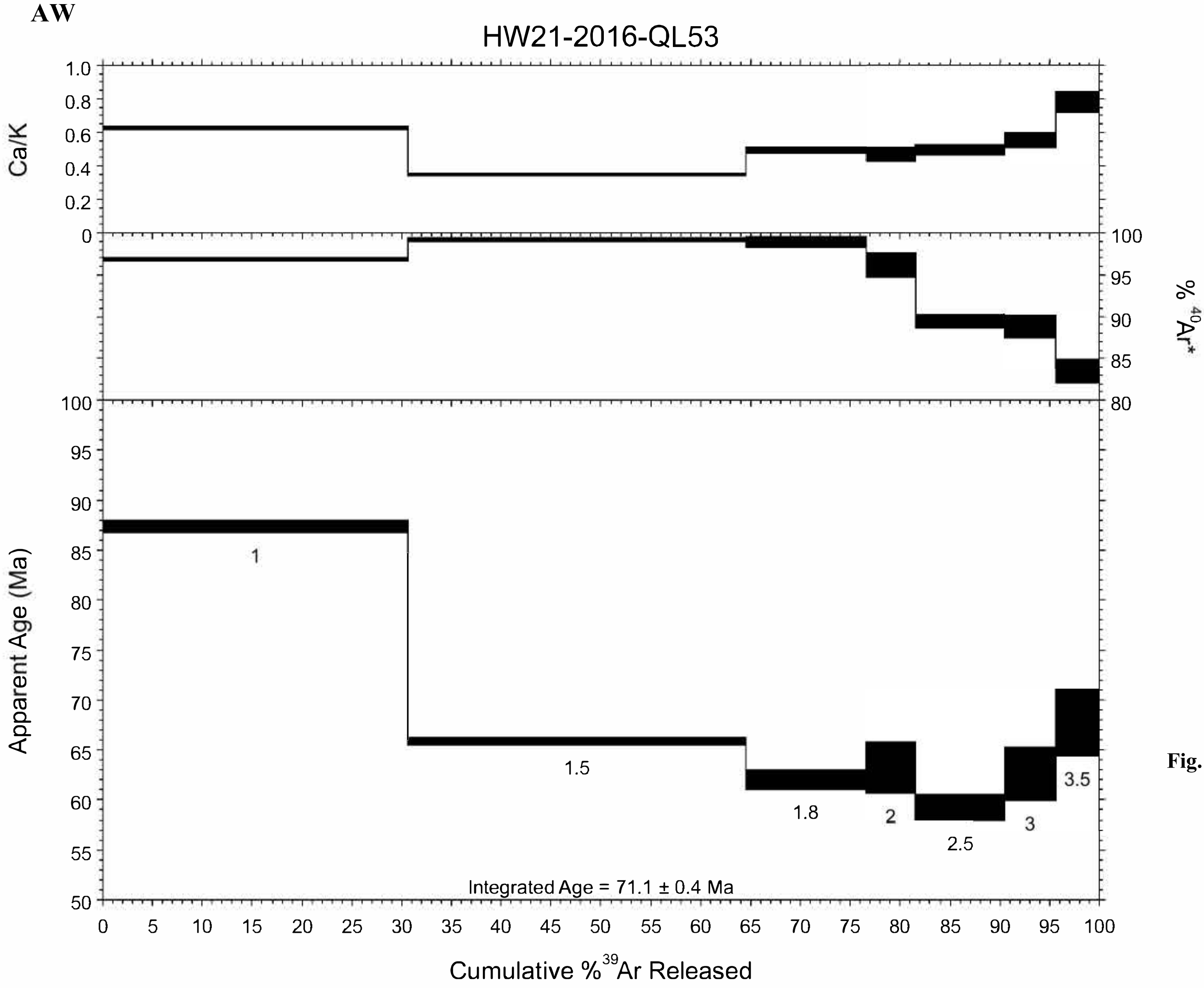

Fig. S3 co

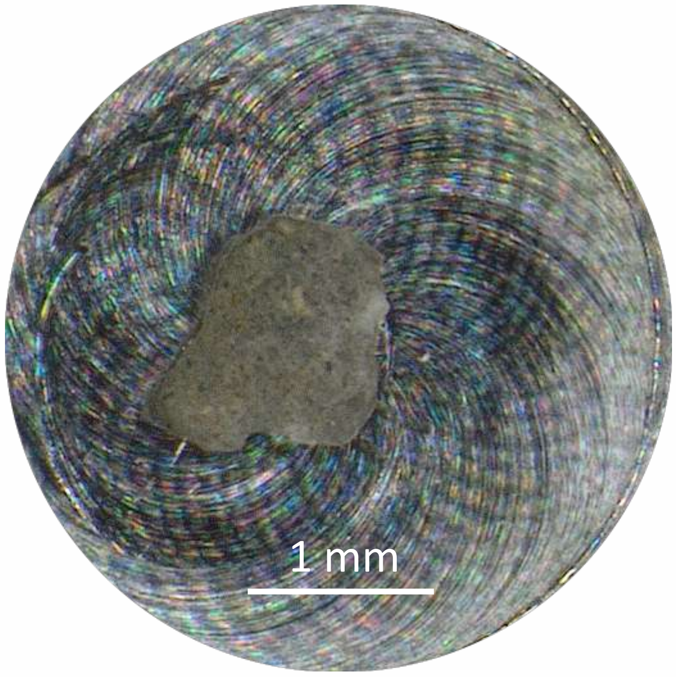

Fig. S3 continued.

AX

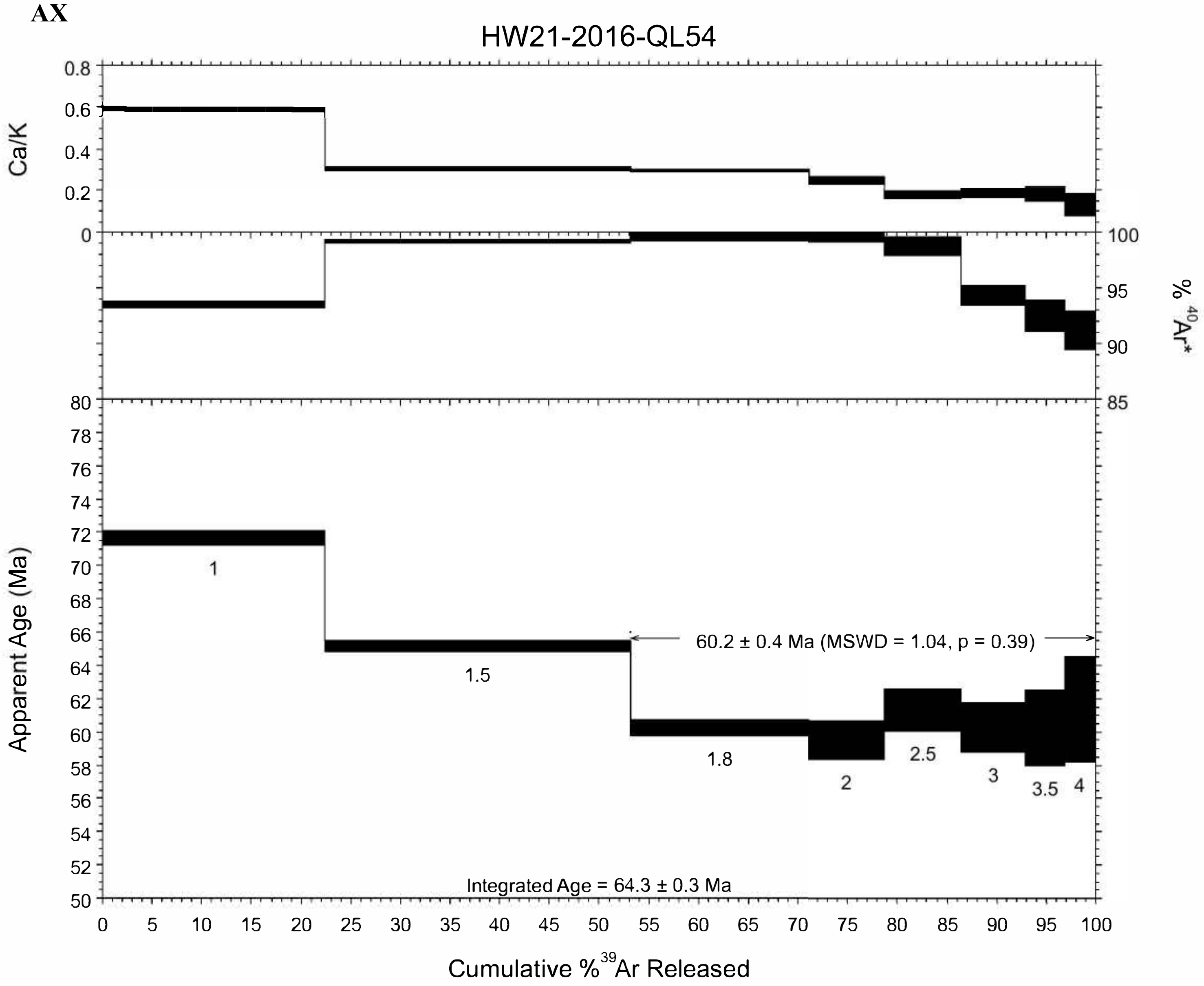

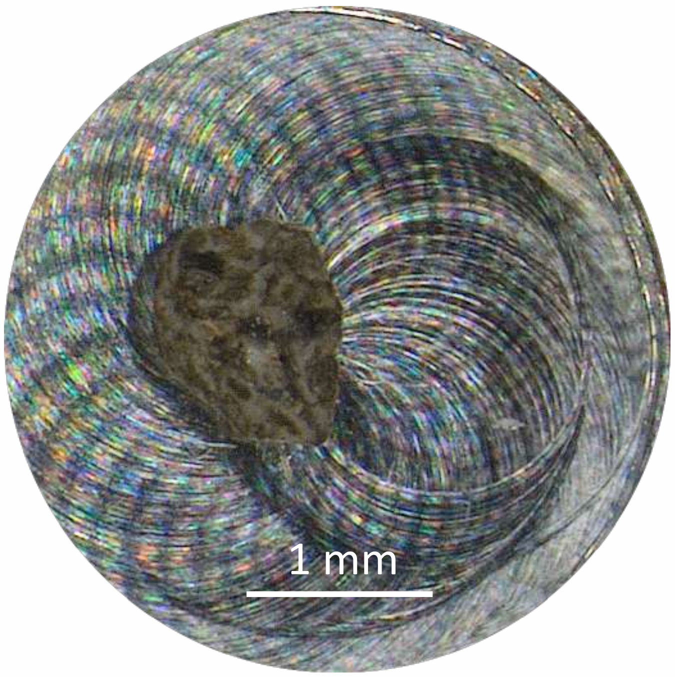

Fig. S3 continued.

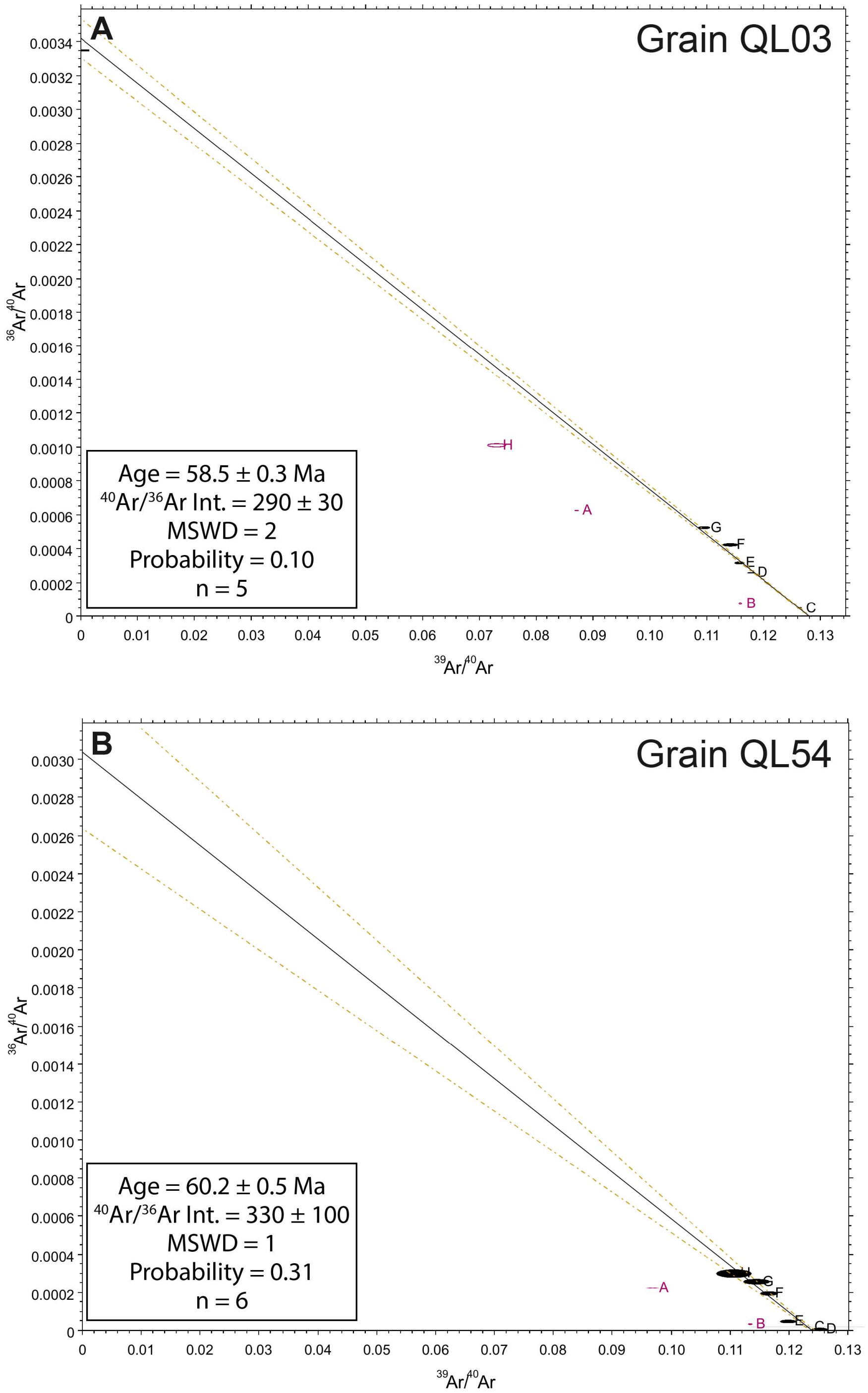

Fig. S4. Inverse isochron plots of $^{36}Ar/^{40}Ar$ vs. $^{39}Ar/^{40}Ar$ for the two grains that gave mini-plateau ages, QL03 and QL54. Isochron ages and $^{40}Ar/^{36}Ar$ are presented at 2σ. Isochron error envelope is indicated. Letters represent sequential heating steps. Isochron ages are calculated from the same heating steps (black ellipses) used to calculate the plateau ages (Fig. 4A, fig. S3). Note that the inverse isochron ages are identical to the mini-plateau ages and have $^{40}Ar/^{36}Ar$ intercepts that are within error of the atmospheric value. Int. – intercept; MSWD – mean square of weighted deviates.

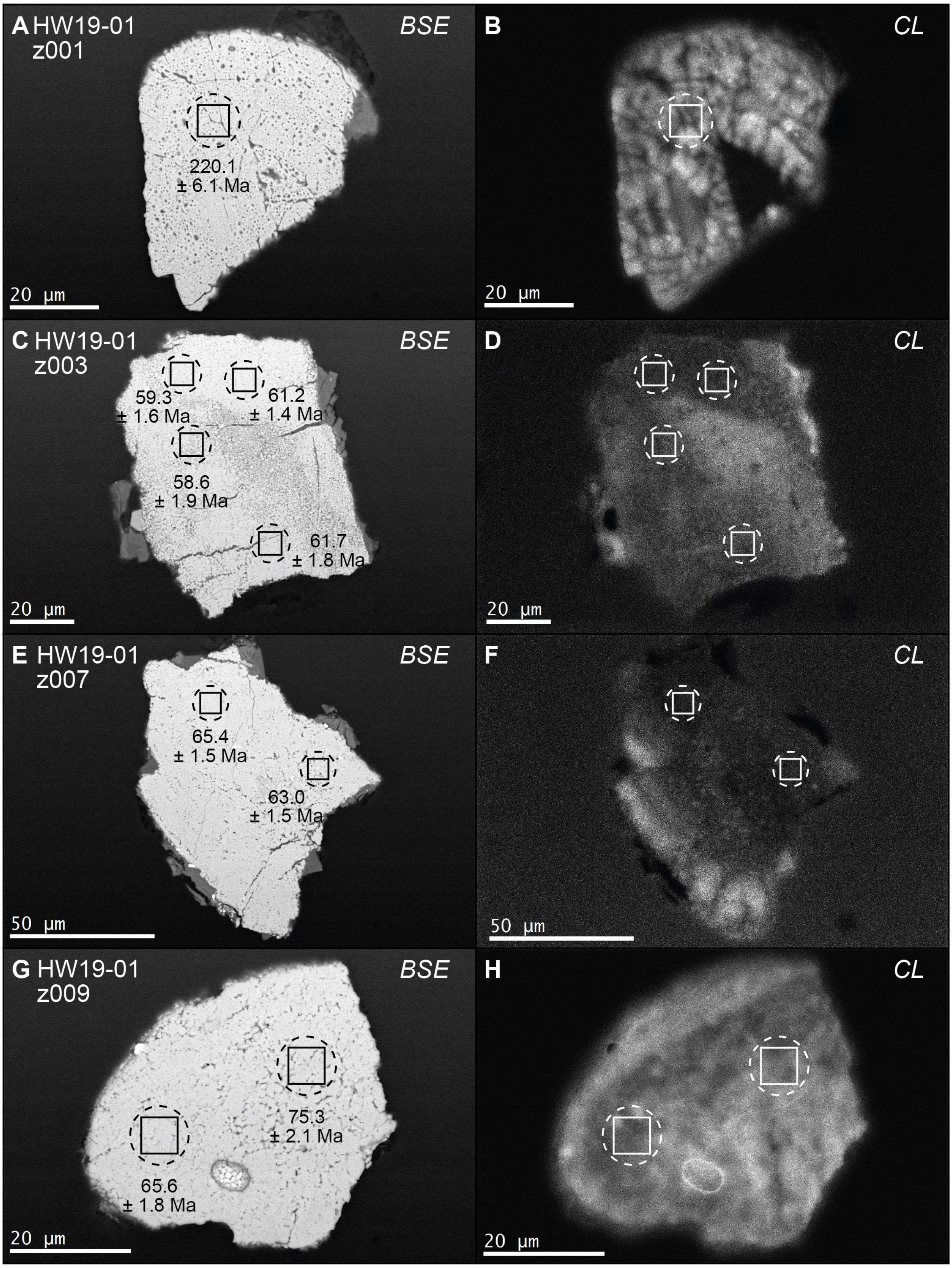
A HW19-01
z001
BSE
220.1
± 6.1 Ma
20 μm
B
CL
20 μm
C HW19-01
z003
BSE
59.3
± 1.6 Ma
61.2
± 1.4 Ma
58.6
± 1.9 Ma
61.7
± 1.8 Ma
20 μm
D
CL
20 μm
E HW19-01
z007
BSE
65.4
± 1.5 Ma
63.0
± 1.5 Ma
50 μm
F
CL
50 μm
G HW19-01
z009
BSE
75.3
± 2.1 Ma
65.6
± 1.8 Ma
20 μm
H
CL
20 μm

Fig. S5. Backscattered electron (BSE) and cathodoluminescence (CL) imaging of zircon grains from the Hiawatha impact structure. Dashed circle represents the ~10 μm-diameter U–Pb analysis pit and the square represents the approximately 6 × 6 μm^2 area that was sampled for U–Pb geochronology after the secondary ion beam passed through the field aperture (see methods). Apparent ages less than 1200 Ma are $^{206}Pb/^{238}U$ ages; those above 1200 Ma are $^{207}Pb/^{206}Pb$ ages. Uncertainties are 2σ.

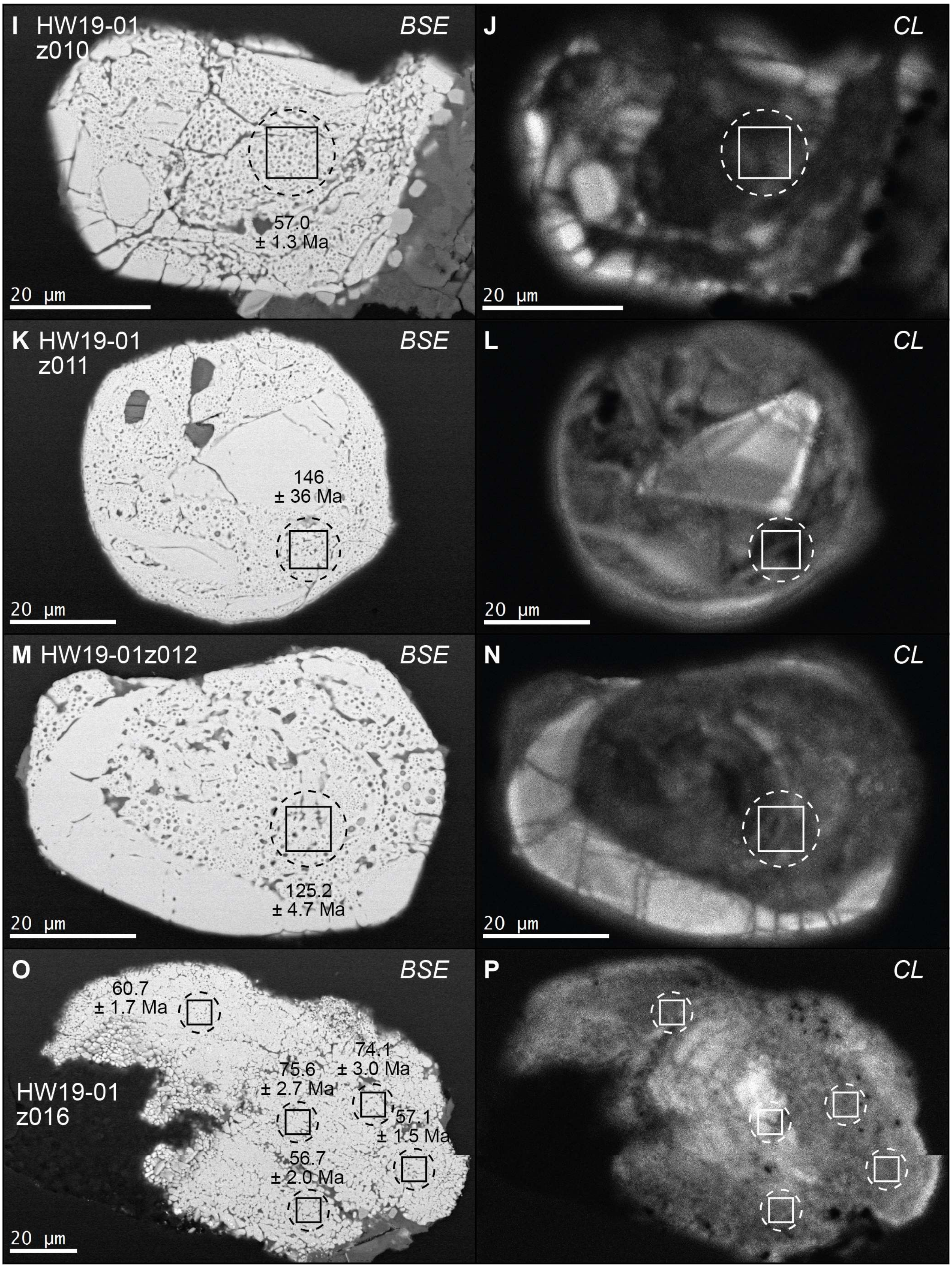

Fig. S5 continued.

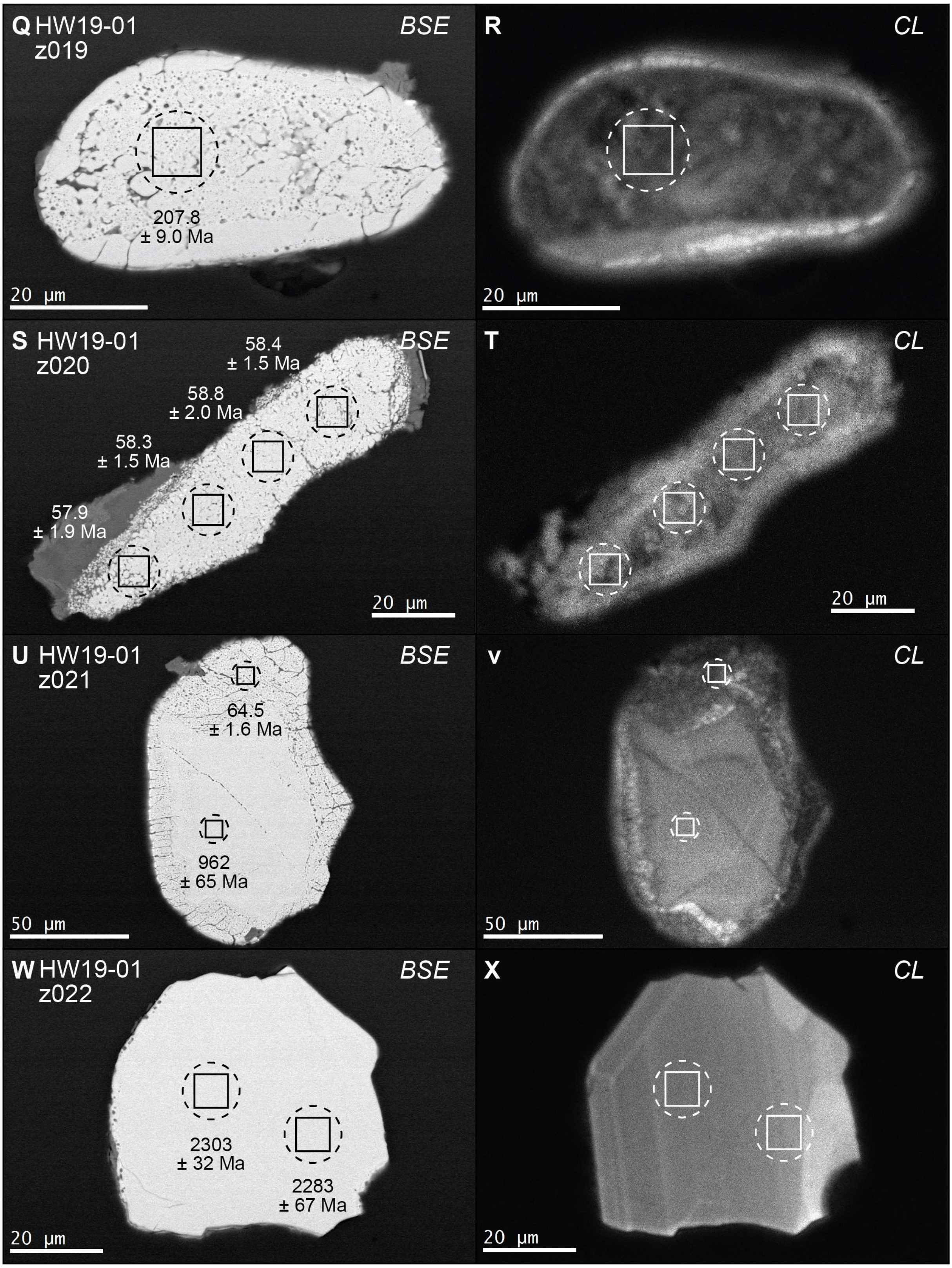

Fig. S5 continued.

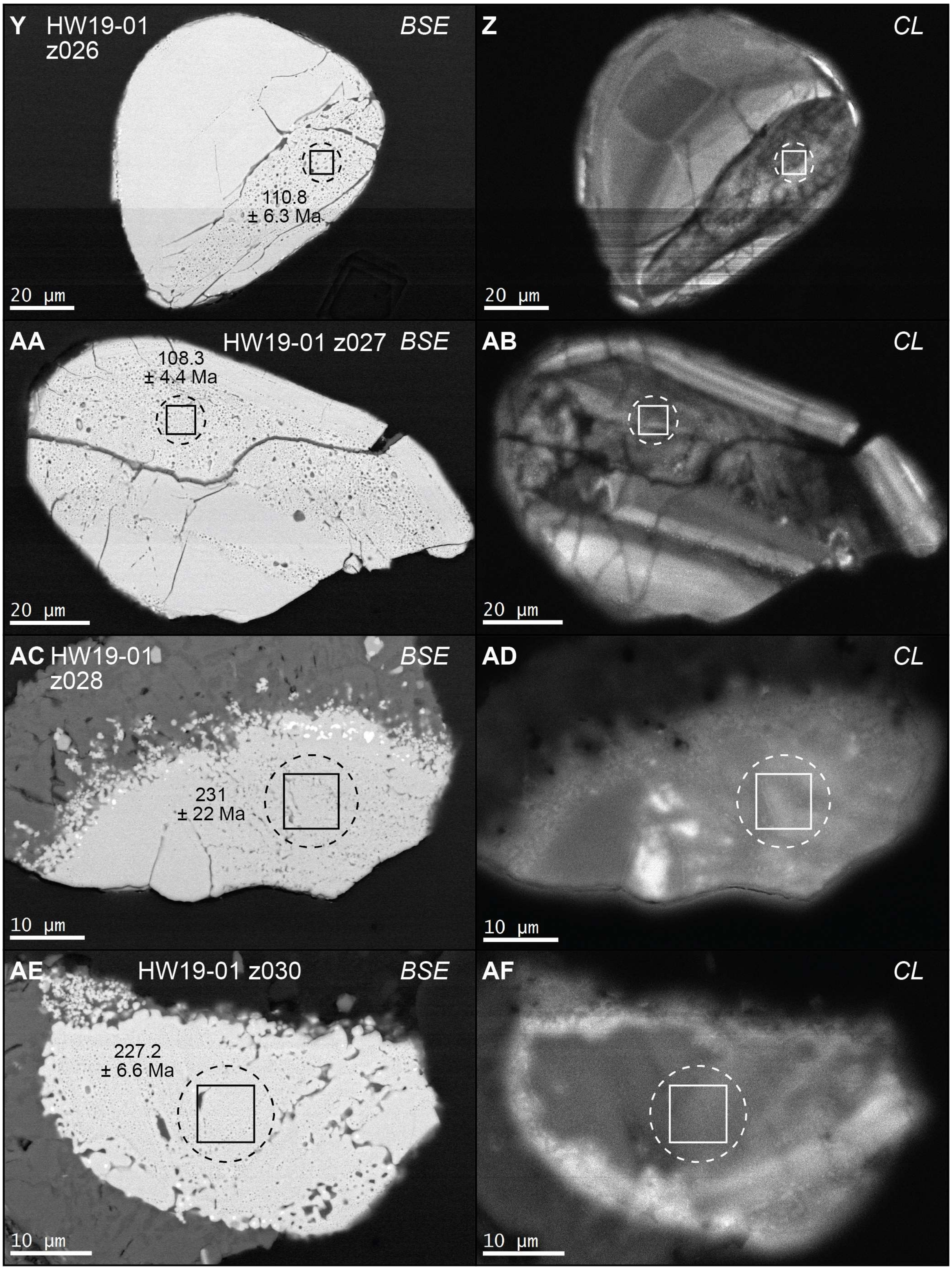

Fig. S5 continued.

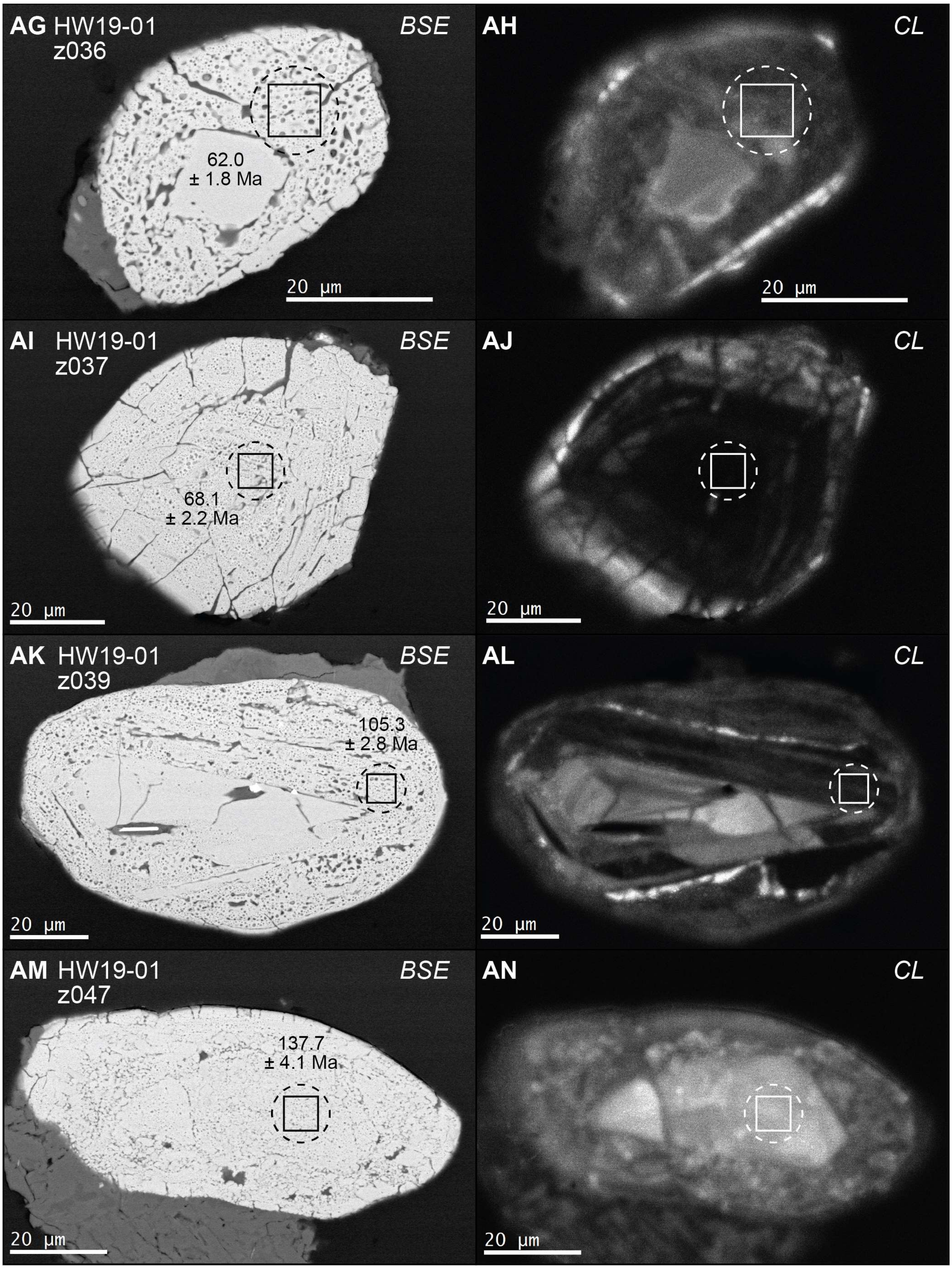

Fig. S5 continued.

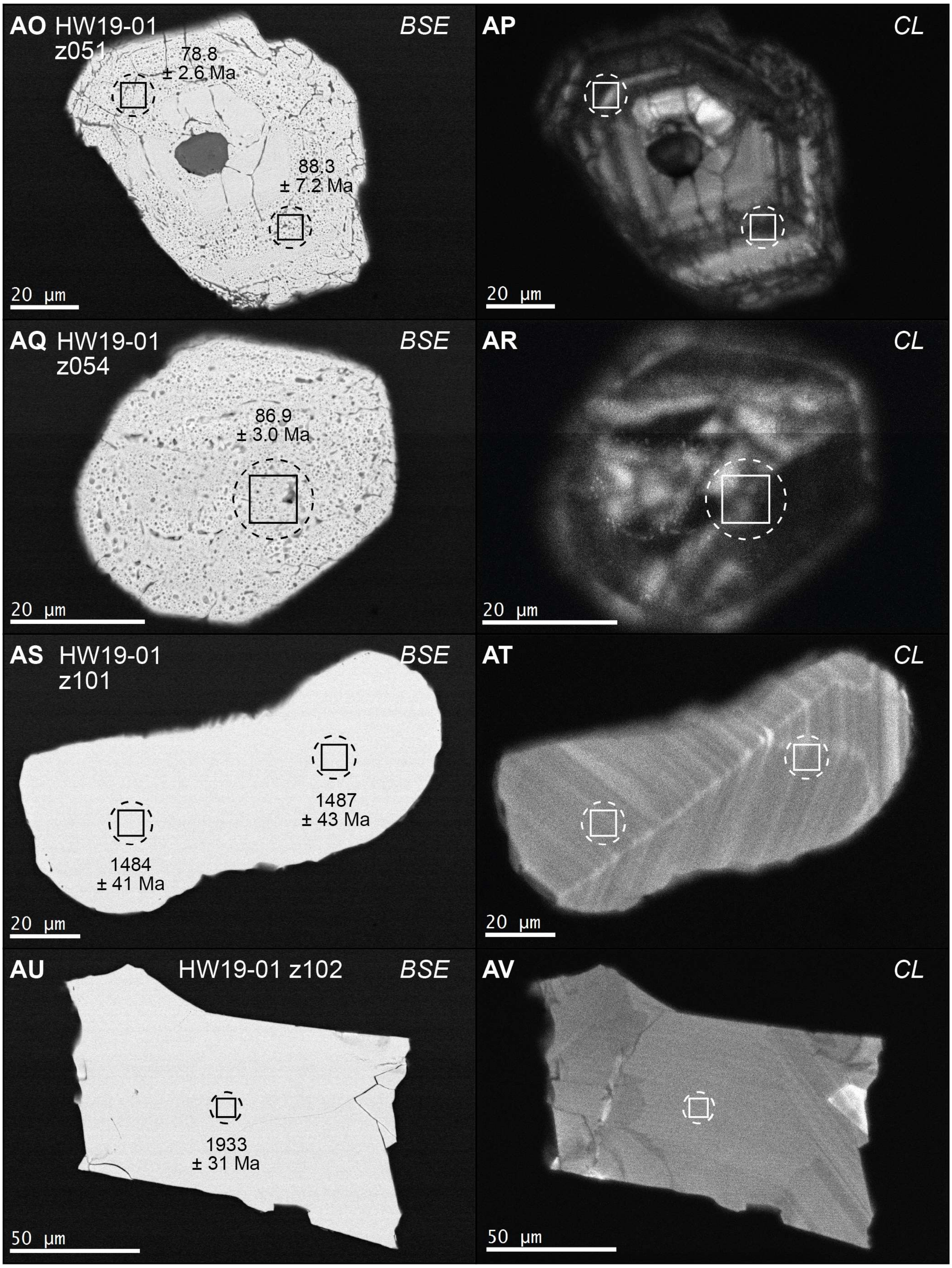

Fig. S5 continued.

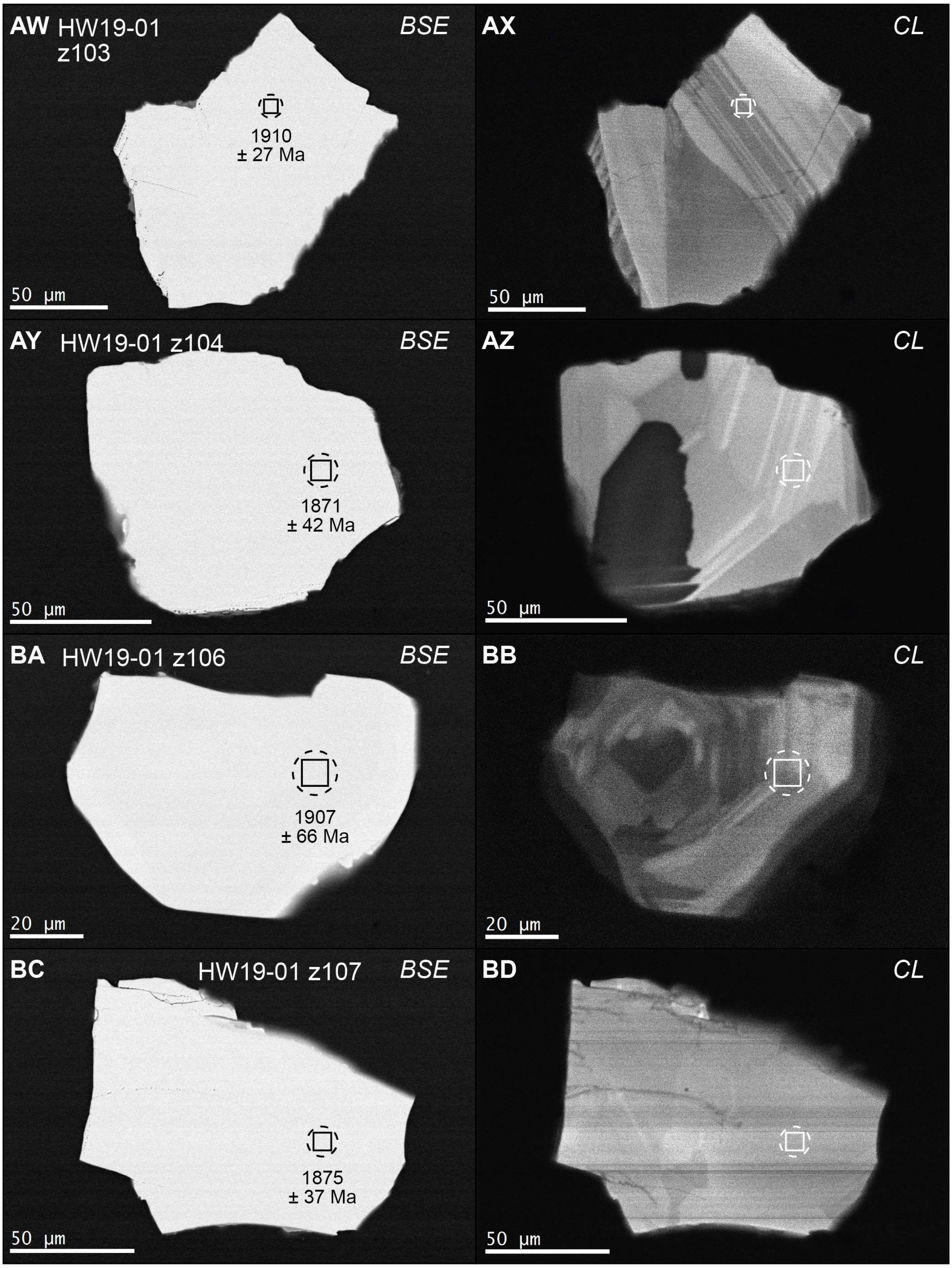

Fig. S5 continued.

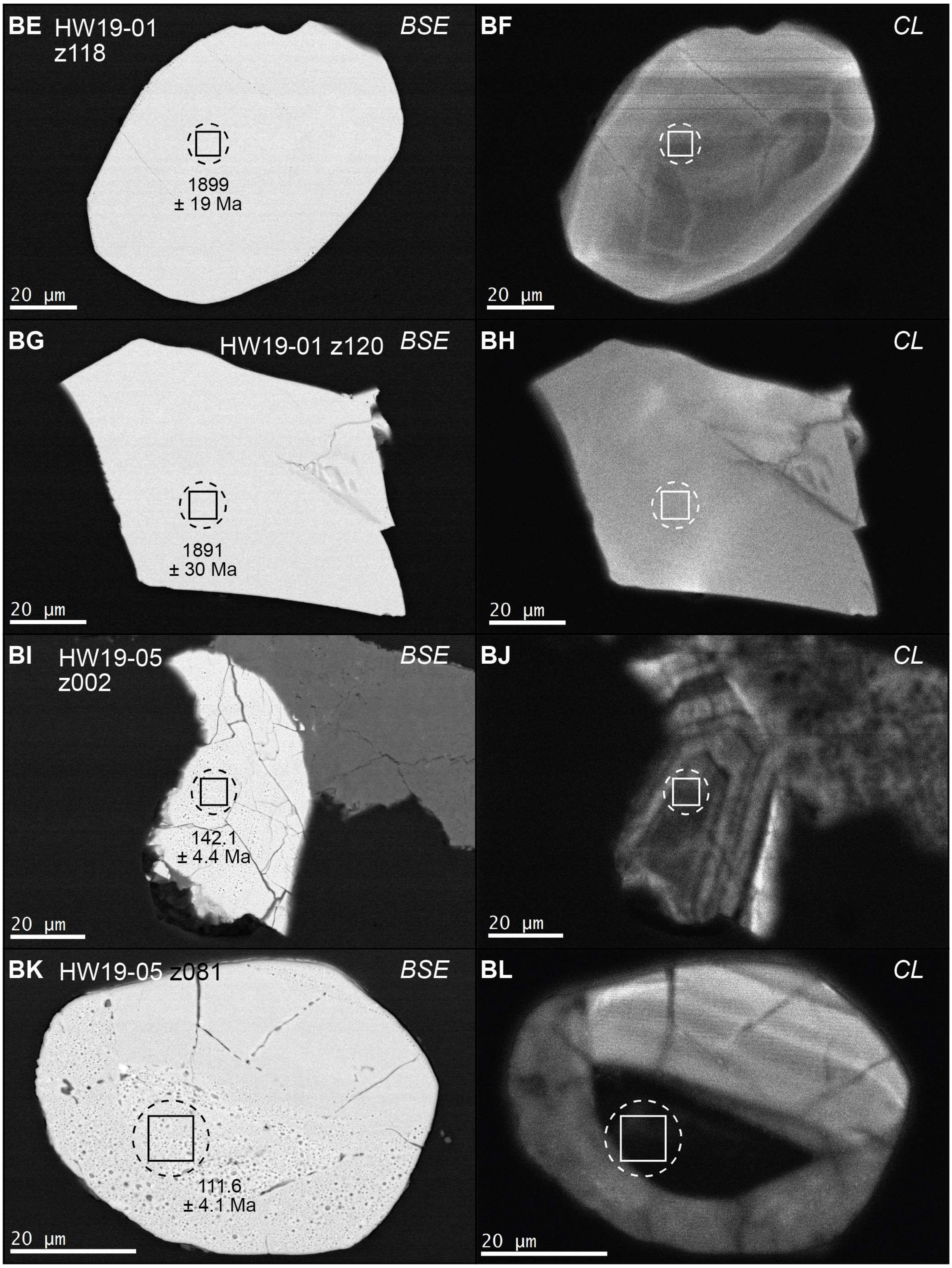

Fig. S5 continued.

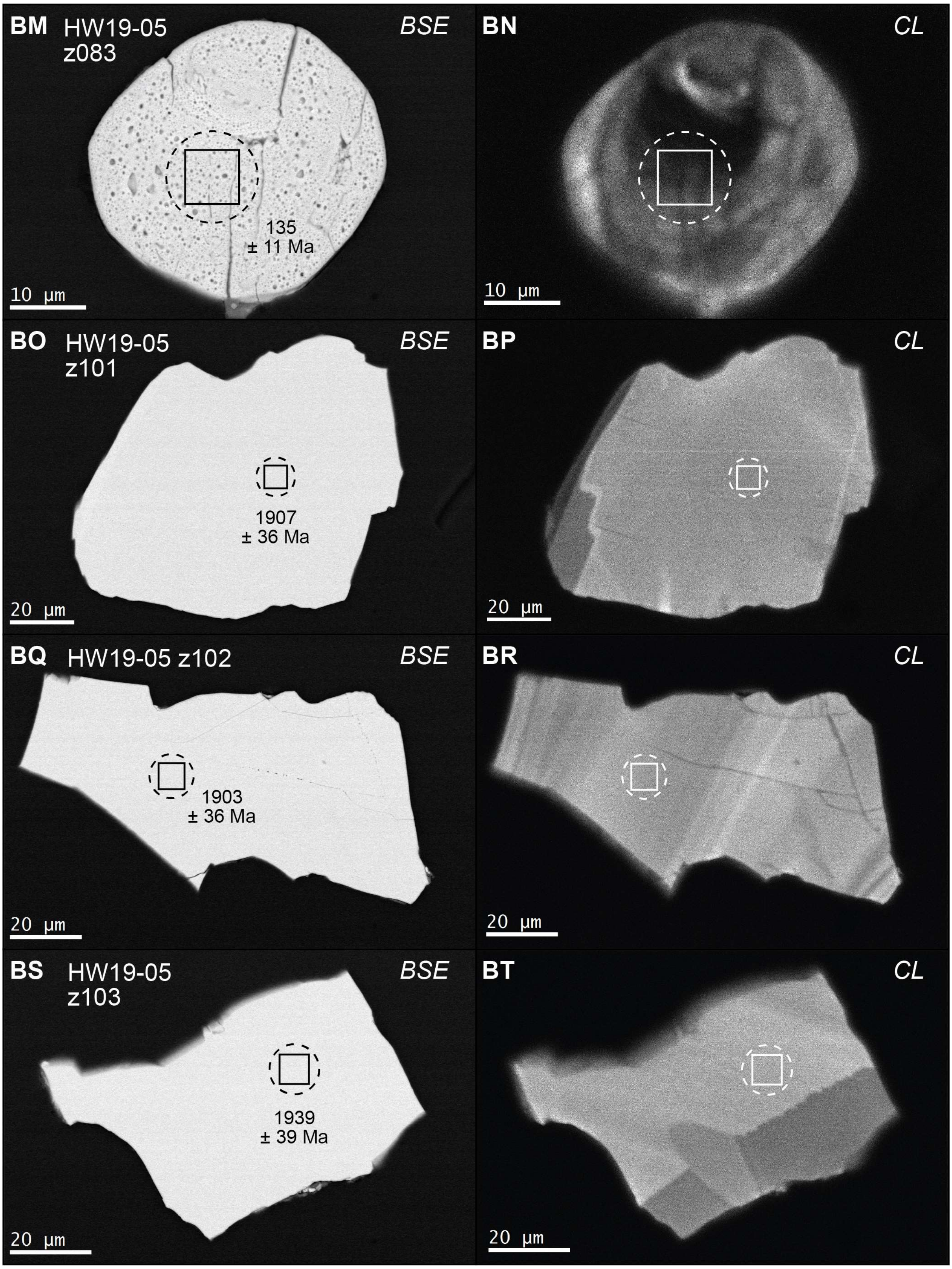

Fig. S5 continued.

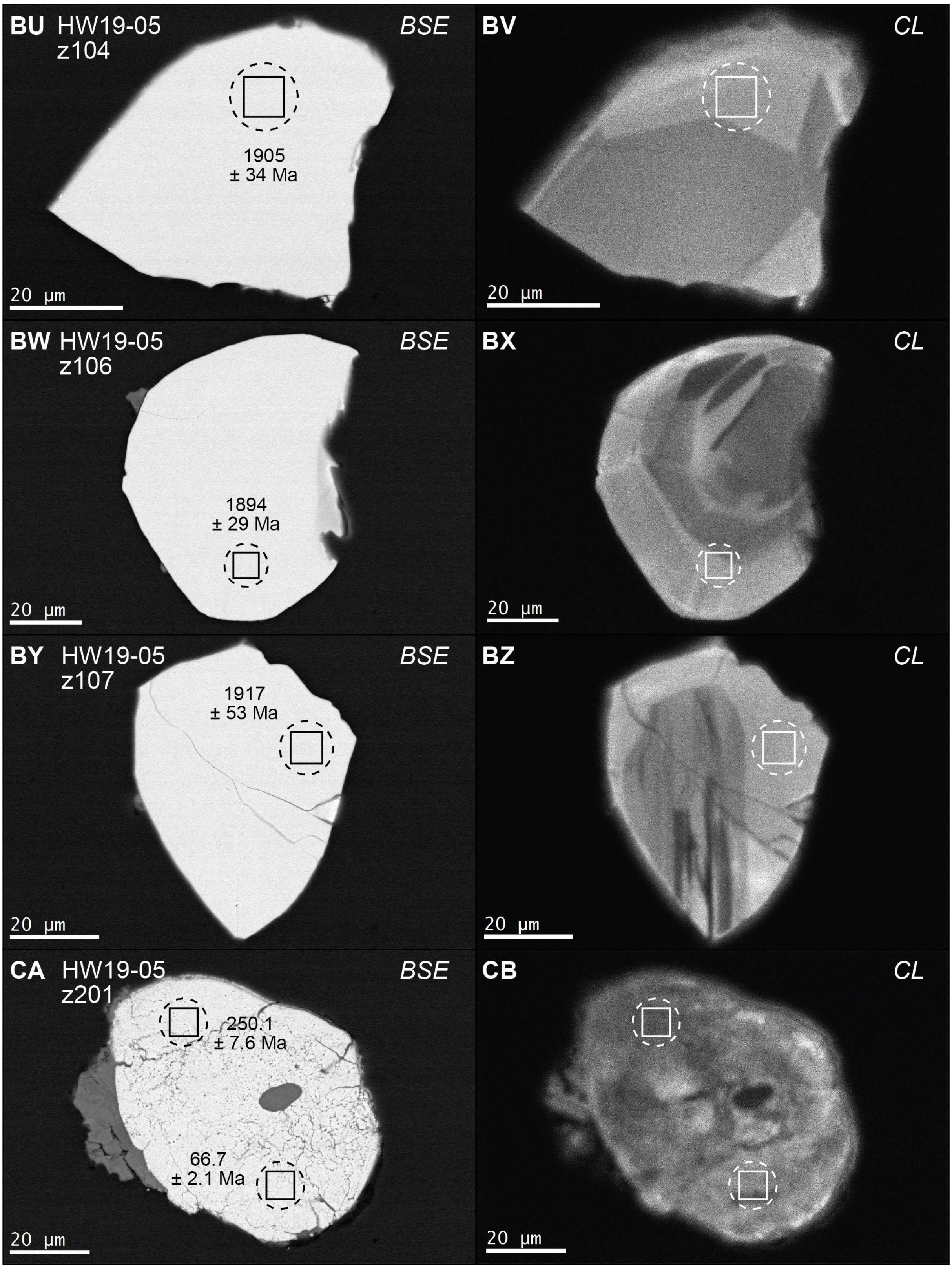

Fig. S5 continued.

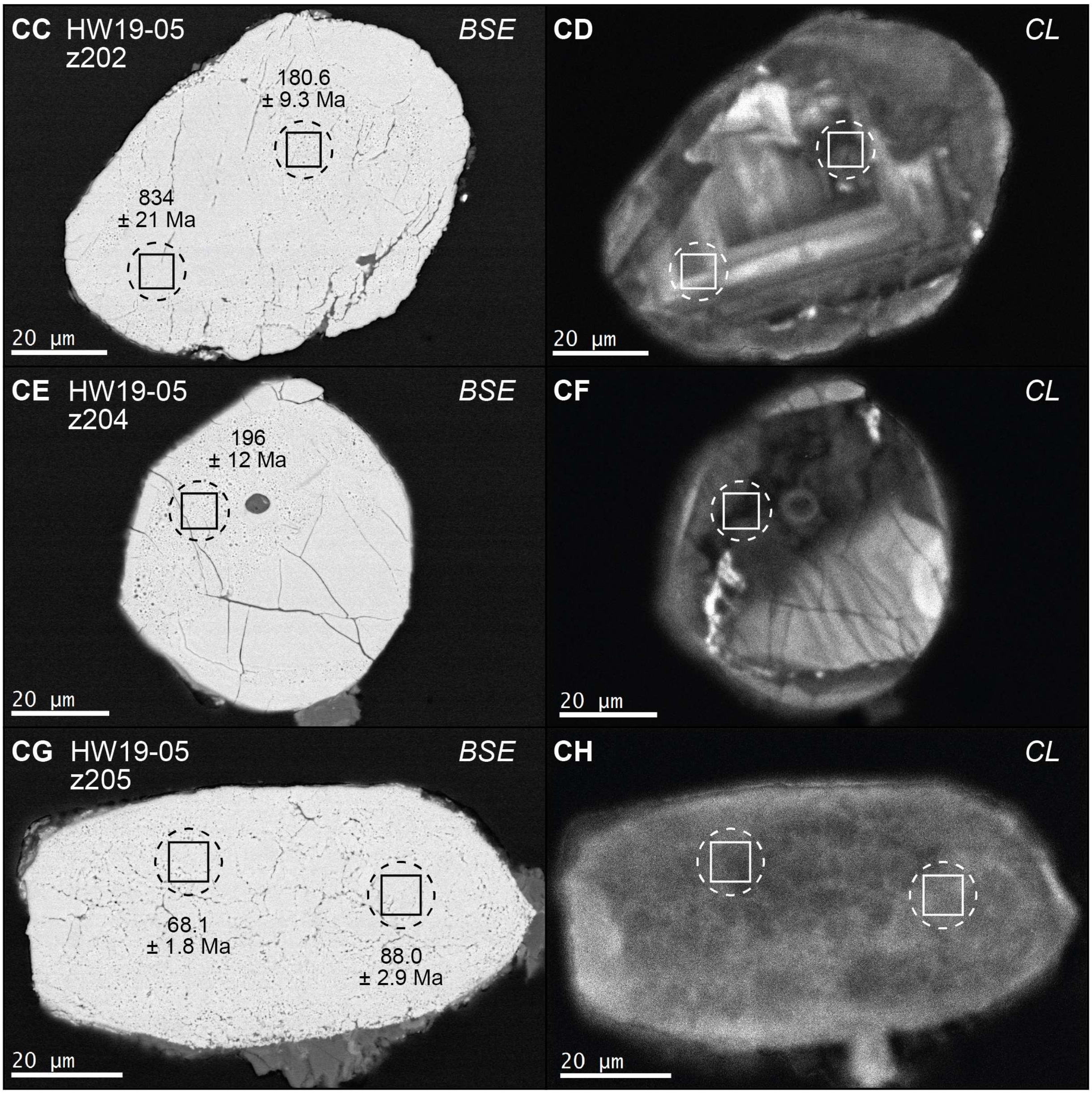

Fig. S5 continued.

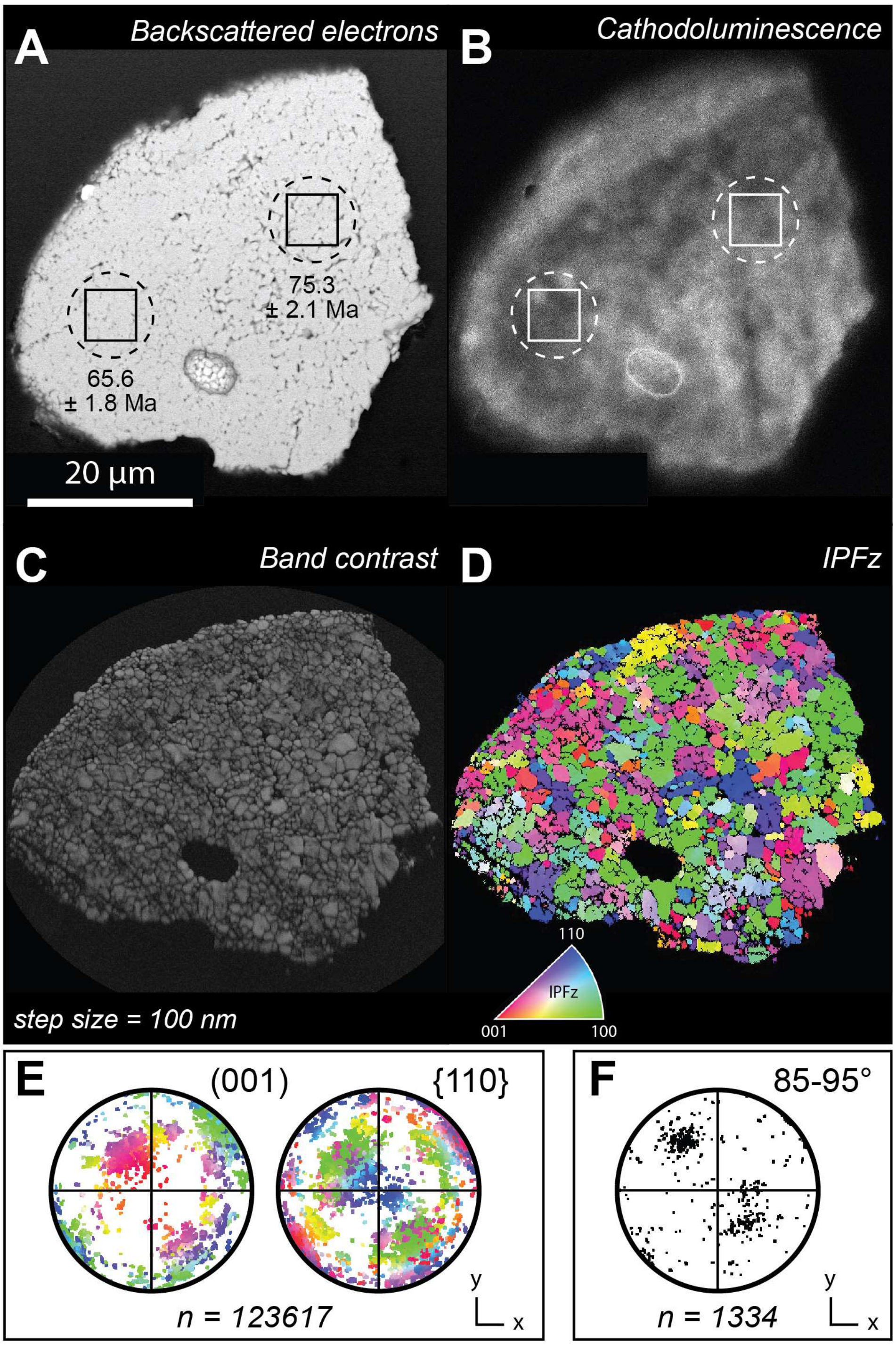

Fig. S6. Imaging and microstructural analysis of recrystallized zircon grain HW19-01 z009 from the Hiawatha impact structure. Dashed circle represents the ~10 μm-diameter U–Pb analysis pit and the square represents the approximately 6 × 6 μm^2 area that was sampled for U–Pb geochronology after the secondary ion beam passed through the field aperture (see methods). Apparent ages are $^{206}Pb/^{238}U$ ages. Uncertainties are 2σ.

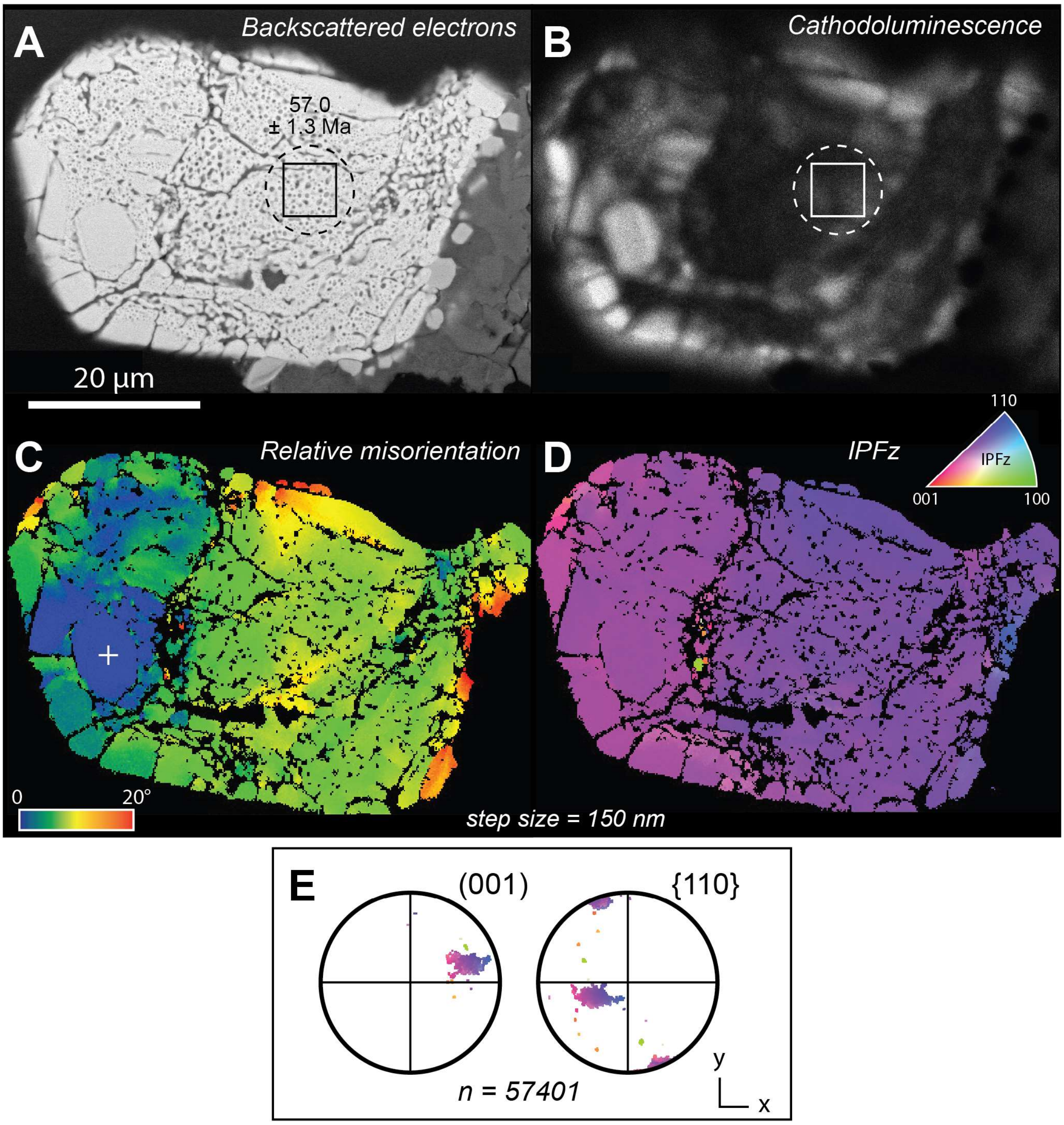

Fig. S7. Imaging and microstructural analysis of porous zircon grain HW19-01 z010 from the Hiawatha impact structure. See caption to Fig. S6 for more details.

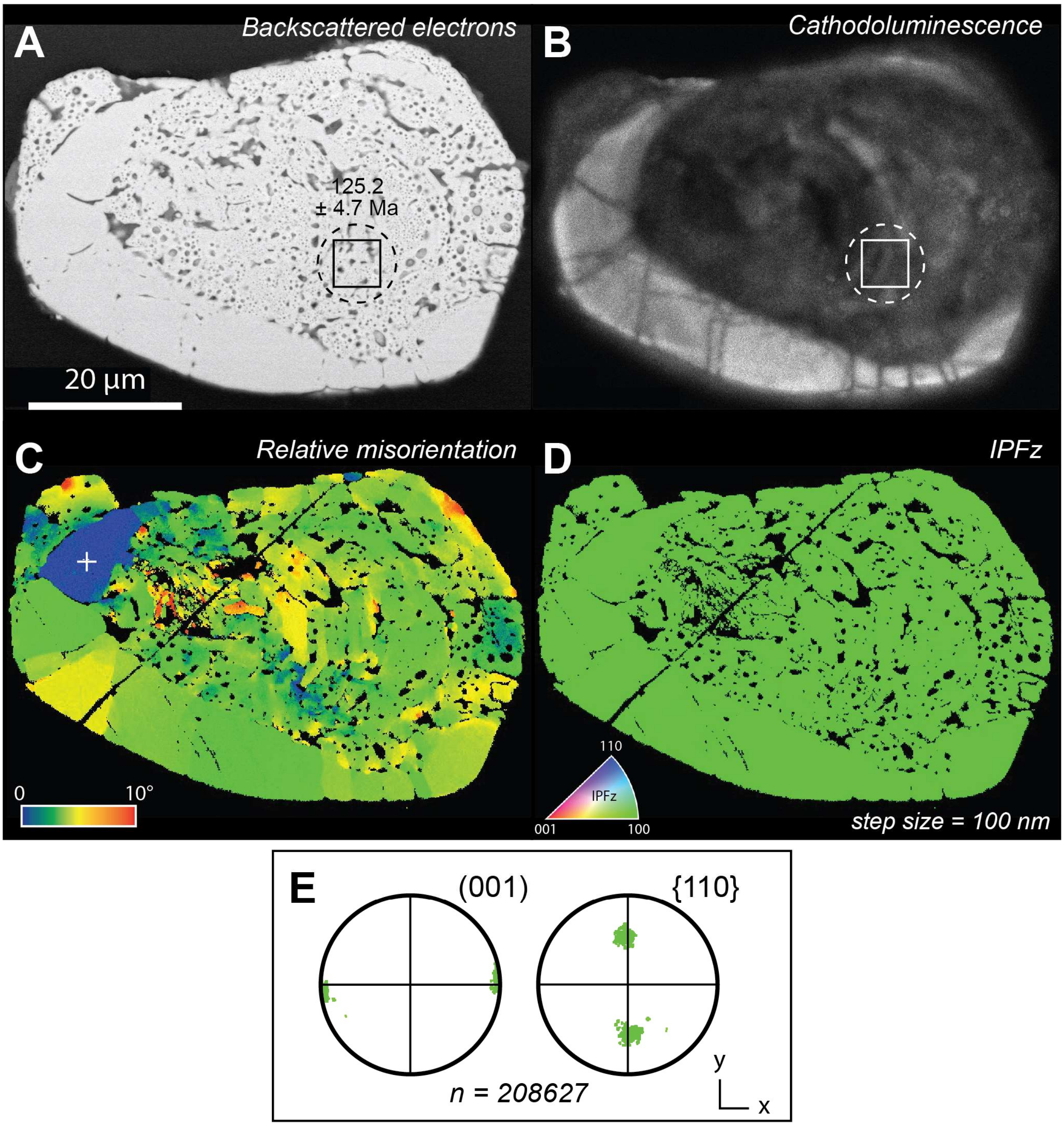

Fig. S8. Imaging and microstructural analysis of porous zircon grain HW19-01 z012 from the Hiawatha impact structure. See caption to Fig. S6 for more details. Linear feature in **C–D** is a scratch introduced during polishing.

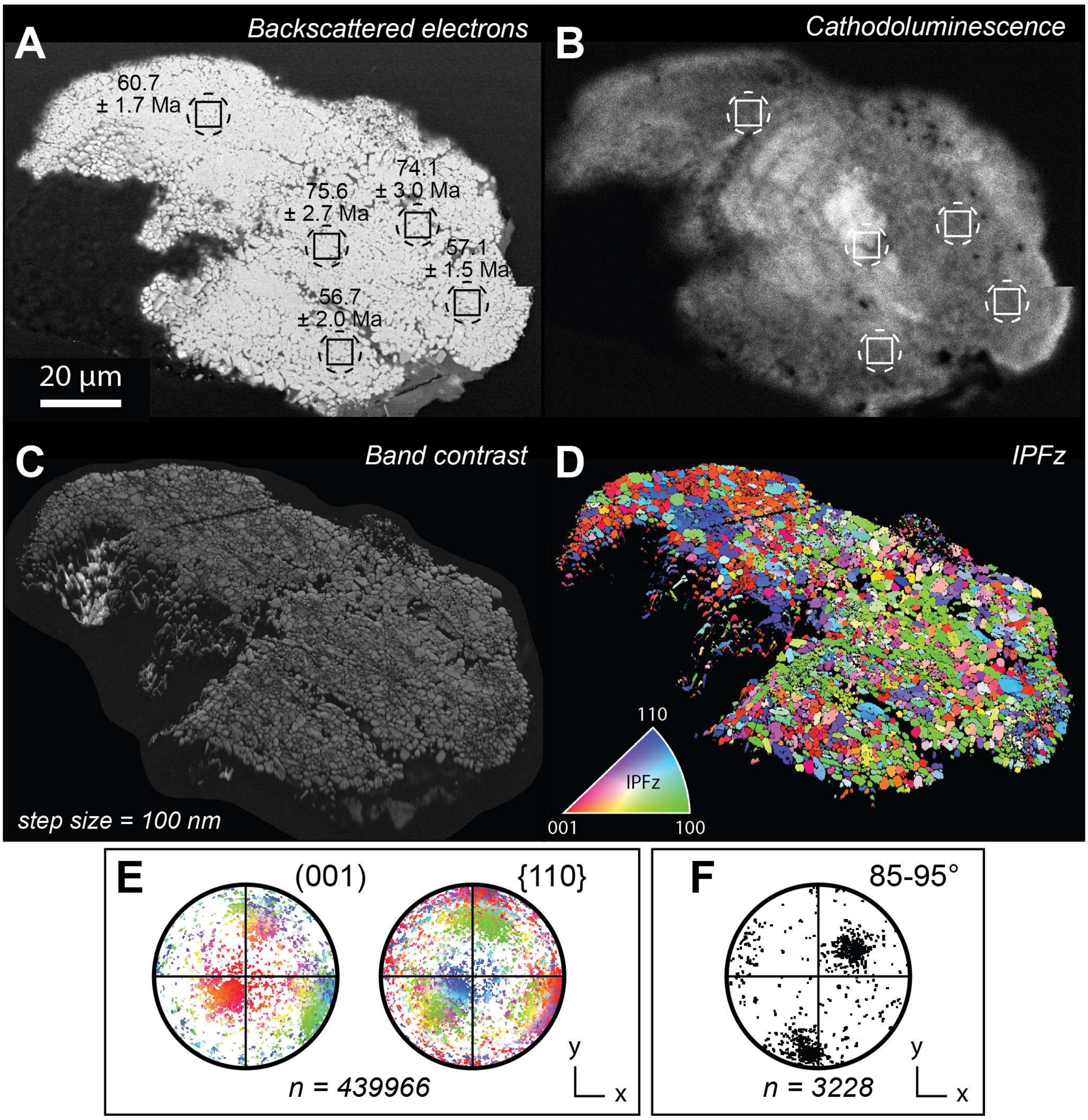

Fig. S9. Imaging and microstructural analysis of granular zircon grain HW19-01 z016 from the Hiawatha impact structure. See caption to Fig. S6 for more details.

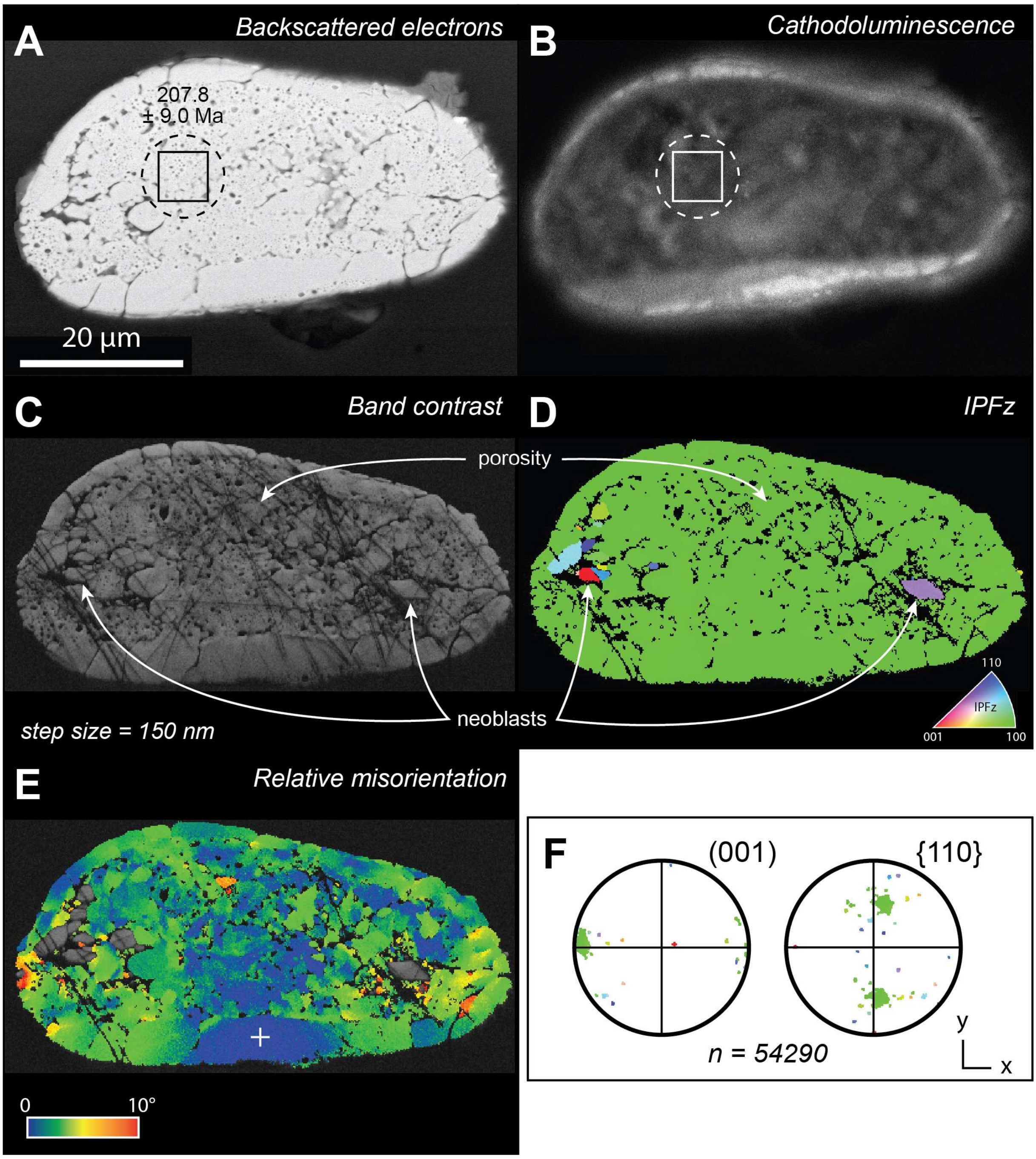

Fig. S10. Imaging and microstructural analysis of porous and partially recrystallized zircon grain HW19-01 z019 from the Hiawatha impact structure. See caption to Fig. S6 for more details. Linear features in **C** are scratches introduced during polishing.

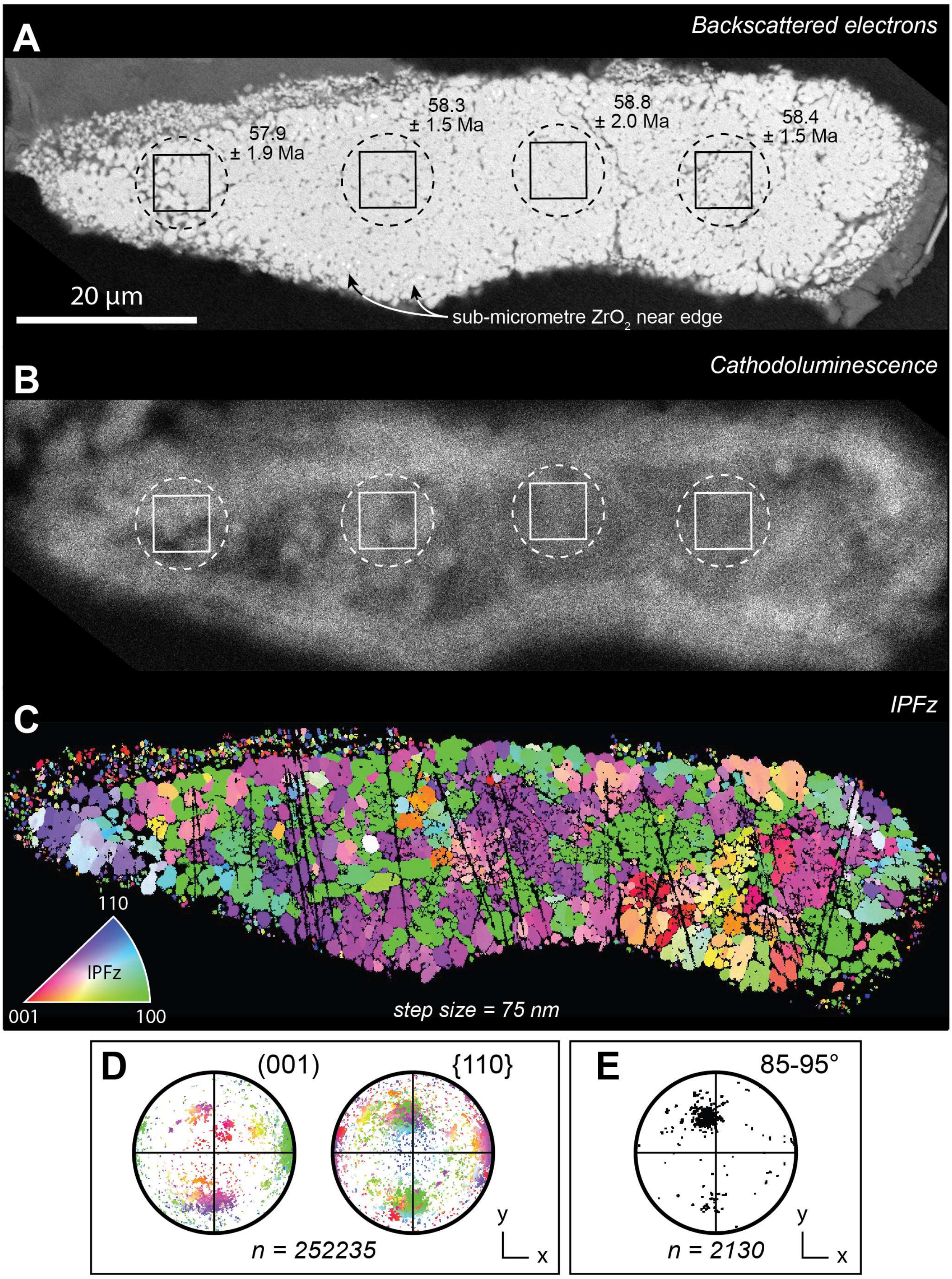

Fig. S11. Imaging and microstructural analysis of recrystallized zircon grain HW19-01 z020 from the Hiawatha impact structure. See caption to Fig. S6 for more details. Linear features in **C** are scratches introduced during polishing.

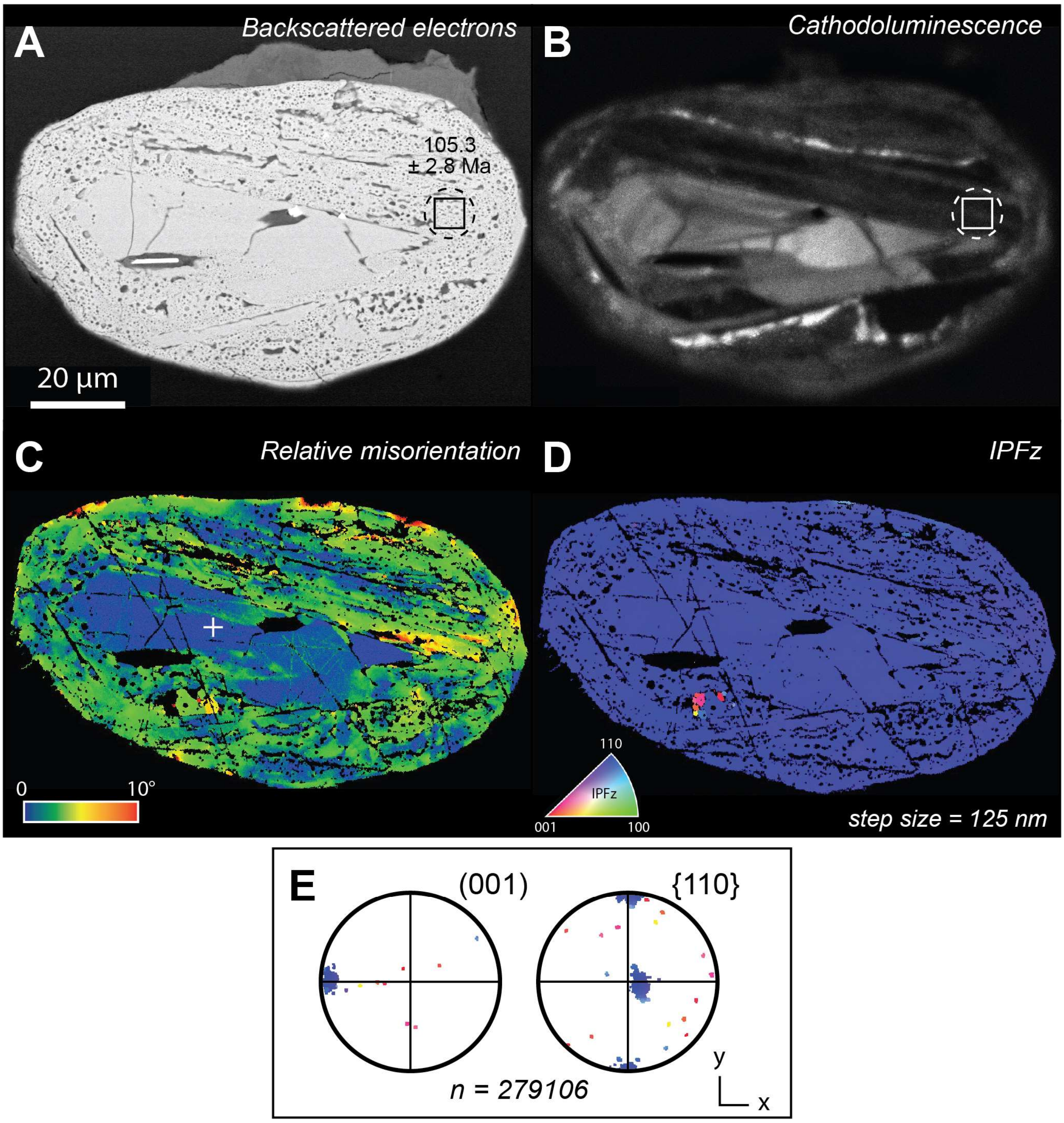

Fig. S12. Imaging and microstructural analysis of porous zircon grain HW19-01 z039 from the Hiawatha impact structure. See caption to Fig. S6 for more details.

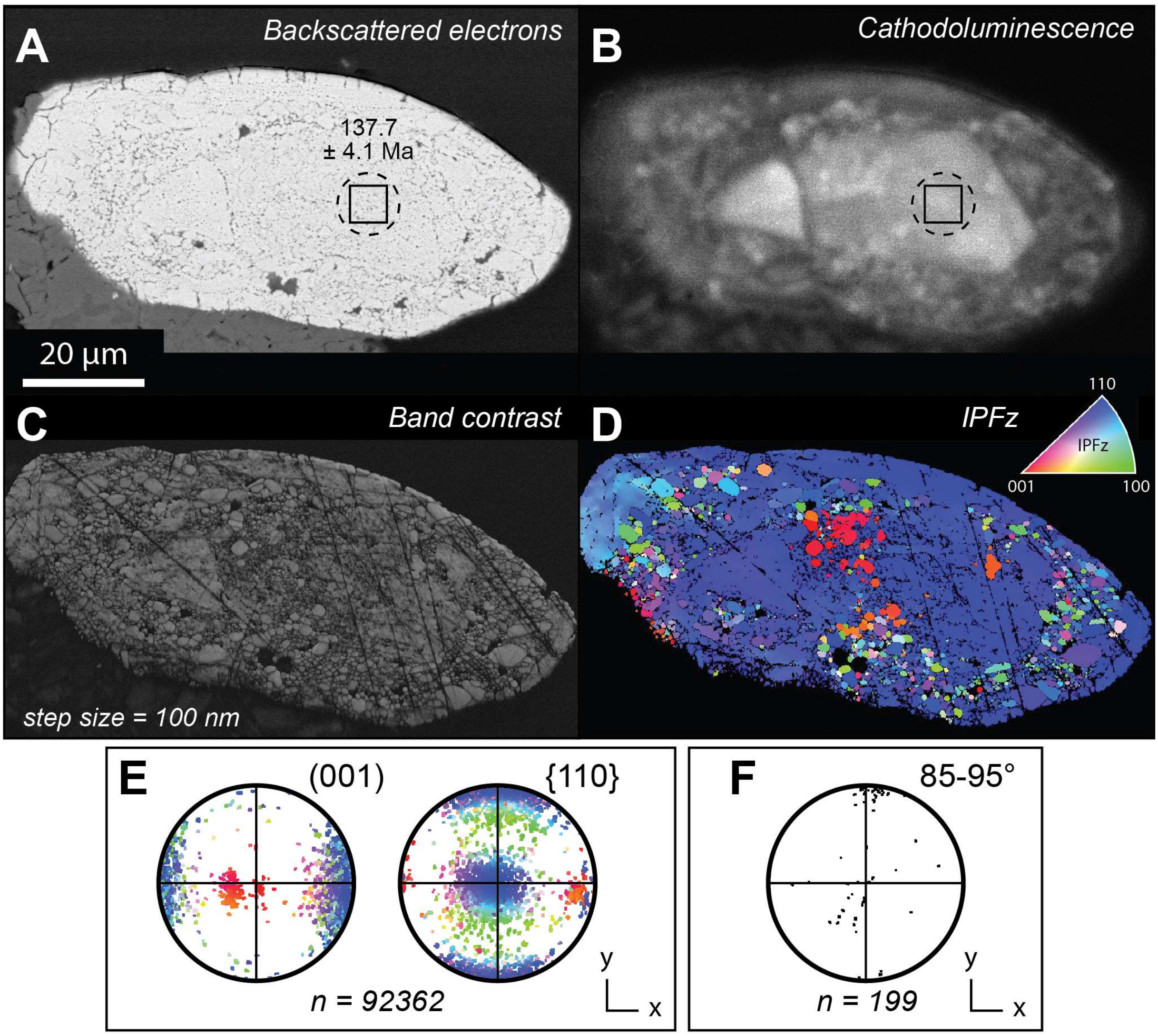

Fig. S13. Imaging and microstructural analysis of partially recrystallized zircon grain HW19-01 z047 from the Hiawatha impact structure. See caption to Fig. S6 for more details. Linear features in **C–D** are scratches introduced during polishing.

Data S1. (separate file) $^{40}Ar/^{39}Ar$ data for Hiawatha impactite grains.

Data S2. (separate file) Secondary ion mass spectrometry (SIMS) U–Pb data for Hiawatha zircon grains.

Data S3. (separate file) Secondary ion mass spectrometry (SIMS) U–Pb data for secondary reference material Temora 2.

REFERENCES AND NOTES

1. K. H. Kjær, N. K. Larsen, T. Binder, A. A. Bjørk, O. Eisen, M. A. Fahnestock, S. Funder, A. A. Garde, H. Haack, V. Helm, M. Houmark-Nielsen, K. K. Kjeldsen, S. A. Khan, H. Machguth, I. McDonald, M. Morlighem, J. Mouginot, J. D. Paden, T. E. Waight, C. Weikusat, E. Willerslev, J. A. MacGregor, A large impact crater beneath Hiawatha Glacier in northwest Greenland. *Sci. Adv.* **4**, eaar8173 (2018).

2. A. A. Garde, A. S. Søndergaard, C. Guvad, J. Dahl-Møller, G. Nehrke, H. Sanei, C. Weikusat, S. Funder, K. H. Kjær, N. K. Larsen, Pleistocene organic matter modified by the Hiawatha impact, northwest Greenland. *Geology* **48**, 867–871 (2020).

3. A. A. Garde, N. Keulen, T. Waight, Microporphyritic and microspherulitic melt grains, Hiawatha crater, Northwest Greenland: Implications for post-impact cooling rates, hydration, and the cratering environment. *GSA Bull.* (2021).

4. A. P. Nutman, P. R. Dawes, F. Kalsbeek, M. A. Hamilton, Palaeoproterozoic and Archaean gneiss complexes in northern Greenland: Palaeoproterozoic terrane assembly in the High Arctic. *Precambrian Res.* **161**, 419–451 (2008).

5. G. S. Collins, H. J. Melosh, R. A. Marcus, Earth Impact Effects Program: A Web-based computer program for calculating the regional environmental consequences of a meteoroid impact on Earth. *Meteorit. Planet. Sci.* **40**, 817–840 (2005).

6. M. N. Koppes, D. R. Montgomery, The relative efficacy of fluvial and glacial erosion over modern to orogenic timescales. *Nat. Geosci.* **2**, 644–647 (2009).

7. T. Cowton, P. Nienow, I. Bartholomew, A. Sole, D. Mair, Rapid erosion beneath the Greenland ice sheet. *Geology* **40**, 343–346 (2012).

8. A. Strunk, M. F. Knudsen, D. L. Egholm, J. D. Jansen, L. B. Levy, B. H. Jacobsen, N. K. Larsen, One million years of glaciation and denudation history in west Greenland. *Nat. Commun.* **8**, 14199 (2017).

9. N. E. Young, J. P. Briner, J. Maurer, J. M. Schaefer, ^{10}Be measurement in bedrock constrain erosion beneath the Greenland Ice Sheet margin. *Geophys. Res. Lett.* **43**, 11708–11719 (2016).

10. P. R. Bierman, J. D. Shakun, L. B. Corbett, S. R. Zimmerman, D. H. Rood, A persistent and dynamic East Greenland Ice Sheet over the past 7.5 million years. *Nature* **540**, 256–260 (2016).

11. R. A. F. Grieve, Petrology and chemistry of the impact melt at Mistastin Lake crater, Labrador, *GSM Bull.* **86**, 1617–1629 (1975).

12. J. Whitehead, R. A. F. Grieve, J. G. Spray, Mineralogy and petrology of melt rocks from the Popigai impact structure, Siberia. *Meteorit. Planet. Sci.* **37**, 623–647 (2002).

13. A. E. Pickersgill, S. J. Jaret, L. Pittarello, J. Fritz, R. S. Harris, Shock effects in feldspars: An overview, in *Large Meteorite Impacts and Planetary Evolution VI*, W. U. Reimold, C. Koeberl, Eds. (Geological Society of America, 2021), vol. 550, pp. 507–535.

14. S. Kelley, Excess argon in K–Ar and Ar–Ar geochronology. *Chem. Geol.* **188**, 1–22 (2002).

15. A. J. Schaen, B. R. Jicha, K. V. Hodges, P. Vermeesch, M. E. Stelten, C. M. Mercer, D. Phillips, T. A. Rivera, F. Jourdan, E. L. Matchan, S. R. Hemming, L. E. Morgan, S. P. Kelley, W. S. Cassata, M. T. Heizler, P. M. Vasconcelos, J. A. Benowitz, A. A. P. Koppers, D. F. Mark, E. M. Niespolo, C. J. Sprain, W. E. Hames, K. F. Kuiper, B. D. Turrin, P. R. Renne, J. Ross, S. Nomade, H. Guillou, L. E. Webb, B. A. Cohen, A. T. Calvert, N. Joyce, M. Ganerød, J. Wijbrans, O. Ishizuka, H. He, A. Ramirez, J. A. Pfänder, M. Lopez-Martínez, H. Qiu, B. S. Singer, Interpreting and reporting ^{40}Ar/^{39}Ar geochronologic data. *Geol. Soc. Am. Bull.* **133**, 461–487 (2021).

16. A. J. Cavosie, N. E. Timms, T. M. Erickson, J. J. Hagerty, F. Hörz, Transformations to granular zircon revealed: Twinning, reidite, and ZrO_2 in shocked zircon from Meteor Crater (Arizona, USA). *Geology* **44**, 703–706 (2016).

17. A. J. Cavosie, N. E. Timms, L. Ferrière, P. Rochette, FRIGN zircon–The only terrestrial mineral diagnostic of high-pressure and high-temperature shock deformation. *Geology* **46**, 891–894 (2018).

18. E. Kovaleva, M. A. Kusiak, G. G. Kenny, M. J. Whitehouse, G. Habler, A. Schreiber, R. Wirth, Nano-scale investigation of granular neoblastic zircon, Vredefort impact structure, South Africa: Evidence for complete shock melting. *Earth Planet. Sci. Lett.* **565**, 116948 (2021).

19. M. Pino, A. M. Abarzúa, G. Astorga, A. Martel-Cea, N. Cossio-Montecinos, R. X. Navarro, M. Paz Lira, R. Labarca, M. A. LeCompte, V. Adedeji, C. R. Moore, T. E. Bunch, C. Mooney, W. S. Wolbach, A. West, J. P. Kennett, Sedimentary record from Patagonia, southern Chile supports cosmic-impact triggering of biomass burning, climate change, and megafaunal extinctions at 12.8 ka. *Sci. Rep.* **9**, 4413 (2019).

20. E. A. Silber, B. C. Johnson, E. Bjonnes, J. A. MacGregor, N. K. Larsen, S. E. Wiggins, Effect of ice sheet thickness on formation of the Hiawatha impact crater. *Earth Planet. Sci. Lett.* **566**, 116972 (2021).

21. A. S. P. Rae, G. S. Collins, J. V. Morgan, T. Salge, G. L. Christeson, J. Leung, J. Lofi, S. P. S. Gulick, M. Poelchau, U. Riller, C. Gebhardt, R. A. F. Grieve, G. R. Osinski, Impact-induced porosity and microfracturing at the chicxulub impact structure. *J. Geophys. Res. Planets* **124**, 1960–1978 (2019).

22. J. L. Bamber, J. A. Griggs, R. T. W. L. Hurkmans, J. A. Dowdeswell, S. P. Gogineni, I. Howat, J. Mouginot, J. Paden, S. Palmer, E. Rignot, D. Steinhage, A new bed elevation dataset for Greenland. *Cryosphere* **7**, 499–510 (2013).

23. M. Morlighem, C. N. Williams, E. Rignot, L. An, J. E. Arndt, J. L. Bamber, G. Catania, N. Chauché, J. A. Dowdeswell, B. Dorschel, I. Fenty, K. Hogan, I. Howat, A. Hubbard, M. Jakobsson, T. M. Jordan, K. K. Kjeldsen, R. Millan, L. Mayer, J. Mouginot, B. P. Y. Noël, C. O'Cofaigh, S. Palmer, S. Rysgaard, H. Seroussi, M. J. Siegert, P. Slabon, F. Straneo, M. R. van den Broeke, W. Weinrebe, M. Wood, K. B. Zinglersen, BedMachine v3: Complete bed

topography and ocean bathymetry mapping of Greenland from multibeam echo sounding combined with mass conservation. *Geophys. Res. Lett.* **44**, 11051–11061 (2017).

24. B. A. Keisling, L. T. Nielsen, C. S. Hvidberg, R. Nuterman, R. M. DeConto, Pliocene–Pleistocene megafloods as a mechanism for Greenlandic megacanyon formation. *Geology* **48**, 737–741 (2020).

25. J. A. MacGregor, W. F. Bottke Jr., M. A. Fahnestock, J. P. Harbeck, K. H. Kjær, J. D. Paden, D. E. Stillman, M. Studinger, A possible second large subglacial impact crater in northwest Greenland. *Geophys. Res. Lett.* **46**, 1496–1504 (2019).

26. G. J. G. Paxman, J. Austermann, K. J. Tinto, A fault-bounded palaeo-lake basin preserved beneath the Greenland Ice Sheet. *Earth Planet. Sci. Lett.* **553**, 116647 (2021).

27. G. Dam, M. Sønderholm, E. V. Sørensen, Inherited basement canyons: Impact on sediment distribution in the North Atlantic. *Terra Nova* **32**, 272–280 (2020).

28. C. J. Williams, B. LePage, A. Johnson, D. R. Vann, Structure, biomass, and productivity of a late Paleocene arctic forest. *Proc. Acad. Nat. Sci. Phila.* **158**, 107–127 (2009).

29. J. T. Bessette, D. M. Schroeder, T. M. Jordan, J. A. MacGregor, radar-sounding characterization of the subglacial groundwater table beneath Hiawatha Glacier, Greenland. *Geophys. Res. Lett.* **48**, e2020GL091432 (2021).

30. J. A. MacGregor, M. A. Fahnestock, G. A. Catania, A. Aschwanden, G. D. Clow, W. T. Colgan, S. P. Gogineni, M. Morlighem, S. M. J. Nowicki, J. D. Paden, S. F. Price, H. Seroussi, A synthesis of the basal thermal state of the Greenland Ice Sheet. *Case Rep. Med.* **121**, 1328–1350 (2016).

31. J. A. MacGregor, M. A. Fahnestock, W. T. Colgan, N. K. Larsen, K. K. Kjeldsen, J. M. Welker, The age of surface-exposed ice along the northern margin of the Greenland Ice Sheet. *J. Glaciol.* **66**, 667–684 (2020).

32. T. Westerhold, U. Röhl, T. Frederichs, S. M. Bohaty, J. C. Zachos, Astronomical calibration of the geological timescale: Closing the middle Eocene gap. *Clim. Past* **11**, 1181–1195 (2015).

33. M. Storey, R. A. Duncan, C. C. Swisher, Paleocene-Eocene thermal maximum and the opening of the northeast Atlantic. *Science* **316**, 587–589 (2007).

34. M. Storey, R. A. Duncan, A. K. Pedersen, L. M. Larsen, H. C. Larsen, $^{40}Ar/^{39}Ar$ geochronology of the West Greenland Tertiary volcanic province. *Earth Planet. Sci. Lett.* **160**, 569–586 (1998).

35. L. M. Larsen, A. K. Pedersen, C. Tegner, R. A. Duncan, N. Hald, J. G. Larsen, Age of Tertiary volcanic rocks on the West Greenland continental margin: Volcanic evolution and event correlation to other parts of the North Atlantic Igneous Province. *Geol. Mag.* **153**, 487–511 (2016).

36. J. F. McHone, R. B. Sorkhabi, Apatite fission-track age of Marquez Dome impact structure, Texas. *Lunar Planet. Sci.* **25**, 881–882 (1994).

37. V. L. Sharpton, J. W. Gibson Jr., The Marquez Dome impact structure, Leon County, Texas (abstract). *Lunar Planet. Sci.* **24**, 1136–1137 (1990).

38. B. C. Johnson, T. J. Bowling, Where have all the craters gone? Earth's bombardment history and the expected terrestrial cratering record. *Geology* **42**, 587–590 (2014).

39. S. Hergarten, T. Kenkmann, The number of impact craters on Earth: Any room for further discoveries? *Earth Planet. Sci. Lett.* **425**, 187–192 (2015).

40. F. Jourdan, P. R. Renne, W. U. Reimold, An appraisal of the ages of terrestrial impact structures. *Earth Planet. Sci. Lett.* **286**, 1–13 (2009).

41. S. P. Kelley, S. C. Sherlock, The geochronology of impact craters, in *Impact Cratering: Processes and Products*, G. R. Osinski, E. Pierazzo, Eds. (Wiley-Blackwell, 2012), pp. 240–253.

42. M. Schmieder, D. A. Kring, Earth's impact events through geologic time: A list of recommended ages for terrestrial impact structures and deposits. *Astrobiology* **20**, 91–141 (2020).

43. A. K. Pedersen, Graphitic andesite tuffs resulting from high-Mg tholeiite and sediment interaction; Nûgssuaq, West Greenland. *Bull. Geol. Soc. Denmark* **27**, 117–130 (1978).

44. E. Robin, N. H. Swinburne, L. Froget, R. Rocchia, J. Gayraud, Characteristics and origin of the glass spherules from the Paleocene flood basalt province of western Greenland. *Geochim. Cosmochim. Acta* **60**, 815–830 (1996).

45. S. V. Margolis, N. H. M. Swinburne, F. Asaro, C. C. Swisher, M. McWilliams, H. J. Hansen, A. K. Pedersen, Possible impact ejecta in the Palaeocene flood basalt province of west Greenland. *Eos Trans. AGU* **72**, 278.

46. A. P. Jones, A. T. Kearsley, C. R. L. Friend, E. Robin, A. Beard, A. Tamura, S. Trickett, P. Claeys, Are there signs of a large Paleocene impact, preserved around Disko Bay, West Greenland? Nuussuaq spherule beds origin by impact instead of volcanic eruption?, in *Large Meteorite Impacts and Planetary Evolution III*, T. Kenkmann, F. Hörz, A. Deutsch, Eds. (Geological Society of America, 2005), vol. 384, pp. 507–535.

47. A. K. Pedersen, L. M. Larsen, G. K. Pedersen, Lithostratigraphy, geology and geochemistry of the volcanic rocks of the Vaigat Formation on Disko and Nuussuaq, Paleocene of West Greenland. *GEUS Bull.* **39**, 1–244 (2017).

48. M. F. Schaller, M. K. Fung, J. D. Wright, M. E. Katz, D. V. Kent, Impact ejecta at the Paleocene-Eocene boundary. *Science* **354**, 225–229 (2016).

49. M. F. Schaller, M. K. Fung, The extraterrestrial impact evidence at the Palaeocene–Eocene boundary and sequence of environmental change on the continental shelf. *Phil. Trans. R. Soc. A* **376**, 20170081 (2018).

50. M. F. Schaller, B. D. Turrin, M. K. Fung, M. E. Katz, C. C. Swisher, Initial ^{40}Ar-^{39}Ar ages of the Paleocene-Eocene boundary impact spherules. *Geophys. Res. Lett.* **46**, 9091–9102 (2019).

51. N. J. Shackleton, M. A. Hall, A. Boersma, Oxygen and carbon isotope data from Leg 74 foraminifers, in *Initial Reports of the Deep Sea Drilling Project* (U.S. Government Printing Office, 1984), vol. 74, pp. 599–612.

52. R. M. Corfield, J. E. Cartlidge, Oceanographic and climatic implications of the Palaeocene carbon isotope maximum. *Terra Nova* **4**, 443–455 (1992).

53. K. Littler, U. Röhl, T. Westerhold, J. C. Zachos, A high-resolution benthic stable-isotope record for the South Atlantic: Implications for orbital-scale changes in Late Paleocene–Early Eocene climate and carbon cycling. *Earth Planet. Sci. Lett.* **401**, 18–30 (2014).

54. J. C. Zachos, G. R. Dickens, R. E. Zeebe, An early Cenozoic perspective on greenhouse warming and carbon-cycle dynamics. *Nature* **451**, 279–283 (2008).

55. J. D. O'Keefe, T. J. Ahrens, Impact production of CO_2 by the cretaceous/tertiary extinction bolide and the resultant heating of the Earth. *Nature* **338**, 247–249 (1989).

56. O. B. Toon, K. Zahnle, D. Morrison, R. P. Turco, C. Covey, Environmental perturbations caused by the impacts of asteroids and comets. *Rev. Geophys.* **35**, 41–78 (1997).

57. E. Pierazzo, D. A. Kring, H. J. Melosh, Hydrocode simulation of hte Chicxulub impact event and the production of climatically active gases. *J. Geophys. Res. Planets* **103**, 28607–28625 (1998).

58. P. Schulte, L. Alegret, I. Arenillas, J. A. Arz, P. J. Barton, P. R. Bown, T. J. Bralower, G. L. Christeson, P. Claeys, C. S. Cockell, G. S. Collins, A. Deutsch, T. J. Goldin, K. Goto, J. M. Grajales-Nishimura, R. A. F. Grieve, S. P. S. Gulick, K. R. Johnson, W. Kiessling, C. Koeberl, D. A. Kring, K. G. MacLeod, T. Matsui, J. Melosh, A. Montanari, J. V. Morgan, C. R. Neal, D. J. Nichols, R. D. Norris, E. Pierazzo, G. Ravizza, M. Rebolledo-Vieyra, W. U. Reimold, E. Robin, T. Salge, R. P. Speijer, A. R. Sweet, J. Urrutia-Fucugauchi, V. Vajda, M. T. Whalen, P. S. Willumsen, The Chicxulub asteroid impact and mass extinction at the Cretaceous-Paleogene boundary. *Science* **327**, 1214–1218 (2010).

59. D. M. Raup, Large-body impact and extinction in the Phanerozoic. *Paleobiology* **18**, 80–88 (1992).

60. D. A. Kring, Reevaluating the impact cratering kill curve. *Meteorit. Planet. Sci.* **37**, 1648–1649 (2002).

61. C. R. Chapman, D. Morrison, Impacts on the Earth by asteroids and comets: Assessing the hazard. *Nature* **367**, 33–40 (1994).

62. K. F. Kuiper, A. Deino, F. J. Hilgren, W. Krijgsman, P. R. Renne, J. R. Wijbrans, Synchronizing rock clocks of Earth history. *Science* **320**, 500–504 (2008).

63. A. Brumm, G. M. Jensen, G. D. van den Bergh, M. J. Morwood, I. Kurniawan, F. Aziz, M. Storey, Hominins on Flores, Indonesia, by one million years ago. *Nature* **464**, 748–752 (2010).

64. T. A. Rivera, M. Storey, C. Zeeden, F. J. Hilgen, K. Kuiper, A refined astronomically calibrated $^{40}Ar/^{39}Ar$ age for Fish Canyon sanidine. *Earth Planet. Sci. Lett.* **311**, 420–426 (2011).

65. G. G. Kenny, I. Mänttäri, M. Schmieder, M. J. Whitehouse, A. A. Nemchin, J. J. Bellucci, R. E. Merle, Age of the Sääksjärvi impact structure, Finland: Reconciling the timing of small impacts in crystalline basement with regional basin development. *J. Geol. Soc.* **177**, 1231–1243 (2020).

66. R. M. Hazen, L. W. Finger, Crystal structure and compressibility of zircon at high pressure. *Am. Mineral.* **64**, 196–201 (1979).

67. I. Farnan, E. Balan, C. J. Pickard, F. Mauri, The effect of radiation damage on local structure in the crystalline fraction of $ZrSiO_4$: Investigating the ^{29}Si NMR response to pressure in zircon and reidite. *Am. Mineral.* **88**, 1663–1667 (2003).

68. C. J. Howard, R. J. Hill, B. E. Reichert, Structures of ZrO_2 polymorphs at room temperature by high-resolution neutron powder diffraction. *Acta Cryst. B* **44**, 116–120 (1988).

69. M. J. Whitehouse, B. S. Kamber, Assigning dates to thin gneissic veins in high-grade metamorphic terranes: A cautionary tale from Akilia, southwest Greenland. *J. Petrol.* **46**, 291–318 (2005).

70. M. Wiedenbeck, P. Allé, F. Corfu, W. L. Griffin, M. Meier, F. Oberli, A. von Quadt, J. C. Roddick, W. Spiegel, Three natural zircon standards for U-Th-Pb, Lu-Hf, trace element and REE analyses. *Geostand. Newsl.* **19**, 1–23 (1995).

71. L. P. Black, S. L. Kamo, C. M. Allen, D. W. Davis, J. N. Aleinikoff, J. W. Valley, R. Mundil, I. H. Campbell, R. J. Korsch, I. S. Willians, C. Foudoulis, Improved $^{206}Pb/^{238}U$ microprobe geochronology by the monitoring of a trace-element-related matrix effect; SHRIMP, ID–TIMS, ELA–ICP–MS and oxygen isotope documentation for a series of zircon standards. *Chem. Geol.* **205**, 115–140 (2004).

72. R. H. Steiger, E. Jäger, Subcommission on geochronology: Convention on the use of decay constants in geo- and cosmochronology. *Earth Planet. Sci. Lett.* **36**, 359–362 (1977).

73. J. S. Stacey, J. D. Kramers, Approximation of terrestrial lead isotope evolution by a two-stage model. *Earth Planet. Sci. Lett.* **26**, 207–221 (1975).

74. K. R. Ludwig, Isoplot 3.75 A geochronological toolkit for Microsoft Excel (Berkeley Geochronology Center Spec. Publ. No. 5, 2012).

75. F. M. Gradstein, J. G. Ogg, M. D. Schmitz, G. M. Ogg, *Geologic Time Scale 2020* (Elsevier, 2020).

76. T. Westerhold, N. Marwan, A. J. Drury, D. Liebrand, C. Agnini, E. Anagnostou, J. S. K. Barnet, S. M. Bohaty, D. De Vleeschouwer, F. Florindo, T. Frederichs, D. A. Hodell, A. E. Holbourn, D. Kroon, V. Lauretano, K. Littler, L. J. Lourens, M. Lyle, H. Pälike, U. Röhl, J. Tian, R. H. Wilkens, P. A. Wilson, J. C. Zachos, An astronomically dated record of Earth's climate and its predictability over the last 66 million years. *Science* **369**, 1383–1387 (2020).

77. A. E. Pickersgill, D. F. Mark, M. R. Lee, S. P. Kelley, D. W. Jolley, The Boltysh impact structure: An early Danian impact event during recovery from the K-Pg mass extinction. *Sci. Adv.* **7**, eabe6530 (2021).

78. C. Porter, P. Morin, I. Howat, M.-J. Noh, B. Bates, K. Peterman, S. Keesey, M. Schlenk, J. Gardiner, K. Tomko, M. Willis, C. Kelleher, M. Cloutier, E. Husby, S. Foga, H. Nakamura, M. Platson, M. Wethington Jr., C. Williamson, G. Bauer, J. Enos, G. Arnold, W. Kramer, P. Becker, A. Doshi, C. D'Souza, P. Cummens, F. Laurier, M. Bojesen, ArcticDEM (Harvard Dataverse, V1, 2018); https://doi.org/10.7910/DVN/OHHUKH.

79. R. L. Shreve, Movement of water in glaciers. *J. Glaciol.* **11**, 205–214 (1972).

80. P. R. Renne, K. B. Knight, S. Nomade, K. N. Leung, T. P. Lou, Application of deuteron-deuteron (D-D) fusion neutrons to $^{40}Ar/^{39}Ar$ geochronology. *Appl. Radiat. Isot.* **62**, 25–32 (2005).

81. K. Min, R. Mundil, P. R. Renne, K. R. Ludwig, A test for systematic errors in $^{40}Ar/^{39}Ar$ geochronology through comparison with U/Pb analysis of a 1.1-Ga rhyolite. *Geochim. Cosmochim. Acta* **64**, 73–98 (2000).

82. J.-Y. Lee, K Marti, J. P. Severinghaus, K. Kawamura, H.-S. Yoo, J. B. Lee, J. S. Kim, A redetermination of the isotopic abundances of atmospheric Ar. *Geochim. Cosmochim. Acta* **70**, 4507–4512 (2006).
